# Recombinant *Plasmodium vivax* circumsporozoite surface protein allelic variants: antibody recognition by individuals from three communities in the Brazilian Amazon

**DOI:** 10.1038/s41598-020-70893-3

**Published:** 2020-08-20

**Authors:** Isabela Ferreira Soares, César López-Camacho, Rodrigo Nunes Rodrigues-da-Silva, Ada da Silva Matos, Barbara de Oliveira Baptista, Paulo Renato Rivas Totino, Rodrigo Medeiros de Souza, Kate Harrison, Alba Marina Gimenez, Elisângela Oliveira de Freitas, Young Chan Kim, Joseli Oliveira-Ferreira, Cláudio Tadeu Daniel-Ribeiro, Arturo Reyes-Sandoval, Lilian Rose Pratt-Riccio, Josué da Costa Lima-Junior

**Affiliations:** 1 Laboratório de Imunoparasitologia, Instituto Oswaldo Cruz, Fundação Oswaldo Cruz, (Fiocruz), Rio de Janeiro, RJ Brazil; 2 Nuffield Department of Medicine, The Jenner Institute, The Henry Wellcome Building for Molecular Physiology, University of Oxford, Oxford, UK; 3 Laboratório de Tecnologia em Anticorpos Monoclonais, Instituto de Tecnologia de Imunobiológicos, Fiocruz, Rio de Janeiro, Brazil; 4 Laboratório de Pesquisa em Malária, Instituto Oswaldo Cruz, Fundação Oswaldo Cruz, Rio de Janeiro, Brazil; 5 Centro de Pesquisa em Doenças Infecciosas, Centro Multidisciplinar, Campus Floresta, Universidade Federal do Acre, Rio Branco, Brazil; 6 Centro de Pesquisa, Diagnóstico e Treinamento em Malária, Fiocruz, Rio de Janeiro, RJ Brazil

**Keywords:** Malaria, Protein vaccines

## Abstract

Circumsporozoite protein (CSP) variants of *P. vivax*, besides having variations in the protein repetitive portion, can differ from each other in aspects such as geographical distribution, intensity of transmission, vectorial competence and immune response. Such aspects must be considered to *P. vivax* vaccine development. Therefore, we evaluated the immunogenicity of novel recombinant proteins corresponding to each of the three *P. vivax* allelic variants (VK210, VK247 and *P. vivax*-like) and of the C-terminal region (shared by all PvCSP variants) in naturally malaria-exposed populations of Brazilian Amazon. Our results demonstrated that PvCSP-VK210 was the major target of humoral immune response in studied population, presenting higher frequency and magnitude of IgG response. The IgG subclass profile showed a prevalence of cytophilic antibodies (IgG1 and IgG3), that seem to have an essential role in protective immune response. Differently of PvCSP allelic variants, antibodies elicited against C-terminal region of protein did not correlate with epidemiological parameters, bringing additional evidence that humoral response against this protein region is not essential to protective immunity. Taken together, these findings increase the knowledge on serological response to distinct PvCSP allelic variants and may contribute to the development of a global and effective *P. vivax* vaccine.

## Introduction

*Plasmodium*, a complex multi-stage organism, has specialized proteins that promote the parasite’s survival in both vertebrate and invertebrate hosts and support the invasion of multiple cell types. Vaccines targeting sporozoites correspond to an attractive strategy widely explored since fewer than 100 sporozoites are inoculated in human host during the blood meal of infected female Anopheles mosquito^1^. Moreover, it was already demonstrated that in mice^2^, non-human primates^1,2^ and humans^3^, the immunization using irradiated sporozoites is capable to elicit protective immunity.

Despite the broad investigation of various proteins as vaccine candidates, the circumsporozoite surface protein (CSP) remains in the lead because it was described as sporozoites’ major surface protein and it has a crucial role in sporozoite’s motility and hepatocyte invasion^4,5^. It has been reported that the CSP play a vital role in invading to the mosquito's salivary glands, binding sporozoite to liver cells, and inactivating the host cell protein synthesis machinery^6^. It has already been demonstrated that specific antibodies against the *P. falciparum* circumsporozoite protein (PfCSP), present in the serum of vaccinated mice, rhesus macaques and humans, have the ability to block sporozoite invasion of hepatocytes^7^. Structurally, CSP contains approximately 400 amino acids and is organized into three domains: The N-terminal, which contain the conserved pentapeptide (region I); a highly repetitive species-specific central domain (repetitive region) and a conserved C-terminal domain (region II).

Currently, the most advanced malaria vaccine is the RTS,S, manufactured by GlaxoSmithKline (GSK). RTS,S, produced in *Saccharomyces cerevisiae,* consists in a recombinant vaccine comprising PfCSP’s C-terminal and repeat regions in combination with hepatitis B virus’ surface antigen (S)^8^. This already licensed vaccine is being implemented since 2018 in selected areas of Ghana, Kenya and Malawi^9,10^. On the other hand, such as almost all vaccine candidates currently tested, this vaccine targets only *P. falciparum*. The priority given to falciparum malaria results from the ability of this plasmodial species in producing elevated parasite loads and to invade red blood cells in all stages, causing extensive morbidity and mortality^11^. Nevertheless, some particularities of *P. vivax,* such as the increasing numbers of reports of severe vivax malaria^12–15^ and the appearance of strains resistant to treatment^16–18^, highlight the importance of the development of a specific vaccine against *P. vivax*.

In contrast to PfCSP, *P. vivax* CSP (PvCSP) is polymorphic and has three allelic variants. Analyses in PvCSP genotypes demonstrated the existence of sequence repeats of this protein belonging to one of two types of nonapeptide repeat units known as VK210 (GDRA(A/D)GQPA) and VK247 (ANGA(G/D)(N/D)QPG)^19,20^. In addition, a third variant, identified by Qary et al.^21^ as *P. vivax*-like, is different from both nonapeptide variants, and it is composed by a repeat sequence of 11-mer-APGANQ(E/G)GGAA-. Phylogenetic and serological study conducted by Souza-Neiras et al.^22^, has demonstrated that differences in these three variants are strictly present in the central repeats of the protein, but present several nucleotide variations with important serological impact. Therefore, it should be considered for PvCSP vaccine trials once they represent important intra-specific biological signatures. In Brazil, the prevalence of PvCSP allelic variants was previously studied and is known in several states such as Acre, Amazonas, Belém, Macapá, Mato Grosso, Pará, Porto Velho and Rondônia^23,24^.

Although less investigated than PfCSP, *P. vivax* CSP began to receive more attention in recent years and strategies were developed to overcome the variations in central domain. An example is the *Escherichia coli* expressed vaccine VMP001, which encodes a chimeric CSP and contains sequences with repetitions of alleles VK210 and VK247. This vaccine went into clinical testing and was capable of inducing recognition, agglutination and virulence loss of live sporozoites, due to the high levels of antibodies induced^25^. Salman et al. also reported the deployment of a highly protective *P. vivax* vaccine, composed by Salvador I sequences of CSP, including its C-terminal region and central repeats of VK210 and VK247, on the surface of a virus-like particle (VLP) based on the Hepatitis B surface antigen, overall known as Rv21. This vaccine was used in rodent model challenges with transgenic sporozoites, where it was capable to achieve 100% sterile protection^26^. In the present study, the naturally acquired humoral immune response to PvCSP repeat variants was evaluated in exposed populations of three regions in the Brazilian Amazon, using recombinant proteins for each one of the three alleles already described. We have also determined the antibody subclass profile induced by different PvCSP variants and verified the associations between the specific IgG and its subclasses (IgG1, IgG2, IgG3 ad IgG4) with epidemiological characteristics that can suggest exposition and/or protection indicatives in exposed populations.

## Results

### Epidemiological profile of the studied population

299 individuals living in three different endemic areas of Brazilian Amazon composed our study population (Cruzeiro do Sul, Guajará and Mâncio Lima). The population age ranged from 12 to 88 years old (median 32 years) and presented similar frequencies of female and male individuals. Studied individuals have been naturally exposed to malaria infection, have been living in endemic areas for 31 years (ranging from 3 to 88 years), most frequently in the same address (0 to 88 years, median 20 years) and reporting, for 86% of the population, at least one previous malaria episode. *P. vivax* was the most prevalent species, together this species mono-infections and mixed infections corresponded to 65.2% of cases diagnosed during the period of study (p < 0.0001), and to the leading cause of previous malaria episodes in 81% of studied individuals. The control group composed by 53 individuals from the non-endemic area of Rio de Janeiro, who never reported malaria episodes, was composed by 69.8% female and 30.2% male, ranging from 17 to 43 years old (median 20 years). The analysis of individual populations based on localities studied (Table 1) reveals a similar profile in relation to time of exposure, number of past malaria infections and diagnosis at the time of blood collection. However, volunteers residing in Guajará (GJ) presented longer time since the last malaria episode (median 12 months) than those of Cruzeiro do Sul (median 2.5 months) and Mâncio Lima (median 3.5 months; p = 0.0044 and 0.0001, respectively).

**Table 1:** Epidemiological features of the study population.

Epidemiological features	CZS^a^	GJ^b^	ML^c^	Total
	(n = 124)	(n = 87)	(n = 88)	(n = 299)
**Gender—N (%)**				
Male	65 (52.4%)	45 (51.7%)	44 (50%)	154 (51.5%)
Female	59 (47.6%)	42 (48.3%)	44 (50%)	145 (48.5%)
**Malaria exposure—median (IR)**				
A.P.I	55,5	42,5	107,2	61,4
Age (years)	29.5 (20–45)	33 (22–50)	33.5 (23–42.5)	32 (22–47)
Years of residence on endemic area	29 (19.5–44)	33.5 (23–50)	32.5 (21–43)	31 (21–47)
Years of residence in the present address	20 (6–36)	18 (3–29)^c^*	27.5 (12.5–38.5)^b^*	20 (5–36)
Months since the last malaria episode	2.5 (0–60)^b^**	12 (4–48)^a^**^/c^***	3.5 (0–22)^b^***	5 (0–36)
Number of malaria episodes on the last year	1 (0–1)	0 (0–1)	1 (0–1.5)	0.5 (0–1)
Number of previous malaria episodes	6 (2–12)	5 (2–10)	10 (5–20)	7 (3–15)
**Species causing previous episodes—N (%)**				
*P. vivax*	23 (19%)	16 (18%)	17 (19%)	56 (19%)
*P. falciparum*	10 (8%)	6 (7%)	0 (0%)	16 (5%)
*P. vivax and P. falciparum*	68 (55%)	51 (59%)	67 (76%)	186 (62%)
Never infected	1 (0.8%)	6 (7%)	0 (0%)	7 (2%)
Not reported	21 (17%)	8 (9%)	5 (6%)	34 (11%)
**Diagnosis—N (%)**				
*P. vivax*	39 (31.5%)	10 (11.5%)	24 (27.3%)	73 (24.4%)
*P. falciparum*	25 (20.2%)	6 (6.9%)	10 (11.4%)	41 (13.7%)
Mixed	0 (0%)	1 (1.1%)	3 (3.4%)	4 (1.3%)
Negative	60 (48.4%)^b^**	70 (80.5%)^a^**^/c^**	51 (58%)^b^**	181 (60.5%)

Values of A.P.I. (annual parasitic index), Age, Years of residence in endemic areas, Years of residence in the present address, Months since the last malaria, Number of malaria episodes on the last year and number of previous malaria episodes represent the median (interquartile range). Frequencies were compared by Fisher’s test, and other epidemiological parameters were compared by Mann–Whitney test. Upper scripted letters (^a,b,c^) indicate the studied populations (CZS = Cruzeiro do Sul; ML = GJ = Guajará and Mâncio Lima, respectively) and statistical differences were represented by * (*)p < 0.05; (**)p < 0.005; ***p < 0.0005.

### Design of the vCSP proteins and assessment of protein secretion for purification

**Figure 1:**
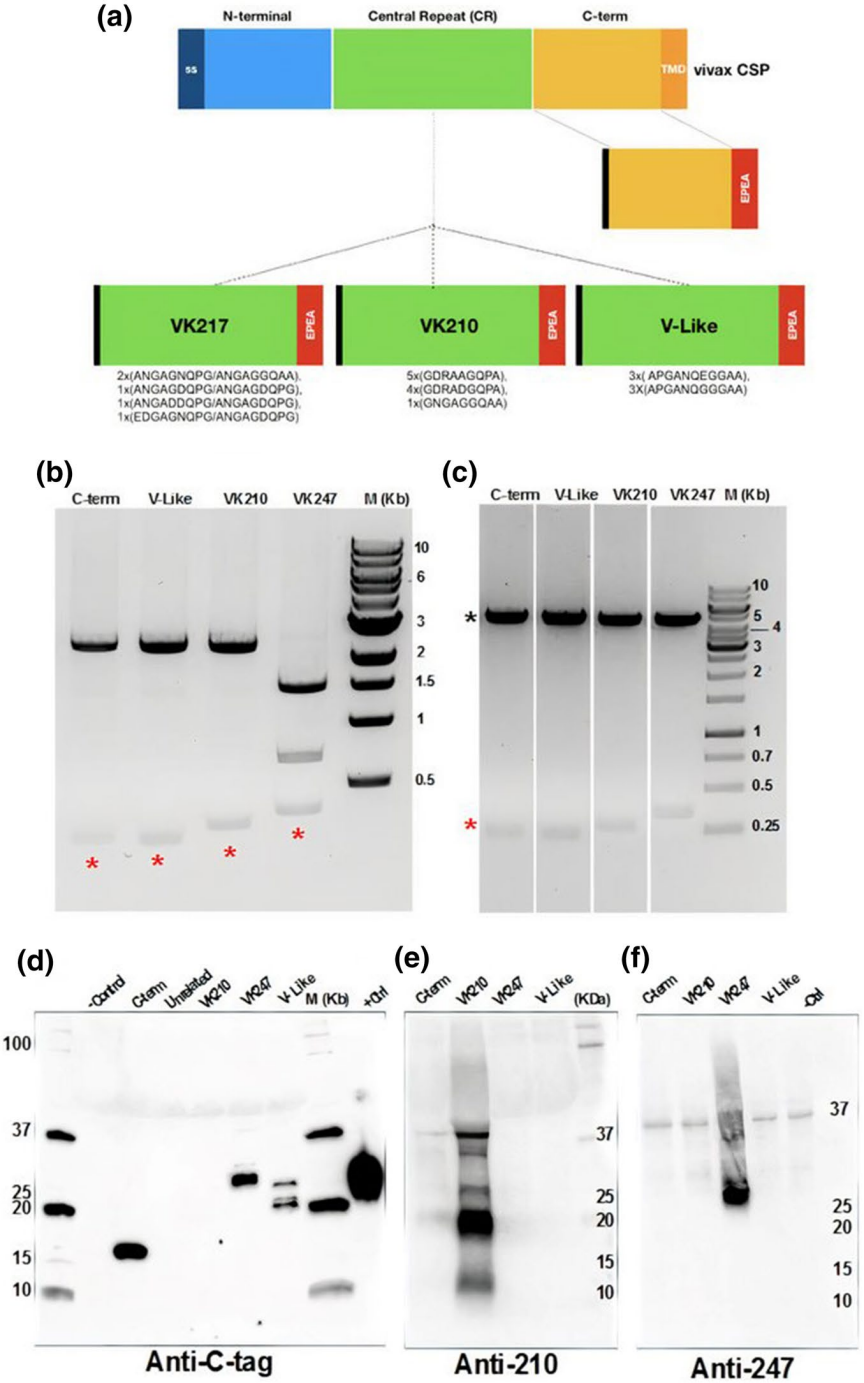
Expression of vCSP malarial antigens for protein production. (**a**) Schematic representation of the vivax CSP protein. The C-term portion (yellow) without its transmembrane domain, as well as the 3 different allelic variants from the central repeat region (VK217, VK247 and V-like) were fused to the c-tag epitope (EPEA) to allow purification using a c-tag affinity column (red). Black color represents the exogenous signal sequence contained in the expression plasmid construct. (**b**) Synthetic DNA sequences carrying such malarial antigens were extracted by double enzymatic digestion (AgeI and KpnI), red asterisk represents the specific size for each construct that were cut from the agarose gel, followed by ligation to the backbone plasmid pHLsec. (**c**) Enzymatic restriction from the PhLsec plasmids after ligation with the malarial antigens. Red asterisk represents the specific size of the coding regions and black asterisk represents the PhLSec backbone. PhLSec plasmid were transfected into HEK293 cells and supernatant was recovered 4 days after transfection. 10 ml of each of the supernatants was subjected to SDS-PAGE and western blot against the anti-C-Tag (**d**), the anti-210 (**e**), and the anti-247 (**f**) antibodies.

Serological analysis of the immune response to malarial antigens is paramount to establish the immunogenicity of potential vaccine candidates and the immune competence or even the effect of parasite genetic polymorphisms in the immunity of residing populations in a given geographical location. However, most of the studies conduct ELISAS against the full-length protein of interest. Here we sought to dissect the serological immune responses against vivax CSP malaria by constructing expression plasmids encoding the sole central repeats of each of the allelic variants of VK247, VK210 and V-Like, respectively, and the C-terminal region of CSP (Fig. 1). The design of these sub-domain regions is based in the antigenic conformation of the chimeric CSP 210/247 from the Rv21 vaccine^26^ which also contains the highly conserved C-Terminal region of vCSP. For the case of the Vivax-Like repeats we used a sequence previously reported^26^. Figure 1a shows a diagrammatic representation of vCSP and the four subunits that were synthesized and enzyme-digested to ligate (Fig. 1b) into the expression plasmid PhLSec. After ligation with the PhLsec backbone, bacterial transformants were double-digested to verify the right size of the transgenes (Fig. 1c). The red and black asterisks denote the specific size of each transgene and the PhLSec backbone, respectively. Upon confirmation of the right clones by enzymatic digestion, plasmids were further verified by Sanger-sequencing. PhLsec plasmids were then transfected into HEK293 cells and supernatant was recovered to assess the secretion capabilities of our c-tagged vCSP subunits. Supernatants were subjected to western blot analysis using a camelid anti-C-Tag antibody (Fig. 1d). Specific bands were detected for the C-term, VK247 and Vivax-Like as well as in the unrelated NS1 beta ladder protein fused to EPEA (c-tag). No bands were detected in the negative untransfected cells or in the unrelated PhLsec plasmid fused to His-tag. However, the anti-C-Tag antibody failed to recognize the VK210 repeats, suggesting a masking effect inflicted by the protein-resolving conditions. To further investigate this masking effect, we used specific monoclonal antibodies (mAb) targeting the VK210 (Fig. 1e) and the VK247 (Fig. 1f) repeats, respectively. By using the anti-210 mAb, we detected a strong signal in the sample that was negative in the C-tag western blot, and not in the other transfectant supernatants, thus confirming the secretion capabilities of VK210 repeats (Fig. 1e). On the other hand, when using the anti-247 antibody, we detected abundant signal in the lane corresponding to the VK247 supernatants, thus confirming the secretion of the VK247 repeats (Fig. 1f). A non-specific band of 35 Kda was detected in all samples. Therefore, the design of the expression plasmids allows the expression and the secretion of the vCSP subunits for a subsequent protein-column purification, to be used as coating agents in the ELISA assays.

### Frequency and magnitude of IgG antibodies to recombinant proteins derived from PvCSP

**Figure 2:**
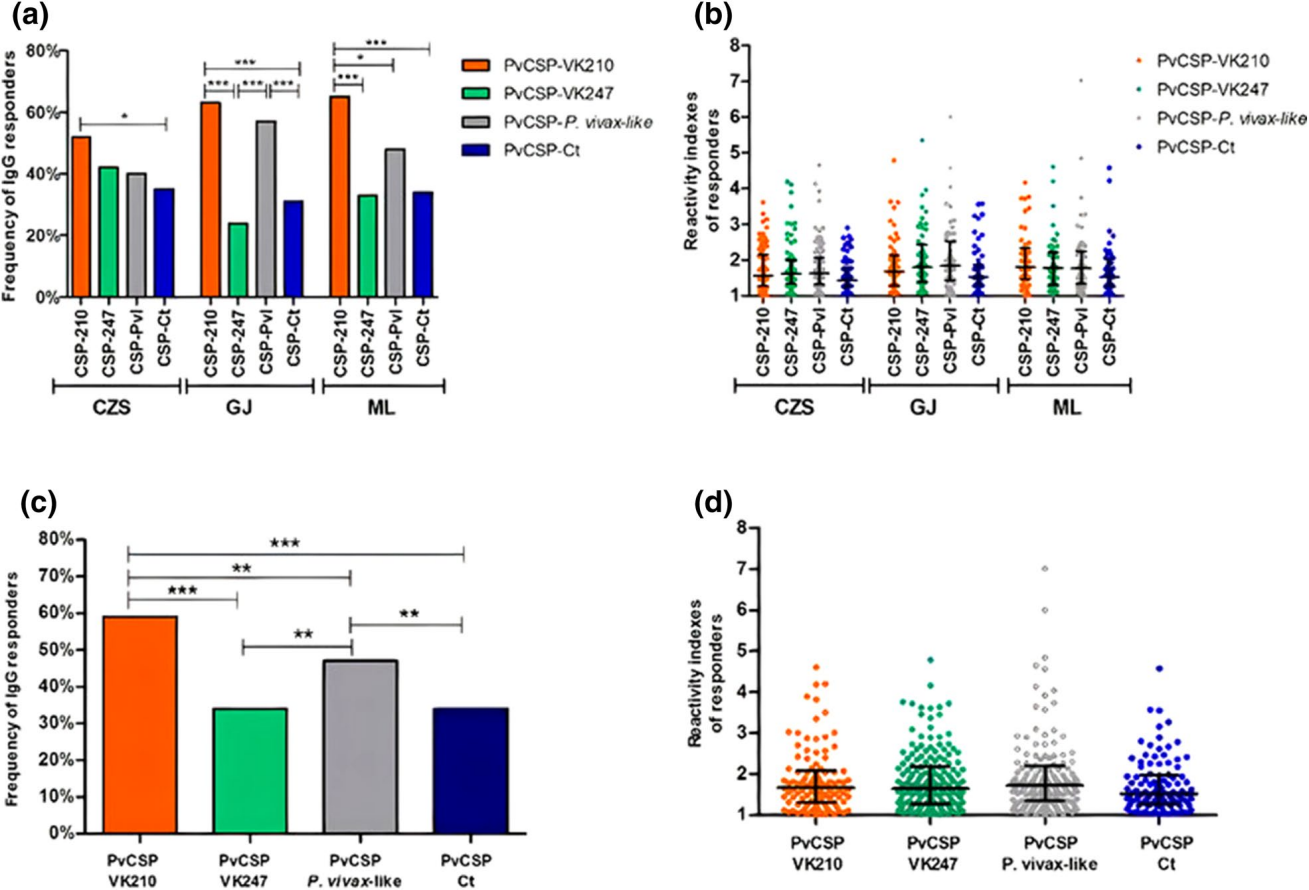
Humoral response against *Pv*CSP recombinant proteins. Frequencies of IgG responders in each studied locality (**a**); reactivity indexes of responders in the three studied localities (**b**); overall frequencies of IgG responders (**c**) and overall reactivity indexes of IgG antibodies of responders (**d**) to each recombinant protein derived from *Pv*CSP. In (**a**) and (**c**), each bar represents the frequency of responders to one of the recombinant proteins (*Pv*CSP-VK210: orange bar; PvCSP-VK247: green bar; *Pv*CSP-*P.vivax-like*: gray bar and *Pv*CSP-Ct: purple bar). Figure 1b,d, each point represents an individual RI against *Pv*CSP recombinant proteins (*Pv*CSP-VK210: orange points; *Pv*CSP-VK247: green points; *Pv*CSP-*P.vivax-like*: gray points and *Pv*CSP-CT: purple points). Black bars on (**b**) and (**d**) represent values of median and interquartile range. RIs higher than 1 are considered responders to the recombinant protein. Frequencies of responders were compared by Fisher´s exact test and RIs were compared by Mann–Whitney test. Significant differences were indicated by *. (*) p < 0.05; (**) p < 0.005; ***p  < 0.0005.

We assessed the naturally acquired IgG response against PvCSP recombinant central repeats of PvCSP-VK210, PvCSP-VK247, and PvCSP-*P. vivax*-like, as well as the PvCSP-C terminal region, in 299 exposed individuals. Firstly, we evaluated the frequency of responders to recombinant PvCSPs in each studied community (Fig. 2a). In Cruzeiro do Sul (CZS), the frequencies of responders to PvCSP allelic variants (PvCSP-VK210, PvCSP-VK247, PvCSP-*P. vivax*-like) were quite similar (52%, 42% and 40%, respectively), while in Guajará (GJ) we observed higher frequencies of responders to PvCSP-VK210 (63%) and PvCSP-*P. vivax*-like (57%) than frequencies of responders to PvCSP-VK247 (24%) (p < 0.0001 and p < 0.0001, respectively) and to PvCSP-Ct (31%) (p < 0.0001 and p = 0.0007, respectively). Moreover, in Mâncio Lima community (ML), we observed a prevalence of responders to PvCSP-VK210 (65%) when compared to frequency of responders to PvCSP-VK247 (33%, p < 0.0001), PvCSP-*P.vivax*-like (48%, p = 0.0331) and to PvCSP-Ct (34%, p < 0.0001). In relation to the magnitude of response to different recombinant PvCSP among responders, despite differences in epidemiological data and frequencies of responders in each studied location, similar IgG reactivity indexes against PvCSP variants and PvCSP-Ct were observed in all studied populations (Fig. 2b). Based on the absence of statistical differences among IgG reactivity indexes against recombinant proteins in these three studied places, we decided to analyze them as a single population. Responders to PvCSP-VK210 (n = 177; 59%) were more prevalent when compared to responders to PvCSP-VK247 (n = 102, 34%; p < 0.0001), to PvCSP-P. vivax-like (n = 141, 47%; p = 0.0041) and to PvCSP-Ct (n = 101, 34%; p < 0.0001), followed by responders to PvCSP-P. vivax-like, which were more prevalent than those responding to PvCSP-VK247 (p = 0.0015) and to PvCSP-Ct (p = 0.0011) (Fig. 2c). Despite differences observed in frequencies of IgG responders, the magnitude of IgG responses was similar among responders to PvCSP recombinant proteins. The average RI of responders against PvCSP-VK210 (1.84 ± 0.72), PvCSP-VK247 (1.86 ± 0.77), PvCSP-P. vivax-like (1.96 ± 0.95) and PvCSP-Ct (1.74 ± 0.65) did not differ statistically (p > 0.05) (Fig. 2d). All samples from healthy Control group were negative to all four recombinant proteins.

**Figure 3:**
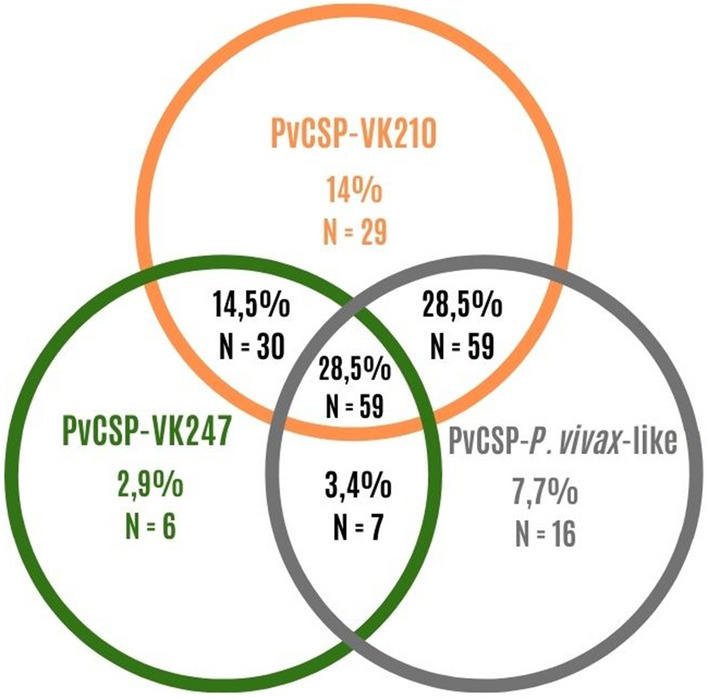
Venn diagram of responders to at least one of the recombinant proteins. In this diagram, values of N and the percentage of responders are demonstrated. Values marked with colors represent individuals that respond exclusively to the indicated protein (orange *Pv*CSP-VK210, green *Pv*CSP-VK247 and gray *Pv*CSP-*P. vivax*-like). Values in black represent individuals that respond to two or three of the recombinant proteins according to the circles in which values are inserted.

Two hundred and seven of individuals (69%) presented IgG antibodies against at least one of recombinant proteins, from these 92% (190) have reported previous *P. vivax* infections, including ongoing infections 23,7% (49). Only 17 individuals never reported previous *P. vivax* episodes and 4 of these (2%) were infected with *P. falciparum.* Among responders, 16.9% of individuals presented antibodies against all four recombinant proteins, 37.7% against three recombinant proteins, 25.6% to two of the recombinant proteins and 19.8% to only one antigen. Inside the group of responders to only one recombinant protein (n = 41), PvCSP-VK210 represented 56.1%. Moreover, focusing on humoral immune response to PvCSP variants, 28.5% of responders to at least one of recombinant proteins, responds to the three PvCSP-variants, 14.5% to PvCSP-VK210 and PvCSP-VK247, 28.5% to PvCSP-VK210 and PvCSP-*P. vivax*-like, 3.4% to PvCSP-VK247 and PvCSP-*P. vivax*-like, 14% responds only to PvCSP-VK210, 2.9% only to PvCSP-VK247 and 7.7% only to PvCSP-*P. vivax*-like, as demonstrated in Fig. 3.

In order to evaluate if the infection status could change the antibody profile of recognition, we also assessed the frequency and magnitude of antibodies against the recombinants by the presence/absence of *P. vivax* at the time of blood collection. Among individuals living in endemic areas, 31% (n = 92) did not present antibodies to any of the recombinant proteins, these individuals include 28 infected *P. vivax* individuals. We have also selected *P. vivax* infected and non-infected individuals inside the group of 207 recombinant protein responders and compared their frequencies and IgG reactivity indexes. We found out higher frequencies of IgG responders to PvCSP-VK210 and PvCSP-Ct in non-infected individuals (p = 0.0086 and p = 0.0117, respectively). However, IgG magnitude was similar between these two groups (supplementary figure S1).

### Evaluation of IgG subclass profile against PvCSP variants

**Figure 4:**
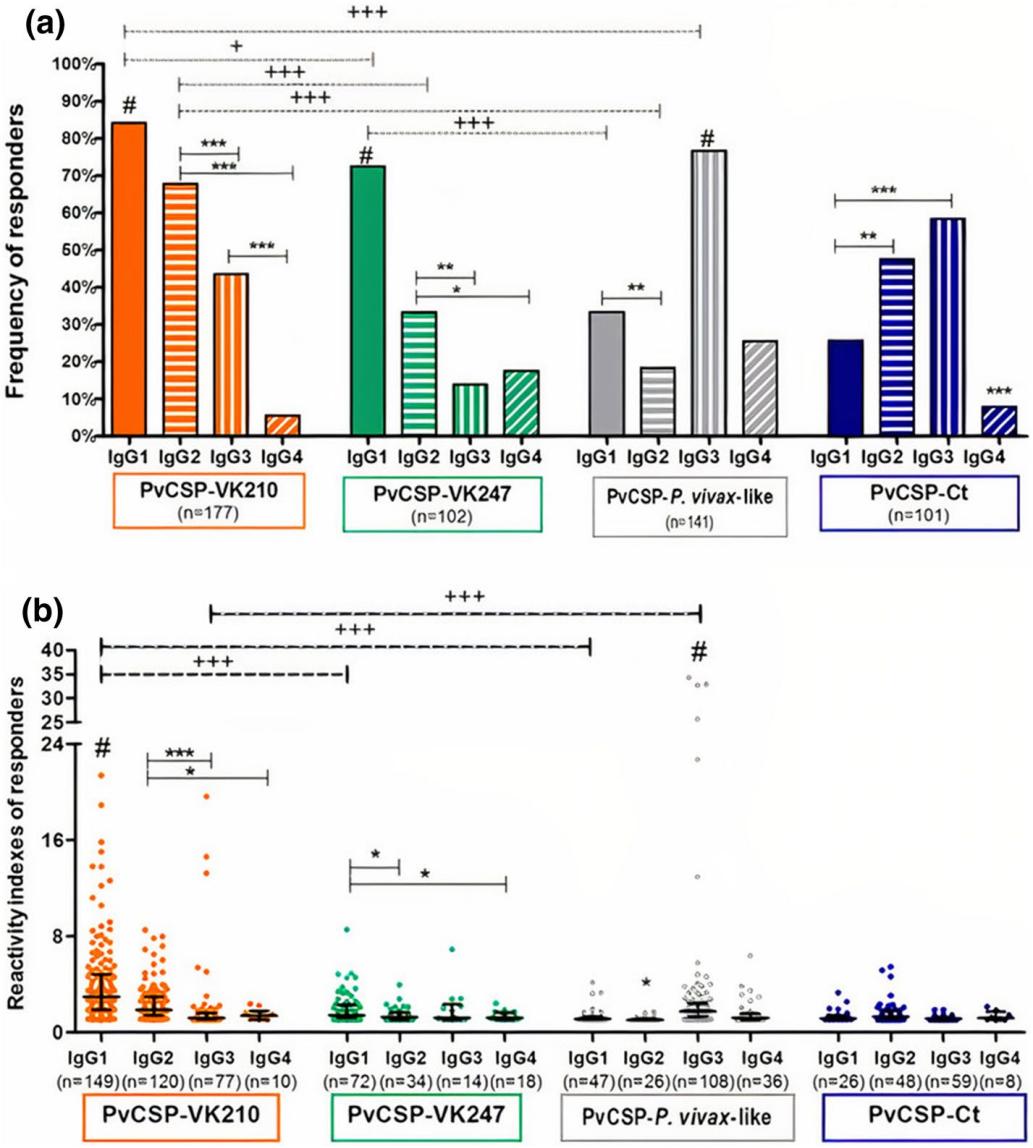
Frequency of IgG subclass responders (**a**) and their reactivity indexes (**b**) against the recombinant *Pv*CSP proteins in the studied population. Points represent an individual RI against PvCSP recombinant proteins (*Pv*CSP-VK210: orange points; *Pv*CSP-VK247: green points; *Pv*CSP-*P. vivax-like*: gray points and *Pv*CSP-CT: purple points). The red traced line represents the cutoff, RIs higher than 1 are considered responders to the recombinant protein. Frequencies of responders were compared by Fisher´s exact test and RIs were compared by Mann–Whitney test. Significant differences among subclasses were indicated by * and significant differences among responsiveness to each protein were indicated by + ; (*) p < 0.05; (**) p < 0.01; (***; +  + +) p < 0.001. In this figure # means that the subclass is different from all the others against the same protein.

We assessed the overall subclass distribution of the IgG antibody (IgG1, IgG2, IgG3, IgG4) responses among responders to each recombinant protein. Among 177 responders to PvCSP-VK210, 149 individuals (84.2%) presented IgG1 antibodies. This is higher than the frequencies of IgG2 (67.8%, p = 0.0005), IgG3 (43.5%, p < 0.0001) and IgG4 (5.6%, p < 0.0001). Similarly, responders to PvCSP-VK247 (n = 102), also had a prevalence of IgG1 (70.6%) over the other IgG subclasses (p < 0.0001), followed by IgG2 (33.3%) and similar frequencies of IgG3 (13.7%) and IgG4 (17.6%) (p = 0.0015 and p = 0.0155, respectively, when compared to IgG2) responders. In contrast, among responders to PvCSP-*P. vivax-*like (n = 141), IgG3 was the most frequent subclass, representing 76.6% of responders (p < 0.0001 as compared to other subclasses), while 33.3%, 19% (p = 0.0063) and 25.5% of this group presented IgG1, IgG2 and IgG4 specific antibodies respectively. Lastly, among responders to the C-terminal region of PvCSP (PvCSP-Ct, n = 101), we observed a minor frequency of responders to IgG4 (7.9%) than to all other subclasses (p < 0.0001). Moreover, similar frequencies of responders to IgG3 (58.4%) and IgG2 (47.5%) were observed, both of them, higher than the observed frequency of responders to IgG1 (25.7%) (p < 0.0001 and p = 0.0021, respectively) (Fig. 4a).

Concerning the magnitude of response of each IgG subclass against PvCSP, the RIs ranged from 0.15 to 34.32. Focusing on subclasses profile against PvCSP-VK210, IgG1 responders presented higher RIs (median = 2.97; interquartile range = 1.86–4.83) than IgG2 (median = 1.86; interquartile range = 1.39–2.93, p < 0.0001), IgG3 (median = 1.2; interquartile range = 1.1–1.6, p < 0.0001) and IgG4 responders (median = 1.39; interquartile range = 1.07–1.79, p = 0.0002), while responders to IgG3 and to IgG4 presented similar RI and both subclasses had lower RIs than IgG2 (p < 0.0001 and 0.018, respectively). Moreover, despite the high prevalence of IgG1 responders against PvCSP-VK247, the RIs of IgG1 responders (median = 1.43; interquartile range = 1.2–2.28) were only higher than IgG2 (median = 1.28; interquartile range = 1.07–1.66; p = 0.0359) and IgG4 (median = 1.23; interquartile range = 1.08–1.69; p = 0.0436), although RIs of IgG3 responders (median = 1.2; interquartile range = 1.07–2.34) were similar to other IgG subclasses. Besides, concerning the reactivity to PvCSP-*P. vivax*-like, the RIs of IgG3 responders (median = 1.76; interquartile range = 1.31–2.38) were higher than that of IgG1 (median = 1.15; interquartile range = 1.09–1.3, p < 0.0001), of IgG2 (median = 1.04; interquartile range = 1.02–1.15, p < 0.0001) and of IgG4 (median = 1.23; interquartile range = 1.05–1.54, p < 0.0002). Finally, considering the responsiveness to PvCSP-C-terminal region, we observed no differences among the RIs of responders to IgG1 (median = 1.16; interquartile range = 1.08–1.39); IgG2 (median = 1.29; interquartile range = 1.12–1.82); IgG3 (median = 1.15; interquartile range = 1.06–1.25) and IgG4 (median = 1.18; interquartile range = 1.06–1.73).

Furthermore, a higher IgG1 RI response against PvCSP-VK210 was observed when compared to RI of IgG1 against PvCSP-VK247 (p < 0.0001) and to PvCSP-*P.vivax-like* (p < 0.0001), and a higher IgG3 reactivity to PvCSP-*P.vivax*-like than to PvCSP-VK210 (p < 0.0001) (Fig. 4b). When we selected and compared infected and non-infected responders to each subclass per recombinant protein we did not find differences in frequencies and reactivity indexes (supplementary figure S1).

### Evaluation of associations between exposition/protection factors and humoral immune response to PvCSP

**Figure 5:**
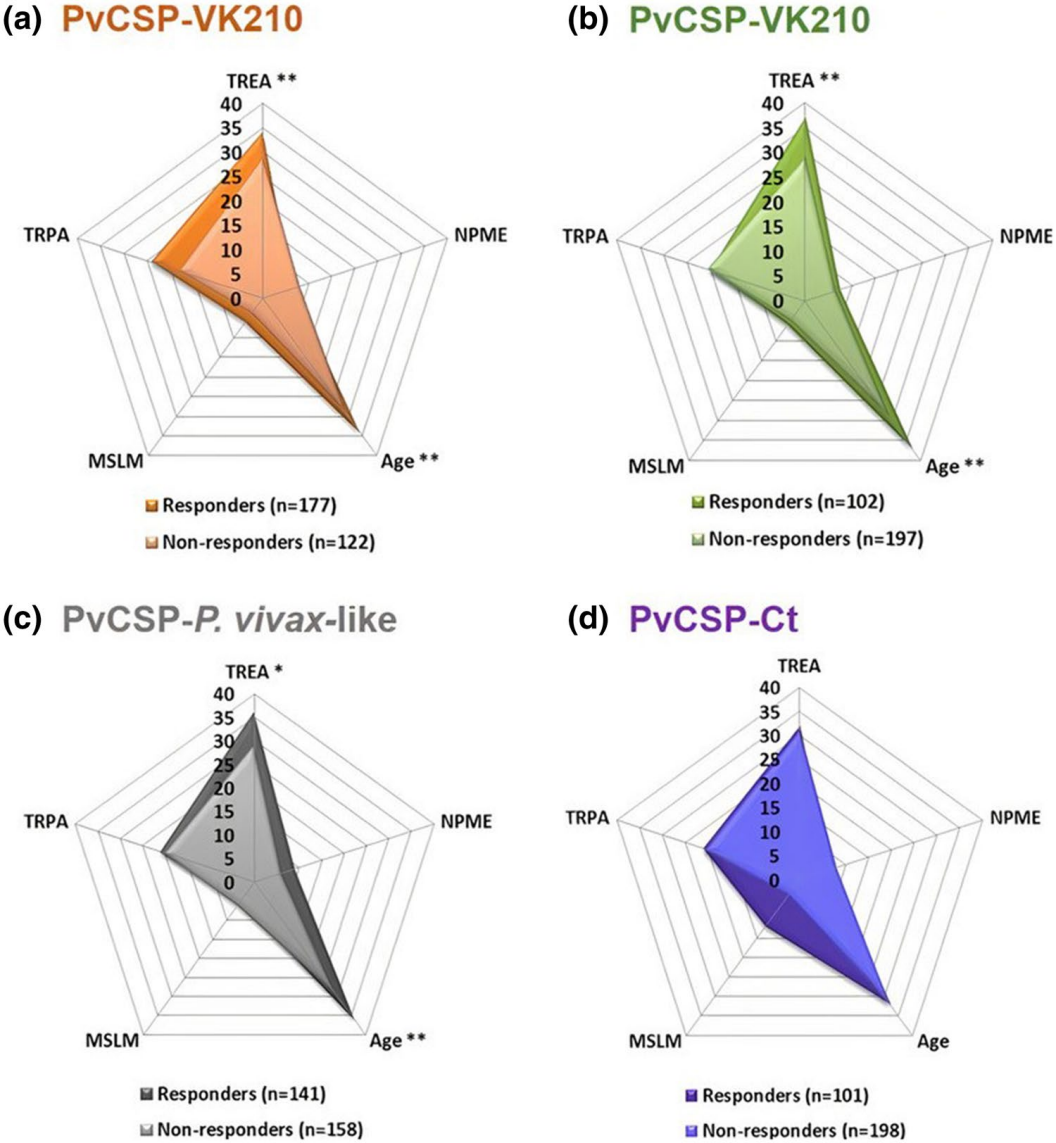
Comparison of epidemiological parameters between responders and non-responders to each *Pv*CSP recombinant protein. TREA = Time of residence in endemic area (years), NPME = Number of previous malaria episodes, MSLM = Months since last malaria and TRPA = Time of residence in the present address (years). Epidemiological data of responders and non-responders to each one of the recombinant proteins were compared by Mann–Whitney test.

In order to investigate the influence of epidemiological parameters on the responsiveness to PvCSP, we compared the epidemiological data of responders and non-responders to each one of the PvCSP recombinant proteins. Responders to PvCSP-VK210, VK247 and *P. vivax-like* presented higher median of age (p = 0.007; p = 0.003; p = 0.007, respectively) and time of residence in endemic area (p = 0.005; p = 0.005 and p = 0.015, respectively) than non-responders to each protein. No statistical difference was found between responders and non-responders to PvCSP-Ct (Fig. 5a–d).

To identify factors associated with the magnitude of response to each antigen, we investigated the existence of correlations between RIs against recombinant proteins and epidemiological data. Firstly, we observed a tendency of correlation between age and time of residence on endemic area with RIs of IgG antibodies against PvCSP-VK210 (p = 0.007, r = 0.159 and p = 0.005, r = 0.165; respectively) and against PvCSP-*P. vivax-like* (p = 0.026, r = 0.131 and p = 0.037, r = 0.124; respectively). In addition, RIs of IgG antibodies against PvCSP-VK247 presented a tendency of correlation with the number of previous malaria episodes (NPME) (p = 0.024, r = 0.135).

Besides, searching for associations between subclass profile against PvCSP-variants and epidemiological data, we identified that the number of previous malaria episodes positively correlated with the IgG1 levels to PvCSP-VK210 (p = 0.008, r = 0.204) and IgG3 levels to PvCSP-VK247 (p = 0.0007, r = 0.278). For PvCSP-*P.vivax-like* responders, the RIs of IgG2 were directly correlated with both, age (p = 0.002, r = 0.266) and time of residence in endemic area (p = 0,001, r = 0.294). No significant correlations were observed between epidemiological data and humoral response to PvCSP-Ct.

### Characteristics of responders to the three major PvCSP variants

**Figure 6:**
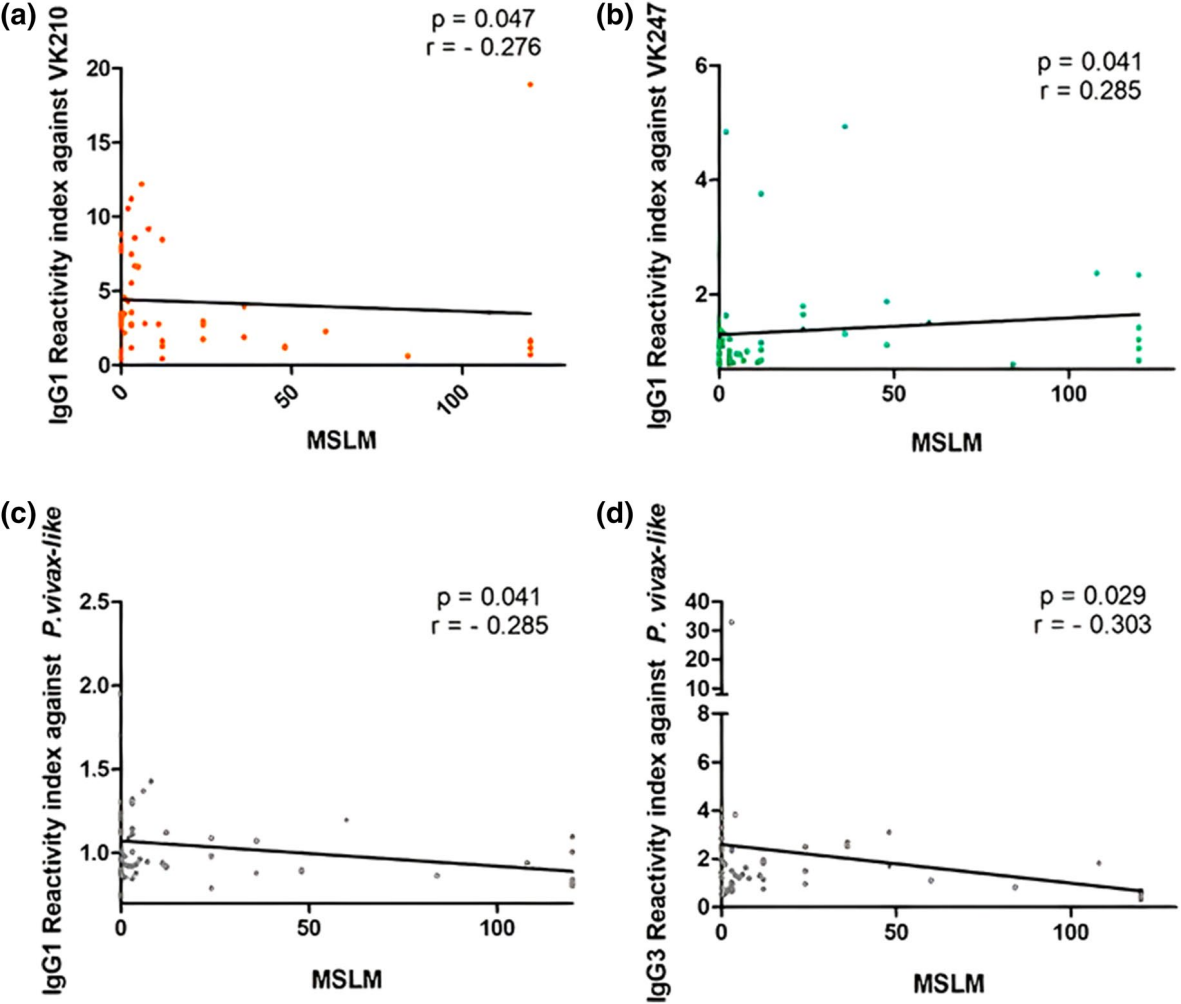
Correlation of RIs of cytophilic IgG subclasses with months since the last malaria (MSLM). (**a**) Correlation of IgG1 RIs against *Pv*CSP-VK210 with MSLM. (**b**) Correlation of IgG1 RIs against *Pv*CSP-VK247 with MSLM. (**c**) Correlation of IgG1 RIs against *Pv*CSP-*P. vivax-like* with MSLM. (**d**) Correlation of IgG3 RIs against *Pv*CSP-*P. vivax-like* with MSLM.

In order to investigate the effect of responsiveness against all PvCSP variants on protection against malaria, we compared the epidemiological data of responders to all three PvCSP variants (VK210, VK247 and *P. vivax-like*; N = 59) with those of individuals who did not respond to at least one of these variants (N = 240). Samples of responders to three variants have a significantly longer time of residence in endemic area (TREA) (median = 38; interquartile range = 24.5–52.5), on average 6 years more than individuals that did not respond (median = 30; interquartile range = 21–44) (p = 0.017). Moreover, the RIs of cytophilic antibodies of responders to three PvCSP variants inversely correlated with months since the last malaria (MSLM). Reactivity indexes of IgG1 against PvCSP-VK210 (p = 0.047; r = − 0.276), IgG1 (p = 0.041; r = − 0.285) and IgG3 (p = 0.029; r = − 0.303) against PvCSP-*P. vivax-like* inversely correlated with MSLM (Fig. 6a,c,d). However, as it is shown in Fig. 6b, RIs of IgG1 against PvCSPVK247 directly correlated with this same parameter (p = 0.041; r = 0.285).

## Discussion

Previous works have proposed that CSP variants of *P. vivax*, besides having variations in the repetitive portion of the protein, can differ from each other in aspects such as geographical distribution, intensity of transmission, vectorial competence, immune response, response to treatment and drug resistance^23,27–29^. Such aspects must be considered to *P. vivax* vaccine development^22^. Therefore, multidisciplinary studies characterizing PvCSP variants epidemiology may advance in the development of an effective vaccine against *P. vivax*.

In our study, we evaluated and characterized the humoral IgG response to the PvCSP variant repeats (VK210, VK247 and *P. vivax*-like) and the C-terminal region, in a population consisting of 299 individuals naturally exposed to *P. vivax* malaria, living in Acre, a state of the malaria endemic Brazilian Amazon. From 207 responders to at least one of recombinant proteins, 190 described previous *P. vivax* infections and just 49 were infected with this species*.* This result suggests that production of antibodies against PvCSP extends for past infections. This feature was previously demonstrated in a study conducted by Longley et al. at a low transmission region in Thailand, where the majority of study population was not infected with *P. vivax* but still presented detectable IgG levels^30^. Other study of a yearlong cohort also demonstrated IgG positivity and magnitude persistence over 1 year period in absence of qPCR-detectable blood stage *P. vivax* infections^31^. Our results demonstrated that, PvCSP-VK210 presented the highest prevalence of responders, followed by PvCSP-*P. vivax-like* and PvCSP-VK247. Frequency profiles were already described in different Amazon regions and they are consistent with our results. Oliveira-Ferreira et al. have observed the same profile of IgG responders to these PvCSP variants (More responders to PvCSP-VK210, followed by PvCSP-*P. vivax-like* and PvCSP-VK247) in Candeias do Jamari in Rondônia state, in 2004^32^. However, the mentioned paper found lower frequencies in comparison to our study, which could be related to the use of synthetic peptides as antigens in their ELISA assays. In 2000, Machado and Póvoa evaluated the distribution of PvCSP variants of *P. vivax* from three endemic areas (Belém, Macapá and Porto Velho), and again found a dominance of prevalence similar to ours^33^. Years later, Storti-Melo et al. assessed the frequency of PvCSP variants using PCR/RFLP samples from five states of the Brazilian Amazon, Acre, Amapá, Mato Grosso, Pará and Rondônia. Again the profile of distribution of the variants was similar to ours^34^. Among facts that may explain observed prevalences, we highlight the distinct susceptibility of *Anopheles* mosquitoes to different PvCSP variants, which depends on the recognition of specific ligands of the peritrophic matrix by ookinete surface proteins, triggering migration or entrapment of the parasite^35^. A study that used dissected blood fed infected mosquitoes and ELISA assays, demonstrated a preferential development of PvCSP-VK210, as compared to that of PvCSP-VK247, in *An. aquasalis* and *An. darlingi*, in the state of Pará^27^. Moreover, frequencies of IgG responders to PvCSP recombinant proteins corroborate the co-circulation of all three *P. vivax* variants in Amazon region^23,24,36^. Besides, the higher frequency of antibodies to VK210 are in according to studies that described this variant as the most common in Amazon, while VK247 was rarely reported as single infection^22,23,34^. In our study we found 28 *P. vivax* infected patients that are non-responders to recombinant proteins and this was already described in literature. In 2018, Oliveira-Ferreira performed a study to evaluate seroprevalence to CSP and demonstrated that not only Brazilian naturally exposed individuals but also *P. vivax* infected ones, were unresponsive to peptides of this protein. In this same study, two specific allelic groups were associated with absence of antibodies against PvCSP (HLA-DRB1*01 and HLA-DQB1*05)^37^. In this scenario, we believe our findings can be explained by well documented associations of the immune response with specific HLA alleles against malaria antigens^38,39^.

Concerning the functionality of the IgG subclasses, we did observe a predominance of cytophilic antibodies to PvCSP variants, once that IgG1 was the prevailing subclass against VK210 and VK247, and IgG3 was the principal IgG subclass of antibodies to *P. vivax*-like. Interestingly, this preponderance of cytophilic antibodies was previously described to PvCSP and PvTRAP, suggesting that this profile could be associated to the adaptive immune response against preerythrocyte stage proteins^40^. Moreover, in places where malaria is endemic and the local population has had several malaria episodes through the years, there are evidences that the premonition (immune condition characterizing clinical protection as reflected by the absence of fever and presence of infection at low densities of parasitemia) is reached after repeated infections, due to the presence of both cytophilic antibodies (IgG1 and IgG3) and memory cells^41–43^. In this context, despite that we not found any significant association between cytophilic antibodies and protection in this study, we believe that these antibodies could act in synergy with other antibodies against preerythrocytic targets, promoting a protective effect. However, it is important to highlight that previous works have already demonstrated heterologous reactivity occurring in antibodies elicited against CSP antigens of *P. vivax* and *P. falciparum*^44^, so, despite the fact that the majority of our recombinant protein responders got in touch with *P. vivax,* we can’t affirm that these antibodies are exclusively to *P. vivax.*

For the purpose of elucidating the influence of epidemiological factors in humoral response to PvCSP variants and C-terminal region, we correlated induced RI’s of IgG and its’ subclasses with the studied population parameters. We found out that IgG reactivity indexes of PvCSP-VK210 and PvCSP-*P. vivax-like* presented a trend to increase with time of exposition to infection. This tendency could be explained by both, the preferential development of PvCSP-VK210 in predominant species of anopheles mosquitoes in Amazon and the widely geographical distribution of these variants when compared to PvCSP-VK247 (suggested to have a lower geographic adaptation in Brazil)^27,34^. These data may explain why people living longer in these endemic regions tend to have higher IgG reactivity indexes against PvCSP-VK210 and PvCSP-*P. vivax-like*, since they get in touch more frequently with these two variants. On the other hand, IgG reactivity indexes of PvCSP-VK247 presented a tendency of correlation with the number of previous malaria episodes, which is also coherent with the aforementioned condition, since individuals that have more infections will probably have more chances to get in touch with this less geographically distributed variant. In our results we have considered three areas as a single one. However, looking separately to these areas, Mâncio Lima and Cruzeiro do Sul demonstrate a similar/higher prevalence of the variant PvCSP-VK247 on PvCSP-*P. vivax-like* as a previous work has demonstrated in other Brazilian endemic areas^24^.

We have then checked possible correlations between subclass reactivity indexes and epidemiological parameters. Our findings suggest a cumulative effect on IgG1 reactivity indexes against PvCSP-VK210 as a result of previous malaria episodes. On the other hand, PvCSP-VK247 IgG3 RI’s presented a direct correlation with the NPME. Differently of the PvCSP variants, the RI’s of IgG and its’ subclasses able to recognize the C-terminal region of the protein were not able to correlate with any of the epidemiological parameters described. These findings are consistent with those reported by Arévalo-Herrera describing high levels of cytophilic antibodies capable to recognize fragments from both PvCSP N-terminal and repeated regions, based on immunizations with long synthetic peptides in phase I clinical assays, while the C-terminal region was not immunogenic in humans^45^. In addition, despite immunogenic in mice, N or C terminals only (i.e. lacking either VK210 or 247 repeat sequences) also failed to induce protective immunity^26^. Since Kurtovic et al. pointed complement activation by antibodies as an important mechanism of anti-sporozoite human immunity, one of the possible mechanisms that might explain the non-immunogenic profile of PvCSP-Ct is by means of the complement fragment C3d, capable to limit the anti-CSP C-terminal flanking sequence-specific antibody response by masking epitopes in this region of the molecule. Thus, C3d binding to CSP C-terminal region may represent a mechanism based on the exploitation of the innate immune response by the parasite in order to suppress the development of an acquired immune response against a conserved region of the protein^46^.

In agreement with the previous discussed results, comparison of epidemiological parameters of responders and non-responders to each one of the recombinant proteins presented statistical differences of age and TREA for the three PvCSP allelic variants. Again, only PvCSP-Ct did not present such a difference and induced low responses in natural exposure conditions. This finding could suggest that the production of specific antibodies against the C-terminal region is not conditioned by the TREA or parasite exposure. In fact, the production of antibodies to PvCSP-Ct may be related to the ability of each individual's immune system to circumvent a possible mechanism of parasite-mediated evasion, like we previously suggested.

From all of the studied population (299) individuals, 59 presented specific IgG antibodies to all of PvCSP variants. When compared to individuals that did not respond to at least one of the variants, these individuals presented significantly longer TREA (mean of 6 years). Furthermore, this same group presents inverse correlations between cytophilic RI’s (IgG1 against PvCSP-VK210; IgG1 and IgG3 against PvCSP-*P. vivax-like*) and MSLM, suggesting that these specific antibodies could, somehow, represent markers of recent infection. On the other hand, only IgG1 RI’s of PvCSP-VK247 were directly correlated with MSLM, implying that these specific antibodies may be important for a protective immune response.

In conclusion, despite the limitations in sample size and study design, our work indicates that IgG positivity and magnitude against PvCSP variants can persist from past infections in the studied population, even in the absence of an ongoing malaria episode. The profile of responders to PvCSP allelic variants and its geographical distribution are still compatible with scientific literature, with dominance of PvCSP-VK210, followed by PvCSP-*P. vivax-like* and PvCSP-VK247. The immune response raised against the recombinant proteins studied here, were mediated, predominantly, by cytophilic antibodies, which have a relevant role to trigger a protective immune response. Conversely, C-terminal region, does not seem to be the best option in vaccine design due its low-immunogenic properties and absence of correlation with epidemiological parameters and protection indicatives. Lastly, taking into account the limitations of the number of individuals enrolled in our study and the unique features of studied population, which is historically exposed to *P. vivax* and *P. falciparum* simultaneously, we can not exclude the possibility of heterologous reactivity of antibodies against *P. vivax* CSP variants. Therefore, other epidemiological studies regarding *P. vivax* allelic variants in other areas are encouraged and essential to address this question and increase the knowledge about the serological landscapes in endemic regions, which is fundamental to develop a global *P. vivax* vaccine.

## Methods

### Study area and volunteers

The cross-sectional cohort study included 299 individuals from three different communities with malaria transmission in Acre state: Cruzeiro do Sul (n = 124), Guajará (n = 87) and Mâncio Lima (n = 88). Samples of 53 individuals living in non aendemic areas of Rio de Janeiro and never exposed to malaria were the control group. Samples and survey data were collected from June to August of 2016. Informed consent was obtained from all donors by written declaration. The study was reviewed and approved by the Fundação Oswaldo Cruz Ethical Committee and the National Ethical Committee of Brazil.

### Epidemiological survey

To evaluate the potential influence of clinical and exposure features on immunity response against PvCSP variants, the donors were interviewed prior the blood sampling. Questions related to personal exposure to malaria, such as time of residence in transmission areas, previous malária episodes, use of prophylactic measures, symptoms and personal knowledge of malaria transmission were done with all study participants. The answers were stored in Epi-Info databank for subsequent analysis (Centers for Disease Control and Prevention, Atlanta, GA, USA). Written informed consent was obtained from all adult donors or from parents of donors in the case of minors.

### Malaria diagnosis and blood sampling

Blood samples were collected by venipuncture in heparin tubes and centrifuged (350×*g*, 10 min) to plasma separation and storage at − 20 °C to ship to Laboratório de Imunoparasitologia, Fiocruz—RJ. Thin and thick blood smears were stained and analyzed for malaria parasites. The diagnosis were done by examination of 200 fields at 1,000× magnifications under oil-immersion and two research experts in malaria diagnosis examined all slides. Individuals positive for *P. vivax* and/or *P. falciparum* at the time of blood collection were treated using the chemotherapeutic regimen recommended by the Brazilian Ministry of Health.

### Recombinant PvCSP variants expression in HEK-293 T cells

As previously described by Longley et al.^31^, the *P. vivax* sequences used were *Pv*CSPVK210 of Belem strain (GenBank accession number P08677), *Pv*CSPVK247 of Papua New Guinea (GenBank accession number M69059), *Pv*CSPVivax-like of Papua New Guinea (GenBank accession number L13724.1) and *Pv*CSP C-terminal region from Salvador I strain (NCBI Reference Sequence XP_001613068.1). Each one of the domains contained in the multi-variant chimeric recombinant protein used in the Rv21 vaccine described by Salman et al.^26^ was synthesized and codon-optimized using the Geneart service (ThermoFisher), as follows;

C-terminal: NNEGANAPNEKSVKEYLDKVRATVGTEWTPCSVTCGVGVRVRRRVNAANKKPEDLTLNDLETDVCTMDK.

VK210: (5x(GDRAAGQPA), 4x(GDRADGQPA), 1x(GNGAGGQAA)); VK247: (2x(ANGAGNQPG/ANGAGGQAA), 1x(ANGAGDQPG/ANGAGDQPG), 1x(ANGADDQPG/ANGAGDQPG), 1x(EDGAGNQPG/ANGAGDQPG)).

Finally, Vivax-like repeats, with a sequence 3x(APGANQEGGAA), 3x(APGANQGGGAA), was obtained from the genebank accession number: AAA18616.

DNA fragments encoding each of the vCSP regions were cloned in the expression vector pHLsec, which is flanked by the chicken β-actin/rabbit β-globin hybrid promoter with a signal secretion sequence and with a modification of a C-tag instead of a His-tag tail.

For expression and purification of vCSP malarial antigens, the codon-optimized coding regions were cloned into the pHLsec vector, as previously described^47^, which is flanked by the chicken β-actin/rabbit β-globin hybrid promoter with a signal secretion sequence and a C-tag (EPEA). The pHLsec plasmids (500 μg) were transfected in HEK-293 cells using polyethyleneimine (PEI) in roller bottles (surface area of 2,125 cm^2^) under standard cell culture conditions. Five days after transfection, cells were discarded and media was filtered through 0.22 μM disposable filters. The secreted protein was purified from the supernatant by affinity chromatography (C-tag column), using the Äkta Start chromatography system and eluted with 2.0 M MgCl_2_ and 20 mM Tris, pH 7.0. Finally, the eluted protein was dialysed using Slide-A-LyzerTM cassette (Fisher Scientific) against 1× PBS.

### Western Blot

Supernatants from transfected HEK293 cells were boiled at 100 °C for 5 min in laemli buffer. Equal amounts cell supernatants were resolved by SDS/PAGE and transferred to PVDF membranes. Blots were blocked with 1× PBS-Tween- 5% milk and incubated with an anti-C-Tag antibody (CaptureSelect™ Biotin Anti-C-tag Conjugate at 1:2,000 dilution, supernatants from a MRA-185 hybridoma cell line (2E10.E9) that recognize the VK247 repeats, and supernatants from a MRA-184 hybridoma cell line (2F2) that recognize the VK210 repeats. followed by incubation with HRP-conjugated secondary antibody (1:5,000). Chemiluminescence (Perkin-Elmer Life Sciences, Boston, MA) was visualized using the BioRad ChemiDoc SRS device.

### Antibody assays

Anti-PvCSP specific antibodies against recombinant VK210, VK247, Vivax-like and C terminal region (CT), were evaluated by enzyme-linked immunosorbent assay (ELISA) as previously described by Matos et al.^48^. Briefly, MaxiSorp 96-well plates (Nunc, Rochester, NY, USA) were coated overnight with 1.0 µg/ml of each recombinant protein. Plates were washed and blocked for 1 h at 37 °C. After blocking, plasma samples (1:100 in PBS-Tween-BSA 5%) were incubated in duplicate wells during 1 h. After three washing steps, bound antibodies were detected with anti-human IgG-PE (Sigma, St. Louis) and followed by addition of o-phenylenediamine and hydrogen peroxide. Plates were read at 492 nm using a SpectraMax 250 ELISA reader (Molecular Devices, Sunnyvale, CA, USA). The results for total IgG were expressed as reactivity indexes (RIs), which were calculated by the mean optical density (O.D.) of an each tested sample divided by the cut-off, number expressed by the mean optical density of 10 non-exposed control individuals’ samples plus 3 standard deviation (cut-offs values: VK210 = 0.208, Vivax-like = 0.268, VK247 = 0.260 and Ct = 0.245). These 10 non-exposed control individuals’ corresponded to 10 higher DO’s found in 53 control individuals tested (VK210 = 0.126; 0.131; 0.137; 0.143; 0.146; 0.146; 0.149; 0.153; 0.183; 0.184; Vivax-like = 0.147; 0.175; 0.185; 0.189; 0.191; 0.202; 0.210; 0.210; 0.220; 0.230; VK247 = 0.139; 0.146; 0.149; 0.168; 0.172; 0.178; 0.188; 0.193; 0.215; 0.220; Ct = 0.129; 0.131; 0.137; 0.148; 0.156; 0.182; 0.183; 0.189; 0.192; 0.196). Subjects were scored as responders to PvCSP variants if the RI of IgG against each one of the recombinant proteins was higher than 1. Additionally, the RIs of IgG subclasses for each one of the proteins, were evaluated on responders of each PvCSP variant. The same method was done, using peroxidase-conjugated goat anti-human IgG1, IgG2, IgG3, and IgG4 (Sigma, St. Louis) (cut-offs—VK210 IgG1 = 0.158, IgG2 = 0.168 , IgG3 = 0.194 and IgG4 = 0.315, Vivax-like IgG1 = 0.060, IgG2 = 0.053, IgG3 = 0.110 and IgG4 = 0.065, VK247 IgG1 = 0.234, IgG2 = 0.337, IgG3 = 0.288 and IgG4 = 0.439 and Ct IgG1 = 0.161, IgG2 = 0.308, IgG3 = 0.191 and IgG4 = 0.284).

### Statistical analysis

Statistical analyzes were done in GraphPad Prism 5.0 for Windows (GraphPad Software, Inc.). Normality test were done in all variables using the one-sample Kolmogorov–Smirnoff test. The Dunn’s test was used to compare RIs of IgG against recombinant PvCSP variants in studied groups. Uncorrected Fisher’s plus LSD was done to access the differences in proportions of IgG, IgG subclass. Correlations between immune response and epidemiological parameters were evaluated by Spearman rank test. A two-sided p value < 0.05 was considered significant.

### Ethics approval and consent to participate

Written consent for use of plasma samples and survey data were obtained in accordance to the revised Declaration of Helsinki. Both collection and consent protocols were under approval of Fundação Oswaldo Cruz Ethical Committee and the National Ethical Committee of Brazil (CEP-FIOCRUZ CAAE 46084015.1.0000.5248).

## Supplementary information

Supplementary Information 1.

Supplementary Information 2.

## Data Availability

The present manuscript includes all datasets generated for this study.
