# Supplementary material for: Recombinant Plasmodium vivax circumsporozoite surface protein allelic variants: antibody recognition by individuals from three communities in the Brazilian Amazon
Source: Sci Rep. 2020 Aug 20;10:14020. doi: 10.1038/s41598-020-70893-3 (PMC7441389; doi:10.1038/s41598-020-70893-3)
Supplement: Supplementary file 1 — Supplementary Information 1. [file 41598_2020_70893_MOESM1_ESM.pdf]

Recombinant *Plasmodium vivax* Circumsporozoite Surface Protein allelic variants: antibody recognition by individuals from three communities in the Brazilian Amazon

Isabela Ferreira Soares[#1], César López-Camacho[#2], Rodrigo Nunes Rodrigues-da-Silva[#3], Ada da Silva Matos[1], Barbara de Oliveira Baptista[4], Paulo Renato Rivas Totino[4], Rodrigo Medeiros de Souza[5], Kate Harrison[2], Alba Marina Gimenez[2], Elisângela Oliveira de Freitas[2], Young Chan Kim[2], Joseli Oliveira-Ferreira[1], Cláudio Tadeu Daniel-Ribeiro[4,6], Arturo Reyes-Sandoval[2], Lilian Rose Pratt-Riccio[4], Josué da Costa Lima-Junior[1]

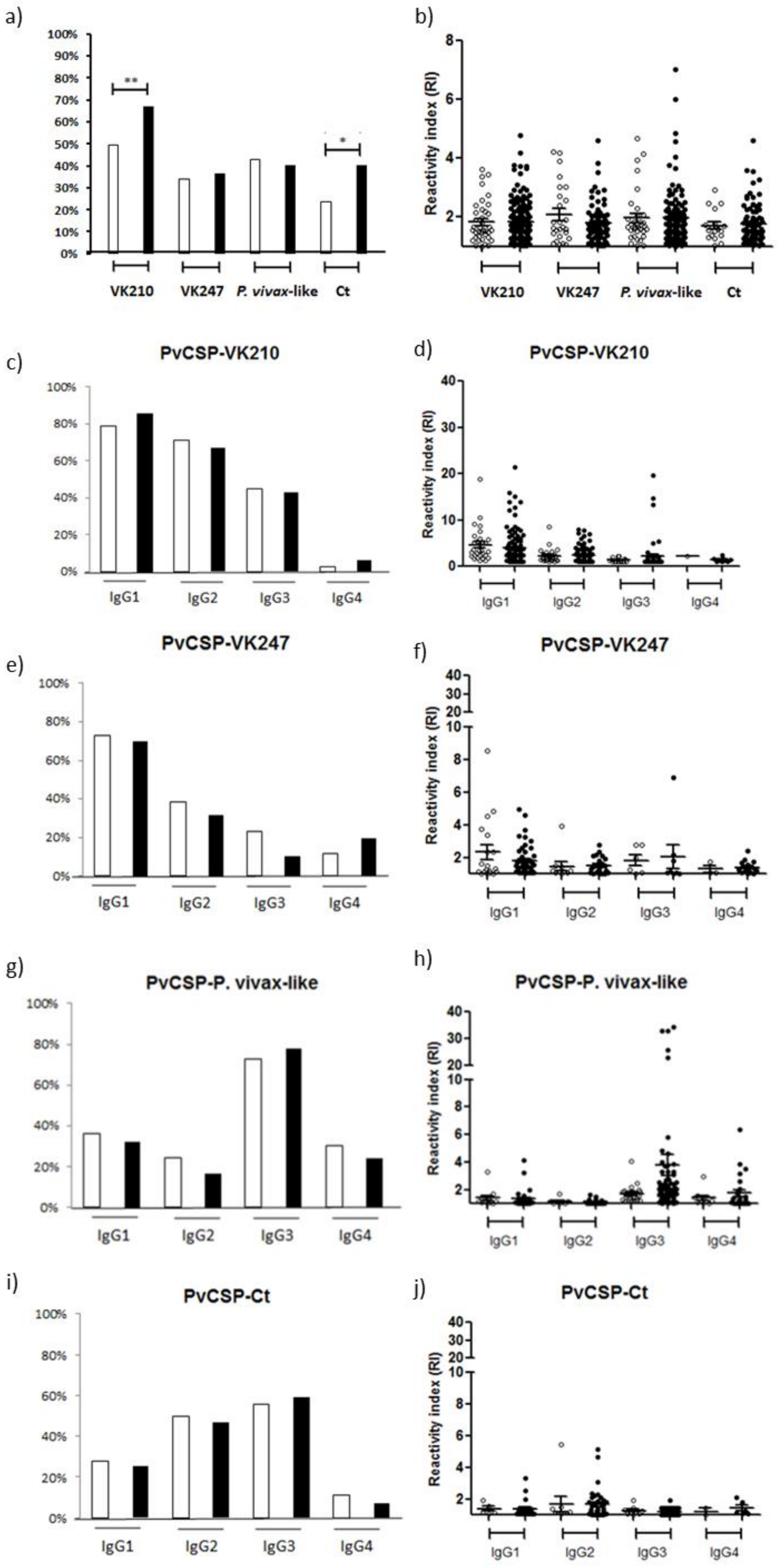

a)
100%
90%
80%
70%
60%
50%
40%
30%
20%
10%
0%
**
*
VK210
VK247
P. vivax-like
Ct
b)
Reactivity index (RI)
8
6
4
2
VK210
VK247
P. vivax-like
Ct
c)
PvCSP-VK210
100%
80%
60%
40%
20%
0%
IgG1
IgG2
IgG3
IgG4
d)
PvCSP-VK210
Reactivity index (RI)
40
30
20
10
0
IgG1
IgG2
IgG3
IgG4
e)
PvCSP-VK247
100%
80%
60%
40%
20%
0%
IgG1
IgG2
IgG3
IgG4
f)
PvCSP-VK247
Reactivity index (RI)
40
30
20
10
8
6
4
2
IgG1
IgG2
IgG3
IgG4
g)
PvCSP-P. vivax-like
100%
80%
60%
40%
20%
0%
IgG1
IgG2
IgG3
IgG4
h)
PvCSP-P. vivax-like
Reactivity index (RI)
40
30
20
10
8
6
4
2
IgG1
IgG2
IgG3
IgG4
i)
PvCSP-Ct
100%
80%
60%
40%
20%
0%
IgG1
IgG2
IgG3
IgG4
j)
PvCSP-Ct
Reactivity index (RI)
40
30
20
10
8
6
4
2
IgG1
IgG2
IgG3
IgG4

Supplementary figure 1: Frequencies and RI’s of IgG responders and subclasses separated by infected and non-infected individuals. White bars and white circles represent infected individuals while dark bars and dark circles represent non-infected individuals. Figures (a) and (b) compare frequencies and RI’s of infected and non-infected individuals responding to each recombinant protein. Figures (c,d,e,f,g,h,i and j) compare frequencies and RI’s of infected and non-infected individuals’ IgG subclasses to each recombinant protein. Significant differences were indicated by *. (*) $p<0.05$; (**) $p<0.005$; *** $p<0.0005$.
