# Supplementary material for: Recombinant Plasmodium vivax circumsporozoite surface protein allelic variants: antibody recognition by individuals from three communities in the Brazilian Amazon
Source: Sci Rep. 2020 Aug 20;10:14020. doi: 10.1038/s41598-020-70893-3 (PMC7441389; doi:10.1038/s41598-020-70893-3)
Supplement: Supplementary file 2 — Supplementary Information 2. [file 41598_2020_70893_MOESM2_ESM.pdf]

Recombinant *Plasmodium vivax* Circumsporozoite Surface Protein allelic variants: antibody recognition by individuals from three communities in the Brazilian Amazon

Isabela Ferreira Soares[#1], César López-Camacho[#2], Rodrigo Nunes Rodrigues-da-Silva[#3], Ada da Silva Matos[1], Barbara de Oliveira Baptista[4], Paulo Renato Rivas Totino[4], Rodrigo Medeiros de Souza[5], Kate Harrison[2], Alba Marina Gimenez[2], Elisângela Oliveira de Freitas[2], Young Chan Kim[2], Joseli Oliveira-Ferreira[1], Cláudio Tadeu Daniel-Ribeiro[4,6], Arturo Reyes-Sandoval[2], Lilian Rose Pratt-Riccio[4], Josué da Costa Lima-Junior[1]

					VK210									
**Sample**	**Years of residence in endemic area**	**Number of previous malaria episodes**	**Previous Species**	**Current infection**	**IgG OD**	**IgG RI**	**IgG1 OD**	**IgG1 RI**	**IgG2 OD**	**IgG2 RI**	**IgG3 OD**	**IgG3 RI**	**IgG4 OD**	**IgG4 RI**
CZS001	13	4	-	*P. vivax*	0,276	1,326	0,115	0,731	0,223	1,326	0,134	0,693	0,141	0,449
CZS002	3	40	*P. vivax/P. falciparum*	NO	0,307	1,475	0,602	3,807	0,276	1,641	0,158	0,812	0,122	0,388
CZS003	37	2	*P. falciparum*	NO	0,204	0,980	-	-	-	-	-	-	-	-
CZS004	16	20	*P. vivax*	NO	0,142	0,683	-	-	-	-	-	-	-	-
CZS005	28	2	*P. vivax*	*P. vivax*	0,496	2,383	0,374	2,364	1,433	8,530	0,167	0,863	0,243	0,772
CZS006	35	5	*P. vivax/P. falciparum*	NO	0,138	0,663	-	-	-	-	-	-	-	-
CZS007	18	10	*P. vivax/P. falciparum*	NO	0,126	0,607	-	-	-	-	-	-	-	-
CZS008	24	1	*P. vivax*	*P. falciparum*	0,193	0,929	-	-	-	-	-	-	-	-
CZS009	15	15	*P. vivax/P. falciparum*	NO	0,145	0,698	-	-	-	-	-	-	-	-
CZS010	24	20	-	*P. falciparum*	0,107	0,514	-	-	-	-	-	-	-	-
CZS011	16	10	-	*P. falciparum*	0,233	1,119	0,176	1,114	0,110	0,657	0,136	0,703	0,160	0,509
CZS012	60	2	*P. vivax*	NO	0,347	1,668	0,443	2,801	0,199	1,183	0,129	0,665	0,173	0,549
CZS013	68	1	*P. falciparum*	NO	0,273	1,314	0,483	3,057	0,303	1,804	0,134	0,691	0,271	0,859
CZS014	35	4	*P. vivax/P. falciparum*	NO	0,423	2,036	0,257	1,627	0,152	0,904	0,161	0,83	0,134	0,425
CZS015	56	20	*P. vivax/P. falciparum*	NO	0,237	1,139	0,192	1,215	0,243	1,447	0,153	0,791	0,170	0,541
CZS016	42	6	*P. vivax/P. falciparum*	NO	0,439	2,112	0,204	1,291	0,397	2,366	0,199	1,026	0,106	0,338
CZS017	47	100	*P. vivax/P. falciparum*	NO	0,273	1,313	0,355	2,247	0,138	0,820	0,126	0,649	0,159	0,504
CZS018	34	5	*P. vivax/P. falciparum*	NO	0,244	1,171	0,129	0,817	0,316	1,881	0,153	0,788	0,194	0,615
CZS019	60	1	*P. vivax*	NO	0,542	2,608	0,190	1,203	0,313	1,861	0,454	2,34	0,254	0,807
CZS020	22	1	*P. vivax*	*P. vivax*	0,109	0,523	-	-	-	-	-	-	-	-
CZS021	16	4	-	NO	0,122	0,587	-	-	-	-	-	-	-	-
CZS022	46	1	*P. vivax*	NO	0,552	2,655	0,313	1,984	0,671	3,992	0,214	1,105	0,216	0,687
CZS023	36	1	*P. vivax*	NO	0,264	1,27	0,165	1,044	0,340	2,024	0,123	0,636	0,133	0,423
CZS024	29	10	*P. vivax/P. falciparum*	*P. falciparum*	0,190	0,914	-	-	-	-	-	-	-	-
CZS025	57	10	*P. vivax/P. falciparum*	NO	0,372	1,787	0,343	2,174	0,312	1,855	0,174	0,899	0,103	0,328
CZS026	24	2	*P. falciparum*	*P. falciparum*	0,247	1,189	1,993	12,612	0,255	1,516	0,187	0,966	0,122	0,388
CZS027	71	6	*P. vivax/P. falciparum*	*P. falciparum*	0,174	0,838	-	-	-	-	-	-	-	-
CZS028	34	0	-	NO	0,303	1,457	0,723	4,573	0,178	1,061	0,144	0,74	0,107	0,339

					VK210									
**Sample**	**Years of residence in endemic area**	**Number of previous malaria episodes**	**Previous Species**	**Current infection**	**IgG OD**	**IgG RI**	**IgG1 OD**	**IgG1 RI**	**IgG2 OD**	**IgG2 RI**	**IgG3 OD**	**IgG3 RI**	**IgG4 OD**	**IgG4 RI**
CZS029	33	5	*P. falciparum*	NO	0,154	0,742	-	-	-	-	-	-	-	-
CZS030	18	-	-	NO	0,126	0,607	-	-	-	-	-	-	-	-
CZS031	50	20	*P. vivax/P. falciparum*	*P. vivax*	0,391	1,881	2,988	18,91	0,550	3,272	0,153	0,788	0,214	0,679
CZS032	19	20	*P. vivax/P. falciparum*	*P. falciparum*	0,160	0,771	-	-	-	-	-	-	-	-
CZS033	23	2	-	NO	0,312	1,501	0,246	1,56	0,352	2,095	0,199	1,028	0,116	0,368
CZS034	18	4	*P. vivax/P. falciparum*	*P. vivax*	0,291	1,399	0,260	1,646	0,366	2,179	0,210	1,085	0,196	0,622
CZS035	47	-	*P. vivax/P. falciparum*	NO	0,287	1,381	0,104	0,658	0,337	2,003	0,172	0,886	0,132	0,42
CZS036	32	2	*P. falciparum*	NO	0,605	2,909	0,524	3,314	0,445	2,651	0,165	0,848	0,101	0,322
CZS037	62	6	*P. vivax/P. falciparum*	NO	0,303	1,457	0,180	1,139	0,220	1,308	0,137	0,706	0,144	0,458
CZS038	35	2	*P. vivax/P. falciparum*	NO	0,318	1,531	0,123	0,779	0,240	1,430	0,187	0,964	0,171	0,544
CZS039	16	12	*P. vivax/P. falciparum*	NO	0,281	1,35	0,222	1,402	0,241	1,433	0,210	1,085	0,192	0,61
CZS040	40	14	*P. vivax/P. falciparum*	NO	0,344	1,655	0,184	1,162	0,270	1,605	0,244	1,257	0,216	0,687
CZS041	63	-	-	NO	0,519	2,495	0,293	1,855	0,657	3,908	0,179	0,925	0,158	0,501
CZS042	-	15	*P. vivax/P. falciparum*	NO	0,255	1,228	0,172	1,086	0,152	0,904	0,191	0,984	0,173	0,55
CZS043	22	5	*P. vivax*	NO	0,527	2,532	0,195	1,234	0,220	1,311	0,191	0,987	0,162	0,515
CZS044	-	15	*P. vivax/P. falciparum*	NO	0,184	0,884	-	-	-	-	-	-	-	-
CZS045	19	1	*P. vivax*	*P. vivax*	0,322	1,549	0,275	1,741	0,206	1,227	0,142	0,732	0,144	0,457
CZS046	39	10	*P. vivax/P. falciparum*	NO	0,365	1,754	0,562	3,56	0,144	0,859	0,135	0,698	0,135	0,428
CZS047	16	5	*P. vivax*	NO	0,108	0,517	-	-	-	-	-	-	-	-
CZS048	18	2	*P. falciparum*	*P. falciparum*	0,167	0,803	-	-	-	-	-	-	-	-
CZS049	47	10	*P. vivax/P. falciparum*	NO	0,226	1,086	0,171	1,082	0,514	3,058	0,243	1,25	0,179	0,569
CZS050	19	10	*P. vivax/P. falciparum*	*P. falciparum*	0,254	1,223	0,134	0,848	0,313	1,864	0,120	0,621	0,108	0,342
CZS051	27	3	*P. vivax/P. falciparum*	NO	0,313	1,504	0,178	1,124	0,111	0,663	1,050	5,411	0,157	0,498
CZS052	23	5	*P. vivax/P. falciparum*	NO	0,100	0,482	-	-	-	-	-	-	-	-
CZS053	18	10	*P. vivax/P. falciparum*	NO	0,138	0,663	-	-	-	-	-	-	-	-
CZS054	25	1	*P. falciparum*	*P. falciparum*	0,249	1,195	0,728	4,605	0,187	1,112	0,185	0,953	0,163	0,517
CZS055	36	1	*P. falciparum*	NO	0,476	2,287	0,112	0,712	0,543	3,234	0,137	0,706	0,136	0,433
CZS056	31	3	*P. vivax/P. falciparum*	*P. falciparum*	0,125	0,599	-	-	-	-	-	-	-	-

					VK210									
**Sample**	**Years of residence in endemic area**	**Number of previous malaria episodes**	**Previous Species**	**Current infection**	**IgG OD**	**IgG RI**	**IgG1 OD**	**IgG1 RI**	**IgG2 OD**	**IgG2 RI**	**IgG3 OD**	**IgG3 RI**	**IgG4 OD**	**IgG4 RI**
CZS057	18	1	*P. vivax*	NO	0,085	0,409	-	-	-	-	-	-	-	-
CZS058	33	10	*P. vivax/P. falciparum*	NO	0,117	0,564	-	-	-	-	-	-	-	-
CZS059	17	5	*P. vivax/P. falciparum*	*P. vivax*	0,180	0,865	-	-	-	-	-	-	-	-
CZS060	17	1	*P. falciparum*	*P. falciparum*	0,148	0,71	-	-	-	-	-	-	-	-
CZS061	36	2	*P. falciparum*	*P. falciparum*	0,569	2,736	0,249	1,576	0,283	1,685	0,132	0,68	0,138	0,438
CZS062	25	2	*P. vivax*	NO	0,158	0,759	-	-	-	-	-	-	-	-
CZS063	33	1	*P. vivax*	*P. vivax*	0,318	1,531	0,127	0,801	0,252	1,498	0,118	0,608	0,187	0,595
CZS064	39	10	*P. vivax/P. falciparum*	*P. vivax*	0,269	1,294	0,117	0,741	0,368	2,193	0,151	0,778	0,190	0,604
CZS065	30	4	*P. vivax/P. falciparum*	*P. falciparum*	0,534	2,566	0,103	0,655	0,250	1,489	0,162	0,837	0,156	0,496
CZS066	50	20	*P. vivax/P. falciparum*	*P. vivax*	0,259	1,247	0,560	3,545	0,418	2,488	0,172	0,889	0,179	0,568
CZS067	50	10	*P. vivax/P. falciparum*	*P. vivax*	0,246	1,183	0,099	0,627	0,301	1,792	0,171	0,884	0,193	0,614
CZS068	20	8	*P. vivax/P. falciparum*	NO	0,369	1,773	0,195	1,234	0,828	4,928	0,133	0,685	0,250	0,794
CZS069	44	10	*P. vivax/P. falciparum*	NO	0,334	1,605	0,360	2,279	0,288	1,715	0,136	0,703	0,228	0,725
CZS070	12	10	*P. vivax/P. falciparum*	*P. vivax*	0,185	0,888	-	-	-	-	-	-	-	-
CZS071	51	50	*P. vivax/P. falciparum*	NO	0,253	1,218	0,185	1,174	0,372	2,217	0,235	1,211	0,204	0,648
CZS072	23	6	*P. vivax/P. falciparum*	*P. falciparum*	0,379	1,822	0,198	1,25	0,090	0,538	0,142	0,734	0,075	0,239
CZS073	19	23	*P. vivax/P. falciparum*	NO	0,358	1,72	0,298	1,886	0,076	0,452	0,133	0,685	0,067	0,212
CZS074	15	10	*P. vivax/P. falciparum*	NO	0,110	0,529	-	-	-	-	-	-	-	-
CZS075	19	20	*P. vivax/P. falciparum*	*P. vivax*	0,341	1,641	0,535	3,386	0,079	0,470	0,135	0,696	0,100	0,317
CZS076	44	40	*P. vivax/P. falciparum*	NO	0,168	0,809	-	-	-	-	-	-	-	-
CZS077	48	12	*P. vivax/P. falciparum*	NO	0,126	0,605	-	-	-	-	-	-	-	-
CZS078	48	10	*P. vivax/P. falciparum*	NO	0,350	1,682	0,203	1,285	0,082	0,487	0,146	0,755	0,100	0,317
CZS079	48	3	*P. vivax/P. falciparum*	*P. falciparum*	0,128	0,613	-	-	-	-	-	-	-	-
CZS080	21	10	*P. vivax/P. falciparum*	NO	0,567	2,728	0,427	2,703	0,074	0,443	0,126	0,647	0,103	0,327
CZS081	19	1	-	*P. falciparum*	0,220	1,06	0,639	4,042	0,070	0,419	0,147	0,76	0,065	0,205
CZS082	20	2	*P. vivax*	*P. falciparum*	0,502	2,412	0,149	0,943	0,066	0,392	0,980	5,05	0,083	0,265
CZS083	23	3	*P. vivax/P. falciparum*	*P. vivax*	0,094	0,452	-	-	-	-	-	-	-	-
CZS084	22	10	*P. vivax/P. falciparum*	NO	0,157	0,754	-	-	-	-	-	-	-	-

Sample	Years of residence in endemic area	Number of previous malaria episodes	Previous Species	Current infection	VK210									
					IgG OD	IgG RI	IgG1 OD	IgG1 RI	IgG2 OD	IgG2 RI	IgG3 OD	IgG3 RI	IgG4 OD	IgG4 RI
CZS085	25	1	-	*P. falciparum*	0,233	1,122	0,547	3,459	0,064	0,380	0,159	0,822	0,067	0,212
CZS086	25	1	-	NO	0,260	1,25	0,158	1,001	0,074	0,440	0,145	0,747	0,090	0,287
CZS087	20	8	*P. vivax*	NO	0,088	0,421	-	-	-	-	-	-	-	-
CZS088	29	2	*P. vivax*	NO	0,180	0,867	-	-	-	-	-	-	-	-
CZS089	32	1	-	NO	0,091	0,438	-	-	-	-	-	-	-	-
CZS090	23	1	*P. vivax*	*P. vivax*	0,119	0,573	-	-	-	-	-	-	-	-
CZS091	22	1	*P. vivax*	*P. vivax*	0,153	0,736	-	-	-	-	-	-	-	-
CZS092	25	5	*P. vivax*	*P. vivax*	0,100	0,481	-	-	-	-	-	-	-	-
CZS093	39	40	*P. vivax*	*P. vivax*	0,437	2,1	0,445	2,817	0,062	0,372	0,188	0,969	0,052	0,165
CZS094	33	8	*P. vivax/P. falciparum*	NO	0,100	0,482	-	-	-	-	-	-	-	-
CZS095	20	6	*P. vivax/P. falciparum*	*P. vivax*	0,324	1,56	0,179	1,13	0,065	0,386	0,182	0,938	0,056	0,179
CZS096	56	2	*P. vivax*	NO	0,108	0,518	-	-	-	-	-	-	-	-
CZS097	29	15	*P. vivax/P. falciparum*	*P. vivax*	0,175	0,843	-	-	-	-	-	-	-	-
CZS098	50	-	-	NO	0,157	0,756	-	-	-	-	-	-	-	-
CZS099	56	20	*P. vivax/P. falciparum*	*P. vivax*	0,159	0,765	-	-	-	-	-	-	-	-
CZS100	40	20	*P. vivax/P. falciparum*	*P. vivax*	0,094	0,454	-	-	-	-	-	-	-	-
CZS101	18	20	*P. vivax/P. falciparum*	*P. vivax*	0,102	0,488	-	-	-	-	-	-	-	-
CZS102	53	20	*P. vivax/P. falciparum*	*P. vivax*	0,149	0,716	-	-	-	-	-	-	-	-
CZS103	41	10	*P. vivax/P. falciparum*	*P. falciparum*	0,479	2,301	0,626	3,959	0,056	0,336	0,582	2,999	0,048	0,152
CZS104	63	17	*P. vivax/P. falciparum*	*P. falciparum*	0,335	1,609	0,431	2,725	0,056	0,336	0,232	1,196	0,049	0,157
CZS105	21	10	*P. vivax/P. falciparum*	*P. falciparum*	0,210	1,012	0,468	2,959	0,065	0,386	0,141	0,729	0,053	0,168
CZS106	-	-	-	*P. vivax*	0,161	0,773	-	-	-	-	-	-	-	-
CZS107	28	2	*P. vivax/P. falciparum*	*P. falciparum*	0,229	1,101	0,277	1,756	0,066	0,392	0,139	0,716	0,062	0,197
CZS108	-	-	-	*P. vivax*	0,106	0,509	-	-	-	-	-	-	-	-
CZS109	-	-	-	*P. vivax*	0,094	0,45	-	-	-	-	-	-	-	-
CZS110	73	10	*P. vivax/P. falciparum*	*P. falciparum*	0,113	0,544	-	-	-	-	-	-	-	-
CZS111	-	-	-	*P. vivax*	0,112	0,537	-	-	-	-	-	-	-	-
CZS112	36	10	*P. vivax/P. falciparum*	*P. vivax*	0,209	1,005	0,070	0,446	0,055	0,330	0,374	1,93	0,048	0,152

Sample	Years of residence in endemic area	Number of previous malaria episodes	Previous Species	Current infection	VK210									
					IgG OD	IgG RI	IgG1 OD	IgG1 RI	IgG2 OD	IgG2 RI	IgG3 OD	IgG3 RI	IgG4 OD	IgG4 RI
CZS113	13	20	*P. vivax/P. falciparum*	*P. vivax*	0,105	0,505	-	-	-	-	-	-	-	-
CZS114	-	-	-	*P. vivax*	0,191	0,917	-	-	-	-	-	-	-	-
CZS115	19	1	*P. vivax*	NO	0,090	0,435	-	-	-	-	-	-	-	-
CZS116	-	-	-	*P. vivax*	0,099	0,476	-	-	-	-	-	-	-	-
CZS117	15	10	*P. vivax/P. falciparum*	*P. falciparum*	0,253	1,217	0,231	1,462	0,067	0,398	0,146	0,750	0,074	0,235
CZS118	54	10	*P. vivax/P. falciparum*	*P. vivax*	0,652	3,137	0,258	1,633	0,079	0,473	0,147	0,760	0,058	0,184
CZS119	30	15	*P. vivax/P. falciparum*	*P. vivax*	0,095	0,459	-	-	-	-	-	-	-	-
CZS120	49	45	*P. vivax/P. falciparum*	*P. vivax*	0,109	0,524	-	-	-	-	-	-	-	-
CZS121	-	-	-	*P. vivax*	0,090	0,432	-	-	-	-	-	-	-	-
CZS122	-	-	-	*P. vivax*	0,507	2,439	0,386	2,443	0,060	0,357	0,133	0,685	0,054	0,17
CZS123	-	-	-	*P. vivax*	0,148	0,71	-	-	-	-	-	-	-	-
CZS124	-	-	-	*P. vivax*	0,751	3,611	0,201	1,275	0,073	0,437	0,124	0,639	0,065	0,206
ML001	10	10	*P. vivax/P. falciparum*	*P. vivax*	0,415	0,645	-	-	-	-	-	-	-	-
ML002	36	15	*P. vivax/P. falciparum*	*P. vivax*	0,104	1,748	0,385	2,434	0,274	1,632	0,261	1,345	0,158	0,501
ML003	27	20	*P. vivax/P. falciparum*	*P. vivax*	0,172	0,929	-	-	-	-	-	-	-	-
ML004	25	6	*P. vivax*	NO	0,338	2,477	1,337	8,46	1,094	6,512	0,230	1,188	0,198	0,629
ML005	53	20	*P. vivax/P. falciparum*	*P. vivax*	0,145	0,66	-	-	-	-	-	-	-	-
ML006	38	20	*P. vivax/P. falciparum*	NO	0,264	0,675	-	-	-	-	-	-	-	-
ML007	-	8	*P. vivax/P. falciparum*	NO	0,332	0,838	-	-	-	-	-	-	-	-
ML008	74	72	*P. vivax/P. falciparum*	*P. falciparum*	0,114	1,294	0,435	2,753	0,233	1,388	0,281	1,446	0,223	0,707
ML009	18	24	*P. vivax/P. falciparum*	NO	0,160	0,713	-	-	-	-	-	-	-	-
ML010	6	2	*P. vivax*	NO	0,093	1,978	0,331	2,098	0,603	3,590	0,288	1,482	0,265	0,84
ML011	20	-	*P. vivax/P. falciparum*	*P. vivax*	0,129	1,641	1,450	9,178	0,562	3,344	0,229	1,18	0,235	0,747
ML012	14	1	-	*P. vivax*	0,191	2,255	0,445	2,814	0,187	1,112	0,264	1,363	0,208	0,66
ML013	42	4	*P. vivax*	NO	0,177	2,357	0,139	0,88	0,210	1,248	0,220	1,136	0,132	0,419
ML014	56	5	*P. vivax/P. falciparum*	NO	0,265	1,627	0,384	2,431	0,442	2,630	2,837	14,623	0,201	0,639
ML015	32	5	*P. vivax/P. falciparum*	*P. vivax*	0,144	0,812	-	-	-	-	-	-	-	-
ML016	60	15	*P. vivax/P. falciparum*	NO	0,219	2,696	0,533	3,374	0,058	0,344	3,806	19,617	0,187	0,595

					VK210									
**Sample**	**Years of residence in endemic area**	**Number of previous malaria episodes**	**Previous Species**	**Current infection**	**IgG OD**	**IgG RI**	**IgG1 OD**	**IgG1 RI**	**IgG2 OD**	**IgG2 RI**	**IgG3 OD**	**IgG3 RI**	**IgG4 OD**	**IgG4 RI**
ML017	18	5	*P. vivax*	NO	0,114	0,932	-	-	-	-	-	-	-	-
ML018	34	5	*P. vivax*	NO	0,492	0,564	-	-	-	-	-	-	-	-
ML019	23	13	*P. vivax/P. falciparum*	NO	0,168	0,724	-	-	-	-	-	-	-	-
ML020	28	1	*P. vivax*	*P. vivax*	0,095	2,175	0,749	4,741	0,242	1,441	0,212	1,095	0,135	0,43
ML021	35	20	*P. vivax/P. falciparum*	*P. vivax*	0,230	0,891	-	-	-	-	-	-	-	-
ML022	58	8	*P. vivax/P. falciparum*	*P. vivax*	0,367	1,022	0,908	5,747	0,819	4,877	0,184	0,948	0,216	0,687
ML023	37	3	-	*P. vivax*	0,216	0,599	-	-	-	-	-	-	-	-
ML024	55	3	*P. vivax/P. falciparum*	NO	0,643	0,584	-	-	-	-	-	-	-	-
ML025	56	13	*P. vivax/P. falciparum*	*P. falciparum*	0,355	0,447	-	-	-	-	-	-	-	-
ML026	29	-	*P. vivax/P. falciparum*	*P. vivax*	0,265	2,552	0,509	3,222	0,247	1,468	0,191	0,984	0,239	0,758
ML027	32	10	*P. vivax/P. falciparum*	*P. vivax*	0,224	3,453	0,354	2,241	0,602	3,584	0,269	1,389	0,240	0,761
ML028	22	10	*P. vivax/P. falciparum*	NO	0,445	1,44	2,501	15,827	0,179	1,067	0,399	2,059	0,256	0,812
ML029	43	8	*P. vivax/P. falciparum*	NO	0,152	2,32	1,928	12,201	0,636	3,784	0,165	0,853	0,341	1,083
ML030	48	3	*P. vivax/P. falciparum*	NO	0,186	1,034	0,412	2,608	0,112	0,669	0,268	1,381	0,129	0,409
ML031	27	4	*P. vivax/P. falciparum*	*P. falciparum*	0,338	1,226	0,783	4,953	0,223	1,329	0,200	1,031	0,216	0,687
ML032	58	5	-	NO	0,180	0,923	-	-	-	-	-	-	-	-
ML033	30	20	*P. vivax/P. falciparum*	*P. vivax*	0,720	1,475	1,046	6,621	0,295	1,754	0,194	1,001	0,255	0,81
ML034	25	1	*P. vivax*	NO	0,243	1,951	1,059	6,7	0,757	4,506	0,239	1,234	0,326	1,034
ML035	19	10	*P. vivax/P. falciparum*	*P. falciparum*	0,232	1,034	1,073	6,792	0,127	0,758	0,220	1,136	0,137	0,434
ML036	24	10	*P. vivax/P. falciparum*	NO	0,453	1,929	1,356	8,583	0,134	0,800	0,185	0,953	0,293	0,931
ML037	38	20	*P. vivax/P. falciparum*	NO	0,150	1,285	0,186	1,18	0,486	2,892	0,159	0,819	0,122	0,387
ML038	60	4	*P. vivax/P. falciparum*	NO	0,103	2,065	2,373	15,017	0,952	5,665	0,217	1,118	0,229	0,726
ML039	39	20	*P. vivax/P. falciparum*	NO	0,528	0,989	-	-	-	-	-	-	-	-
ML040	42	15	*P. vivax/P. falciparum*	*P. falciparum*	0,397	3,726	0,864	5,466	0,610	3,632	0,286	1,474	0,205	0,652
ML041	41	20	*P. vivax/P. falciparum*	NO	0,753	0,938	-	-	-	-	-	-	-	-
ML042	13	1	*P. vivax*	NO	0,171	1,568	0,409	2,586	0,145	0,865	0,170	0,874	0,227	0,721
ML043	20	5	*P. vivax/P. falciparum*	NO	0,332	2,418	0,416	2,633	0,215	1,281	0,203	1,044	0,164	0,522
ML044	49	10	*P. vivax/P. falciparum*	*P. falciparum*	0,304	2,177	0,320	2,026	0,443	2,636	0,206	1,064	0,198	0,629

					VK210									
Sample	Years of residence in endemic area	Number of previous malaria episodes	Previous Species	Current infection	IgG OD	IgG RI	IgG1 OD	IgG1 RI	IgG2 OD	IgG2 RI	IgG3 OD	IgG3 RI	IgG4 OD	IgG4 RI
ML045	39	3	*P. vivax*	NO	0,994	1,345	0,113	0,715	0,316	1,881	0,175	0,904	0,115	0,366
ML046	45	4	*P. vivax/P. falciparum*	NO	0,353	2,92	0,137	0,864	0,608	3,617	0,154	0,796	0,154	0,49
ML047	17	8	*P. vivax/P. falciparum*	NO	0,247	2,887	0,563	3,564	0,330	1,962	0,185	0,955	0,144	0,458
ML048	88	-	*P. vivax/P. falciparum*	NO	0,341	1,741	0,875	5,538	0,292	1,739	0,176	0,907	0,277	0,878
ML049	14	1	-	NO	0,134	0,397	-	-	-	-	-	-	-	-
ML050	39	80	*P. vivax/P. falciparum*	NO	0,102	2,103	1,770	11,204	0,240	1,427	0,165	0,848	0,127	0,404
ML051	65	10	*P. vivax/P. falciparum*	NO	0,188	0,532	-	-	-	-	-	-	-	-
ML052	29	30	*P. vivax/P. falciparum*	NO	0,273	1,822	0,361	2,285	0,124	0,737	0,213	1,098	0,247	0,783
ML053	36	20	*P. vivax/P. falciparum*	NO	0,542	1,136	1,182	7,482	0,121	0,719	0,153	0,791	0,101	0,322
ML054	50	20	*P. vivax/P. falciparum*	NO	0,246	1,542	0,627	3,969	0,183	1,088	0,170	0,874	0,131	0,415
ML055	21	15	*P. vivax/P. falciparum*	NO	0,306	1,574	0,128	0,81	0,551	3,278	0,235	1,211	0,162	0,515
ML056	15	15	*P. vivax/P. falciparum*	*P. vivax*	0,179	0,698	-	-	-	-	-	-	-	-
ML057	20	20	*P. vivax*	*P. vivax*	0,379	0,768	-	-	-	-	-	-	-	-
ML058	22	6	*P. vivax/P. falciparum*	*P. falciparum*	0,129	2,211	2,181	13,802	0,520	3,094	0,201	1,036	0,191	0,607
ML059	33	5	*P. vivax*	NO	0,334	0,692	-	-	-	-	-	-	-	-
ML060	59	30	*P. vivax/P. falciparum*	NO	0,182	0,753	-	-	-	-	-	-	-	-
ML061	20	10	*P. vivax/P. falciparum*	NO	0,121	0,59	-	-	-	-	-	-	-	-
ML062	49	5	*P. vivax*	NO	0,158	3,765	0,425	2,687	1,158	6,895	0,225	1,162	0,162	0,515
ML063	39	10	*P. vivax/P. falciparum*	NO	0,145	1,218	0,385	2,434	0,347	2,063	0,148	0,763	0,135	0,427
ML064	37	10	*P. vivax/P. falciparum*	NO	0,161	4,163	0,436	2,76	1,013	6,028	0,337	1,739	0,153	0,487
ML065	19	8	*P. vivax/P. falciparum*	NO	0,360	0,777	-	-	-	-	-	-	-	-
ML066	21	6	*P. vivax/P. falciparum*	NO	0,149	3,724	0,122	0,775	0,257	1,528	0,165	0,853	0,113	0,358
ML067	39	6	*P. vivax/P. falciparum*	NO	0,469	1,691	0,276	1,747	0,217	1,290	0,356	1,837	0,135	0,427
ML068	29	3	*P. vivax*	*P. vivax*	0,382	2,141	0,947	5,994	0,495	2,945	0,164	0,845	0,167	0,531
ML069	18	30	*P. vivax/P. falciparum*	NO	0,240	1,988	2,183	13,815	0,375	2,232	0,195	1,005	0,226	0,717
ML070	15	30	*P. vivax/P. falciparum*	*P. vivax/P. falciparum*	0,235	0,802	-	-	-	-	-	-	-	-
ML071	35	20	*P. vivax/P. falciparum*	NO	0,143	0,66	-	-	-	-	-	-	-	-
ML072	16	15	*P. vivax*	*P. vivax/P. falciparum*	0,143	0,578	-	-	-	-	-	-	-	-

					VK210									
Sample	Years of residence in endemic area	Number of previous malaria episodes	Previous Species	Current infection	IgG OD	IgG RI	IgG1 OD	IgG1 RI	IgG2 OD	IgG2 RI	IgG3 OD	IgG3 RI	IgG4 OD	IgG4 RI
ML073	13	5	*P. vivax/P. falciparum*	NO	0,279	0,897	-	-	-	-	-	-	-	-
ML074	13	15	*P. vivax/P. falciparum*	NO	0,225	1,139	0,673	4,257	0,112	0,669	0,249	1,286	0,177	0,561
ML075	31	8	*P. vivax/P. falciparum*	NO	0,621	0,546	-	-	-	-	-	-	-	-
ML076	59	20	*P. vivax/P. falciparum*	NO	0,416	1,241	0,679	4,295	0,142	0,844	0,229	1,18	0,225	0,715
ML077	24	10	*P. vivax*	*P. vivax*	0,380	1,054	1,182	7,482	0,439	2,613	0,420	2,164	0,181	0,574
ML078	38	4	*P. vivax/P. falciparum*	NO	0,435	0,745	-	-	-	-	-	-	-	-
ML079	28	6	*P. vivax/P. falciparum*	*P. vivax*	0,545	3,37	0,819	5,181	0,297	1,765	0,262	1,353	0,179	0,568
ML080	23	15	*P. vivax/P. falciparum*	*P. falciparum*	0,335	1,277	0,952	6,023	0,221	1,317	0,220	1,134	0,192	0,609
ML081	54	4	*P. vivax/P. falciparum*	*P. vivax*	0,372	1,509	0,465	2,94	0,145	0,865	0,117	0,603	0,178	0,564
ML082	54	7	*P. vivax/P. falciparum*	*P. vivax*	0,279	1,013	0,293	1,855	0,270	1,605	0,159	0,822	0,178	0,566
ML083	38	5	*P. vivax*	*P. vivax/P. falciparum*	0,396	1,308	0,836	5,288	0,175	1,043	0,184	0,946	0,175	0,557
ML084	55	20	*P. vivax/P. falciparum*	*P. falciparum*	0,418	1,735	1,002	6,339	0,171	1,016	0,223	1,147	0,154	0,488
ML085	28	2	*P. vivax*	*P. vivax*	0,756	1,202	0,109	0,69	0,207	1,230	0,198	1,023	0,148	0,471
ML086	28	33	*P. vivax/P. falciparum*	*P. vivax*	0,392	1,973	1,669	10,564	0,133	0,794	0,315	1,626	0,197	0,625
ML087	42	12	*P. vivax/P. falciparum*	*P. vivax*	0,570	1,503	0,776	4,912	0,155	0,921	0,200	1,031	0,211	0,671
ML088	-	50	*P. vivax/P. falciparum*	*P. falciparum*	0,134	1,549	0,550	3,481	0,297	1,765	0,220	1,136	0,256	0,812
GJ001	48	40	*P. vivax/P. falciparum*	NO	0,364	1,995	0,724	4,58	0,073	0,437	0,136	0,701	0,056	0,179
GJ002	46	4	*P. vivax/P. falciparum*	NO	0,193	0,498	-	-	-	-	-	-	-	-
GJ003	52	3	*P. vivax/P. falciparum*	NO	0,515	0,827	-	-	-	-	-	-	-	-
GJ004	20	5	*P. vivax/P. falciparum*	NO	0,137	1,627	0,309	1,956	0,075	0,446	0,127	0,654	0,065	0,206
GJ005	20	6	*P. vivax/P. falciparum*	*P. vivax*	0,140	0,695	-	-	-	-	-	-	-	-
GJ006	23	1	-	NO	0,174	1,268	0,343	2,168	0,075	0,446	0,123	0,636	0,057	0,181
GJ007	29	1	*P. vivax*	NO	0,269	1,595	0,413	2,614	0,074	0,443	0,115	0,593	0,057	0,181
GJ008	50	5	*P. vivax/P. falciparum*	*P. falciparum*	0,148	0,548	-	-	-	-	-	-	-	-
GJ009	34	6	*P. vivax/P. falciparum*	NO	0,411	0,771	-	-	-	-	-	-	-	-
GJ010	33	10	*P. vivax/P. falciparum*	*P. falciparum*	0,341	0,447	-	-	-	-	-	-	-	-
GJ011	41	4	*P. vivax*	NO	0,469	0,62	-	-	-	-	-	-	-	-
GJ012	36	20	*P. falciparum*	NO	0,490	0,92	-	-	-	-	-	-	-	-

					VK210									
Sample	Years of residence in endemic area	Number of previous malaria episodes	Previous Species	Current infection	IgG OD	IgG RI	IgG1 OD	IgG1 RI	IgG2 OD	IgG2 RI	IgG3 OD	IgG3 RI	IgG4 OD	IgG4 RI
GJ013	21	2	*P. vivax/P. falciparum*	*P. vivax*	0,338	0,85	-	-	-	-	-	-	-	-
GJ014	55	-	-	NO	0,169	1,276	0,664	4,203	0,085	0,508	0,141	0,729	0,059	0,187
GJ015	21	4	*P. vivax/P. falciparum*	NO	0,561	0,692	-	-	-	-	-	-	-	-
GJ016	25	0	-	NO	0,194	1,054	0,514	3,253	0,083	0,493	0,146	0,755	0,065	0,206
GJ017	22	2	*P. vivax*	NO	0,117	0,546	-	-	-	-	-	-	-	-
GJ018	50	13	*P. vivax/P. falciparum*	NO	0,151	2,364	0,414	2,621	0,084	0,499	0,141	0,729	0,077	0,243
GJ019	13	5	*P. vivax*	*P. vivax*	0,452	0,806	-	-	-	-	-	-	-	-
GJ020	14	1	*P. vivax*	*P. falciparum*	0,185	0,456	-	-	-	-	-	-	-	-
GJ021	43	9	*P. vivax/P. falciparum*	NO	0,213	1,104	0,376	2,38	1,207	7,184	0,328	1,693	0,475	1,509
GJ022	20	1	*P. falciparum*	NO	0,125	1,764	0,244	1,544	0,570	3,394	0,305	1,574	0,329	1,046
GJ023	18	0	-	NO	0,121	1,04	0,733	4,64	0,118	0,701	0,209	1,077	0,402	1,276
GJ024	17	4	*P. vivax/P. falciparum*	NO	0,093	3,09	0,528	3,342	0,666	3,965	0,262	1,35	0,516	1,638
GJ025	25	5	*P. vivax*	NO	0,531	1,706	0,450	2,845	0,423	2,517	0,387	1,994	0,230	0,729
GJ026	56	5	*P. vivax/P. falciparum*	*P. falciparum*	0,718	1,276	0,499	3,159	0,421	2,506	0,329	1,698	0,265	0,842
GJ027	38	4	*P. vivax*	NO	0,300	1,078	0,078	0,491	0,133	0,794	0,330	1,703	0,247	0,783
GJ028	54	3	*P. vivax/P. falciparum*	NO	0,483	2,141	0,150	0,947	0,356	2,122	0,330	1,703	0,512	1,624
GJ029	31	7	*P. vivax/P. falciparum*	*P. falciparum*	0,215	0,733	-	-	-	-	-	-	-	-
GJ030	-	2	-	*P. vivax*	0,255	0,892	-	-	-	-	-	-	-	-
GJ031	16	4	*P. vivax/P. falciparum*	NO	0,192	1,624	0,532	3,367	0,220	1,311	0,222	1,144	0,400	1,27
GJ032	34	4	*P. vivax/P. falciparum*	NO	0,307	0,867	-	-	-	-	-	-	-	-
GJ033	29	15	*P. vivax/P. falciparum*	NO	0,406	3,462	1,265	8,007	1,341	7,980	0,164	0,843	0,281	0,893
GJ034	22	4	*P. vivax*	NO	0,215	1,169	0,166	1,048	0,127	0,758	0,158	0,814	0,211	0,671
GJ035	26	15	*P. vivax*	NO	0,401	1,114	0,146	0,921	0,141	0,841	0,215	1,108	0,305	0,967
GJ036	23	4	*P. vivax/P. falciparum*	NO	0,267	2,179	0,445	2,814	0,319	1,896	0,178	0,915	0,238	0,755
GJ037	35	10	*P. vivax/P. falciparum*	NO	0,430	0,721	-	-	-	-	-	-	-	-
GJ038	20	10	*P. vivax/P. falciparum*	NO	0,206	0,497	-	-	-	-	-	-	-	-
GJ039	65	1	-	NO	0,775	2,537	0,090	0,567	0,234	1,394	0,220	1,134	0,212	0,672
GJ040	50	0	-	NO	0,195	1,911	0,845	5,349	0,231	1,376	0,196	1,01	0,160	0,507

					VK210									
**Sample**	**Years of residence in endemic area**	**Number of previous malaria episodes**	**Previous Species**	**Current infection**	**IgG OD**	**IgG RI**	**IgG1 OD**	**IgG1 RI**	**IgG2 OD**	**IgG2 RI**	**IgG3 OD**	**IgG3 RI**	**IgG4 OD**	**IgG4 RI**
GJ041	21	1	*P. vivax*	NO	0,326	3,619	0,220	1,393	0,345	2,051	0,133	0,683	0,179	0,568
GJ042	25	1	*P. vivax*	NO	0,503	0,824	-	-	-	-	-	-	-	-
GJ043	29	9	*P. vivax/P. falciparum*	NO	0,453	1,595	0,252	1,595	0,219	1,305	0,208	1,072	0,227	0,721
GJ044	20	1	-	*P. vivax*	0,280	1,463	1,397	8,843	0,199	1,186	0,287	1,479	0,256	0,812
GJ045	50	20	*P. vivax/P. falciparum*	NO	0,607	4,781	0,341	2,158	0,465	2,770	0,199	1,028	0,259	0,823
GJ046	36	0	-	NO	0,600	1,697	0,237	1,497	0,362	2,155	0,159	0,819	0,233	0,739
GJ047	56	-	*P. vivax/P. falciparum*	NO	0,362	1,186	0,601	3,804	0,132	0,785	0,142	0,732	0,187	0,593
GJ048	39	14	*P. vivax/P. falciparum*	NO	0,083	1,641	0,511	3,234	0,233	1,388	0,146	0,752	0,195	0,618
GJ049	21	0	-	NO	0,437	0,645	-	-	-	-	-	-	-	-
GJ050	38	3	*P. vivax/P. falciparum*	NO	0,111	0,491	-	-	-	-	-	-	-	-
GJ051	32	15	*P. vivax*	NO	0,379	0,902	-	-	-	-	-	-	-	-
GJ052	56	1	*P. vivax*	NO	0,236	1,311	0,751	4,75	0,138	0,823	0,165	0,853	0,165	0,525
GJ053	34	1	*P. vivax*	NO	0,321	2,605	0,107	0,68	1,318	7,843	0,169	0,871	0,743	2,359
GJ054	30	15	*P. vivax/P. falciparum*	*P. vivax*	0,327	1,182	0,819	5,181	0,189	1,126	0,228	1,175	0,220	0,699
GJ055	15	3	*P. falciparum*	*P. vivax/P. falciparum*	0,145	1,469	0,078	0,491	0,110	0,654	0,431	2,221	0,707	2,245
GJ056	46	5	*P. vivax/P. falciparum*	*P. vivax*	0,160	0,862	-	-	-	-	-	-	-	-
GJ057	26	40	*P. vivax/P. falciparum*	NO	0,460	1,823	0,733	4,637	0,130	0,776	0,132	0,678	0,167	0,531
GJ058	57	10	*P. vivax/P. falciparum*	NO	0,144	0,622	-	-	-	-	-	-	-	-
GJ059	28	20	*P. vivax/P. falciparum*	NO	0,157	1,606	1,278	8,086	0,122	0,728	0,139	0,719	0,191	0,607
GJ060	23	50	*P. vivax/P. falciparum*	NO	0,123	0,877	-	-	-	-	-	-	-	-
GJ061	22	8	*P. vivax/P. falciparum*	NO	0,783	0,584	-	-	-	-	-	-	-	-
GJ062	20	40	*P. vivax/P. falciparum*	*P. vivax*	0,253	0,762	-	-	-	-	-	-	-	-
GJ063	62	10	*P. vivax/P. falciparum*	NO	0,866	0,698	-	-	-	-	-	-	-	-
GJ064	27	15	*P. vivax/P. falciparum*	*P. falciparum*	0,162	0,774	-	-	-	-	-	-	-	-
GJ065	23	20	*P. vivax/P. falciparum*	NO	0,775	1,731	0,582	3,681	0,208	1,236	0,215	1,108	0,171	0,542
GJ066	48	5	*P. vivax/P. falciparum*	NO	0,352	0,718	-	-	-	-	-	-	-	-
GJ067	50	20	*P. vivax/P. falciparum*	NO	0,445	2,255	0,292	1,845	0,239	1,421	2,567	13,234	0,139	0,441
GJ068	53	7	*P. falciparum*	NO	0,414	1,837	0,365	2,31	0,840	4,999	0,197	1,015	0,267	0,847

					VK210									
Sample	Years of residence in endemic area	Number of previous malaria episodes	Previous Species	Current infection	IgG OD	IgG RI	IgG1 OD	IgG1 RI	IgG2 OD	IgG2 RI	IgG3 OD	IgG3 RI	IgG4 OD	IgG4 RI
GJ069	55	25	*P. vivax/P. falciparum*	NO	0,167	1,156	0,631	3,994	0,143	0,853	0,190	0,977	0,164	0,522
GJ070	55	30	*P. vivax/P. falciparum*	NO	0,137	1,13	0,490	3,102	0,216	1,287	0,219	1,131	0,183	0,582
GJ071	58	0	-	NO	0,120	0,689	-	-	-	-	-	-	-	-
GJ072	39	2	*P. vivax/P. falciparum*	NO	0,187	0,686	-	-	-	-	-	-	-	-
GJ073	73	4	-	NO	0,237	1,341	1,033	6,535	0,229	1,361	0,149	0,77	0,171	0,542
GJ074	65	10	*P. vivax*	NO	0,114	1,081	1,220	7,719	0,185	1,102	0,174	0,897	0,172	0,547
GJ075	24	3	*P. vivax*	NO	0,258	2,984	0,141	0,892	0,224	1,332	0,128	0,662	0,157	0,499
GJ076	67	3	-	NO	0,219	2,001	0,492	3,114	0,148	0,883	0,119	0,611	0,205	0,65
GJ077	79	3	*P. falciparum*	NO	0,155	1,828	0,509	3,222	0,129	0,770	0,147	0,758	0,137	0,436
GJ078	83	1	-	NO	0,701	2,093	0,470	2,972	0,233	1,388	0,133	0,683	0,185	0,588
GJ079	20	1	*P. falciparum*	NO	0,266	2,621	0,497	3,146	0,528	3,145	0,205	1,056	0,143	0,453
GJ080	16	5	*P. vivax/P. falciparum*	*P. vivax*	0,314	1,611	0,119	0,75	0,438	2,610	0,158	0,814	0,282	0,894
GJ081	31	10	*P. vivax/P. falciparum*	NO	0,211	1,789	0,094	0,598	0,295	1,757	0,134	0,691	0,240	0,763
GJ082	29	10	*P. vivax/P. falciparum*	NO	0,272	1,339	1,153	7,298	0,255	1,519	0,138	0,711	0,180	0,572
GJ083	44	10	*P. vivax/P. falciparum*	NO	0,361	1,902	0,109	0,693	0,378	2,250	0,158	0,817	0,213	0,675
GJ084	73	10	*P. vivax/P. falciparum*	NO	0,250	2,012	0,959	6,07	0,649	3,861	0,161	0,832	0,287	0,91
GJ085	37	8	*P. vivax/P. falciparum*	NO	0,410	3,636	3,378	21,379	0,302	1,795	0,258	1,332	0,186	0,591
GJ086	30	32	*P. vivax/P. falciparum*	NO	0,313	1,883	0,219	1,389	0,288	1,712	0,244	1,26	0,171	0,544
GJ087	57	25	*P. vivax/P. falciparum*	*P. vivax*	0,322	2,738	0,550	3,478	0,317	1,884	0,217	1,121	0,219	0,696
Control 001	0	0	NO	NO	0,087	0,418	-	-	-	-	-	-	-	-
Control 002	0	0	NO	NO	0,084	0,404	-	-	-	-	-	-	-	-
Control 003	0	0	NO	NO	0,123	0,591	-	-	-	-	-	-	-	-
Control 004	0	0	NO	NO	0,126	0,606	0,093	0,589	0,118	0,702	0,163	0,840	0,092	0,292
Control 005	0	0	NO	NO	0,12	0,577	-	-	-	-	-	-	-	-
Control 006	0	0	NO	NO	0,112	0,538	-	-	-	-	-	-	-	-
Control 007	0	0	NO	NO	0,094	0,452	-	-	-	-	-	-	-	-
Control 008	0	0	NO	NO	0,131	0,630	0,098	0,620	0,093	0,551	0,146	0,753	0,201	0,638
Control 009	0	0	NO	NO	0,137	0,659	0,118	0,747	0,081	0,482	0,146	0,750	0,198	0,629

Sample	Years of residence in endemic area	Number of previous malaria episodes	Previous Species	Current infection	VK210									
					IgG OD	IgG RI	IgG1 OD	IgG1 RI	IgG2 OD	IgG2 RI	IgG3 OD	IgG3 RI	IgG4 OD	IgG4 RI
Control 010	0	0	NO	NO	0,095	0,457	-	-	-	-	-	-	-	-
Control 011	0	0	NO	NO	0,079	0,380	-	-	-	-	-	-	-	-
Control 012	0	0	NO	NO	0,083	0,399	-	-	-	-	-	-	-	-
Control 013	0	0	NO	NO	0,076	0,365	-	-	-	-	-	-	-	-
Control 014	0	0	NO	NO	0,086	0,413	-	-	-	-	-	-	-	-
Control 015	0	0	NO	NO	0,143	0,688	0,115	0,728	0,131	0,780	0,153	0,786	0,092	0,292
Control 016	0	0	NO	NO	0,076	0,365	-	-	-	-	-	-	-	-
Control 017	0	0	NO	NO	0,071	0,341	-	-	-	-	-	-	-	-
Control 018	0	0	NO	NO	0,086	0,413	-	-	-	-	-	-	-	-
Control 019	0	0	NO	NO	0,089	0,428	-	-	-	-	-	-	-	-
Control 020	0	0	NO	NO	0,066	0,317	-	-	-	-	-	-	-	-
Control 021	0	0	NO	NO	0,146	0,702	0,125	0,791	0,115	0,685	0,166	0,853	0,102	0,324
Control 022	0	0	NO	NO	0,078	0,375	-	-	-	-	-	-	-	-
Control 023	0	0	NO	NO	0,146	0,702	0,124	0,785	0,115	0,682	0,131	0,675	0,086	0,273
Control 024	0	0	NO	NO	0,122	0,587	-	-	-	-	-	-	-	-
Control 025	0	0	NO	NO	0,125	0,601	-	-	-	-	-	-	-	-
Control 026	0	0	NO	NO	0,123	0,591	-	-	-	-	-	-	-	-
Control 027	0	0	NO	NO	0,122	0,587	-	-	-	-	-	-	-	-
Control 028	0	0	NO	NO	0,089	0,428	-	-	-	-	-	-	-	-
Control 029	0	0	NO	NO	0,112	0,538	-	-	-	-	-	-	-	-
Control 030	0	0	NO	NO	0,123	0,591	-	-	-	-	-	-	-	-
Control 031	0	0	NO	NO	0,066	0,317	-	-	-	-	-	-	-	-
Control 032	0	0	NO	NO	0,080	0,385	-	-	-	-	-	-	-	-
Control 033	0	0	NO	NO	0,094	0,452	-	-	-	-	-	-	-	-
Control 034	0	0	NO	NO	0,111	0,534	-	-	-	-	-	-	-	-
Control 035	0	0	NO	NO	0,098	0,471	-	-	-	-	-	-	-	-
Control 036	0	0	NO	NO	0,183	0,880	0,119	0,753	0,138	0,818	0,134	0,691	0,187	0,594
Control 037	0	0	NO	NO	0,125	0,601	-	-	-	-	-	-	-	-

Sample	Years of residence in endemic area	Number of previous malaria episodes	Previous Species	Current infection	VK210									
					IgG OD	IgG RI	IgG1 OD	IgG1 RI	IgG2 OD	IgG2 RI	IgG3 OD	IgG3 RI	IgG4 OD	IgG4 RI
Control 038	0	0	NO	NO	0,121	0,582	-	-	-	-	-	-	-	-
Control 039	0	0	NO	NO	0,115	0,553	-	-	-	-	-	-	-	-
Control 040	0	0	NO	NO	0,097	0,466	-	-	-	-	-	-	-	-
Control 041	0	0	NO	NO	0,149	0,716	0,121	0,763	0,088	0,524	0,175	0,902	0,189	0,600
Control 042	0	0	NO	NO	0,079	0,380	-	-	-	-	-	-	-	-
Control 043	0	0	NO	NO	0,119	0,572	-	-	-	-	-	-	-	-
Control 044	0	0	NO	NO	0,113	0,543	-	-	-	-	-	-	-	-
Control 045	0	0	NO	NO	0,098	0,471	-	-	-	-	-	-	-	-
Control 046	0	0	NO	NO	0,153	0,736	0,128	0,810	0,115	0,685	0,140	0,719	0,087	0,276
Control 047	0	0	NO	NO	0,123	0,591	-	-	-	-	-	-	-	-
Control 048	0	0	NO	NO	0,084	0,404	-	-	-	-	-	-	-	-
Control 049	0	0	NO	NO	0,116	0,558	-	-	-	-	-	-	-	-
Control 050	0	0	NO	NO	0,184	0,885	0,137	0,867	0,125	0,744	0,159	0,817	0,216	0,686
Control 051	0	0	NO	NO	0,120	0,577	-	-	-	-	-	-	-	-
Control 052	0	0	NO	NO	0,122	0,587	-	-	-	-	-	-	-	-
Control 053	0	0	NO	NO	0,118	0,567	-	-	-	-	-	-	-	-

	VK247										*P. vivax* -like							
**Sample**	**IgG OD**	**IgG RI**	**IgG1 OD**	**IgG1 RI**	**IgG2 OD**	**IgG2 RI**	**IgG3 OD**	**IgG3 RI**	**IgG4 OD**	**IgG4 RI**	**IgG OD**	**IgG RI**	**IgG1 OD**	**IgG1 RI**	**IgG2 OD**	**IgG2 RI**	**IgG3 OD**	**IgG3 RI**
CZS001	0,402	1,548	0,239	1,019	0,175	0,518	0,192	0,668	0,266	0,606	0,411	1,532	0,046	0,773	0,047	0,884	0,060	0,542
CZS002	0,448	1,723	0,606	2,586	0,262	0,777	0,228	0,793	0,197	0,449	0,470	1,754	0,045	0,748	0,044	0,837	0,229	2,078
CZS003	0,293	1,125	0,325	1,386	0,178	0,528	0,191	0,664	0,131	0,299	0,092	0,342	-	-	-	-	-	-
CZS004	0,233	0,895	-	-	-	-	-	-	-	-	0,192	0,718	-	-	-	-	-	-
CZS005	0,781	3,005	2,006	8,566	1,328	3,941	0,242	0,841	0,287	0,653	0,124	0,463	-	-	-	-	-	-
CZS006	0,204	0,783	-	-	-	-	-	-	-	-	0,184	0,687	-	-	-	-	-	-
CZS007	0,212	0,814	-	-	-	-	-	-	-	-	0,176	0,658	-	-	-	-	-	-
CZS008	0,279	1,073	0,779	3,329	0,171	0,507	0,197	0,685	0,436	0,992	0,234	0,874	-	-	-	-	-	-
CZS009	0,220	0,846	-	-	-	-	-	-	-	-	0,182	0,68	-	-	-	-	-	-
CZS010	0,146	0,563	-	-	-	-	-	-	-	-	0,157	0,587	-	-	-	-	-	-
CZS011	0,150	0,577	-	-	-	-	-	-	-	-	0,161	0,599	-	-	-	-	-	-
CZS012	0,400	1,538	0,412	1,760	0,167	0,495	0,194	0,673	0,269	0,613	0,458	1,710	0,075	1,247	0,047	0,894	0,058	0,524
CZS013	0,446	1,716	0,478	2,042	0,208	0,617	0,206	0,715	0,438	0,998	0,329	1,226	0,066	1,098	0,055	1,042	0,081	0,734
CZS014	0,482	1,852	0,316	1,348	0,709	2,103	0,213	0,739	0,199	0,454	0,661	2,465	0,059	0,989	0,052	0,980	1,424	12,948
CZS015	0,344	1,324	0,491	2,097	0,291	0,863	0,241	0,838	0,352	0,802	0,339	1,264	0,049	0,823	0,052	0,980	0,121	1,098
CZS016	0,658	2,53	0,401	1,713	0,332	0,985	0,174	0,605	0,148	0,338	0,095	0,356	-	-	-	-	-	-
CZS017	0,397	1,526	0,388	1,659	0,120	0,357	0,156	0,541	0,289	0,658	0,095	0,354	-	-	-	-	-	-
CZS018	0,166	0,637	-	-	-	-	-	-	-	-	0,167	0,623	-	-	-	-	-	-
CZS019	0,420	1,614	0,600	2,563	0,231	0,684	0,177	0,614	0,433	0,987	0,348	1,298	0,250	4,166	0,053	0,999	0,066	0,597
CZS020	0,164	0,632	-	-	-	-	-	-	-	-	0,151	0,565	-	-	-	-	-	-
CZS021	0,204	0,786	-	-	-	-	-	-	-	-	0,171	0,637	-	-	-	-	-	-
CZS022	0,748	2,876	0,436	1,864	0,531	1,575	0,266	0,923	0,363	0,826	0,154	0,576	-	-	-	-	-	-
CZS023	0,239	0,919	-	-	-	-	-	-	-	-	0,354	1,32	0,063	1,048	0,043	0,818	0,194	1,768
CZS024	0,232	0,891	-	-	-	-	-	-	-	-	0,223	0,833	-	-	-	-	-	-
CZS025	0,374	1,44	0,273	1,164	0,249	0,740	0,161	0,560	0,125	0,284	0,125	0,468	-	-	-	-	-	-
CZS026	0,254	0,975	-	-	-	-	-	-	-	-	0,239	0,893	-	-	-	-	-	-
CZS027	0,198	0,762	-	-	-	-	-	-	-	-	0,777	2,899	0,060	1,006	0,049	0,923	2,825	25,686
CZS028	0,377	1,45	0,296	1,264	0,142	0,420	0,146	0,506	0,114	0,259	0,096	0,359	-	-	-	-	-	-

	VK247										*P. vivax* -like							
**Sample**	**IgG OD**	**IgG RI**	**IgG1 OD**	**IgG1 RI**	**IgG2 OD**	**IgG2 RI**	**IgG3 OD**	**IgG3 RI**	**IgG4 OD**	**IgG4 RI**	**IgG OD**	**IgG RI**	**IgG1 OD**	**IgG1 RI**	**IgG2 OD**	**IgG2 RI**	**IgG3 OD**	**IgG3 RI**
CZS029	0,192	0,737	-	-	-	-	-	-	-	-	0,148	0,554	-	-	-	-	-	-
CZS030	0,193	0,741	-	-	-	-	-	-	-	-	0,161	0,602	-	-	-	-	-	-
CZS031	0,430	1,653	0,248	1,061	0,256	0,761	0,170	0,589	0,256	0,582	0,492	1,835	0,060	1,006	0,050	0,951	0,038	0,345
CZS032	0,215	0,828	-	-	-	-	-	-	-	-	0,223	0,833	-	-	-	-	-	-
CZS033	0,531	2,041	0,336	1,437	0,187	0,555	0,198	0,688	0,130	0,295	0,251	0,937	-	-	-	-	-	-
CZS034	0,387	1,489	0,549	2,343	0,217	0,644	0,260	0,904	0,314	0,716	0,420	1,567	0,066	1,098	0,045	0,846	0,073	0,661
CZS035	0,116	0,445	-	-	-	-	-	-	-	-	0,164	0,612	-	-	-	-	-	-
CZS036	0,100	0,385	-	-	-	-	-	-	-	-	0,487	1,819	0,048	0,807	0,053	1,008	0,061	0,551
CZS037	0,415	1,597	0,565	2,413	0,161	0,477	0,155	0,537	0,223	0,508	0,151	0,562	-	-	-	-	-	-
CZS038	0,263	1,013	0,307	1,313	0,209	0,621	0,167	0,581	0,191	0,436	0,264	0,986	-	-	-	-	-	-
CZS039	0,253	0,974	-	-	-	-	-	-	-	-	0,305	1,137	0,053	0,881	0,049	0,932	0,075	0,679
CZS040	0,533	2,049	0,283	1,209	0,224	0,664	0,232	0,807	0,239	0,545	0,413	1,541	0,048	0,807	0,049	0,932	0,068	0,615
CZS041	0,407	1,567	0,712	3,041	0,472	1,400	0,160	0,556	0,130	0,297	0,142	0,53	-	-	-	-	-	-
CZS042	0,255	0,982	-	-	-	-	-	-	-	-	0,294	1,096	0,061	1,014	0,045	0,856	0,086	0,779
CZS043	0,313	1,205	0,248	1,061	0,176	0,522	0,205	0,711	0,142	0,323	0,111	0,415	-	-	-	-	-	-
CZS044	0,168	0,647	-	-	-	-	-	-	-	-	0,175	0,653	-	-	-	-	-	-
CZS045	0,283	1,087	0,293	1,251	0,137	0,408	0,151	0,525	0,119	0,270	0,200	0,745	-	-	-	-	-	-
CZS046	0,434	1,67	0,857	3,660	0,117	0,347	0,144	0,501	0,157	0,358	0,103	0,385	-	-	-	-	-	-
CZS047	0,145	0,556	-	-	-	-	-	-	-	-	0,146	0,546	-	-	-	-	-	-
CZS048	0,493	1,896	0,310	1,322	0,167	0,497	0,155	0,539	0,180	0,411	0,176	0,658	-	-	-	-	-	-
CZS049	0,365	1,405	0,766	3,272	0,611	1,814	0,286	0,994	0,187	0,426	0,181	0,674	-	-	-	-	-	-
CZS050	0,323	1,241	0,387	1,653	0,322	0,955	0,129	0,449	0,107	0,244	0,195	0,728	-	-	-	-	-	-
CZS051	0,193	0,744	-	-	-	-	-	-	-	-	0,179	0,668	-	-	-	-	-	-
CZS052	0,144	0,552	-	-	-	-	-	-	-	-	0,139	0,518	-	-	-	-	-	-
CZS053	0,173	0,667	-	-	-	-	-	-	-	-	0,186	0,693	-	-	-	-	-	-
CZS054	0,228	0,877	-	-	-	-	-	-	-	-	0,257	0,958	-	-	-	-	-	-
CZS055	0,671	2,582	0,200	0,854	0,653	1,937	0,154	0,534	0,121	0,276	0,595	2,222	0,049	0,815	0,054	1,027	0,074	0,670
CZS056	0,164	0,629	-	-	-	-	-	-	-	-	0,163	0,609	-	-	-	-	-	-

	VK247										*P. vivax* -like							
**Sample**	**IgG OD**	**IgG RI**	**IgG1 OD**	**IgG1 RI**	**IgG2 OD**	**IgG2 RI**	**IgG3 OD**	**IgG3 RI**	**IgG4 OD**	**IgG4 RI**	**IgG OD**	**IgG RI**	**IgG1 OD**	**IgG1 RI**	**IgG2 OD**	**IgG2 RI**	**IgG3 OD**	**IgG3 RI**
CZS057	0,111	0,426	-	-	-	-	-	-	-	-	0,127	0,474	-	-	-	-	-	-
CZS058	0,177	0,681	-	-	-	-	-	-	-	-	0,155	0,577	-	-	-	-	-	-
CZS059	0,153	0,589	-	-	-	-	-	-	-	-	0,213	0,796	-	-	-	-	-	-
CZS060	0,199	0,765	-	-	-	-	-	-	-	-	0,183	0,683	-	-	-	-	-	-
CZS061	0,911	3,502	0,332	1,420	0,306	0,908	0,159	0,553	0,142	0,323	0,978	3,649	0,050	0,840	0,051	0,961	0,059	0,538
CZS062	0,240	0,923	-	-	-	-	-	-	-	-	0,262	0,977	-	-	-	-	-	-
CZS063	0,193	0,744	-	-	-	-	-	-	-	-	0,161	0,6	-	-	-	-	-	-
CZS064	0,373	1,436	0,262	1,119	0,511	1,517	0,240	0,835	0,292	0,666	0,154	0,573	-	-	-	-	-	-
CZS065	0,212	0,815	-	-	-	-	-	-	-	-	0,196	0,733	-	-	-	-	-	-
CZS066	0,411	1,58	0,556	2,373	0,474	1,407	0,806	2,799	0,249	0,566	0,361	1,348	0,056	0,940	0,047	0,884	0,201	1,828
CZS067	0,423	1,625	0,182	0,777	0,358	1,063	0,798	2,771	0,347	0,791	0,360	1,342	0,052	0,865	0,090	1,693	0,091	0,825
CZS068	0,531	2,044	0,306	1,307	0,940	2,788	0,156	0,542	0,249	0,567	0,205	0,766	-	-	-	-	-	-
CZS069	0,272	1,046	0,352	1,503	0,323	0,958	0,185	0,643	0,333	0,758	0,392	1,463	0,072	1,197	0,052	0,989	0,122	1,107
CZS070	0,244	0,937	-	-	-	-	-	-	-	-	0,240	0,896	-	-	-	-	-	-
CZS071	0,407	1,566	0,262	1,119	0,511	1,517	0,240	0,835	0,292	0,666	0,401	1,495	0,053	0,890	0,051	0,970	0,341	3,104
CZS072	0,522	2,009	0,440	1,879	0,783	2,322	0,238	0,826	0,215	0,490	0,504	1,882	0,054	0,898	0,051	0,970	0,191	1,732
CZS073	0,468	1,8	1,155	4,933	0,213	0,633	0,243	0,843	0,206	0,469	0,471	1,757	0,064	1,073	0,049	0,932	0,295	2,684
CZS074	0,378	1,455	0,292	1,245	0,488	1,449	0,206	0,715	0,215	0,489	0,339	1,264	0,048	0,798	0,049	0,932	0,132	1,203
CZS075	0,178	0,686	-	-	-	-	-	-	-	-	0,455	1,698	0,069	1,156	0,051	0,970	0,274	2,493
CZS076	0,242	0,93	-	-	-	-	-	-	-	-	0,238	0,889	-	-	-	-	-	-
CZS077	0,174	0,669	-	-	-	-	-	-	-	-	0,509	1,901	0,048	0,807	0,049	0,923	0,167	1,518
CZS078	0,102	0,392	-	-	-	-	-	-	-	-	0,436	1,626	0,047	0,782	0,045	0,856	0,111	1,012
CZS079	0,154	0,591	-	-	-	-	-	-	-	-	0,141	0,527	-	-	-	-	-	-
CZS080	0,135	0,519	-	-	-	-	-	-	-	-	0,362	1,349	0,055	0,915	0,046	0,875	0,151	1,376
CZS081	0,186	0,716	-	-	-	-	-	-	-	-	0,223	0,833	-	-	-	-	-	-
CZS082	0,090	0,348	-	-	-	-	-	-	-	-	0,213	0,796	-	-	-	-	-	-
CZS083	0,098	0,377	-	-	-	-	-	-	-	-	0,090	0,337	-	-	-	-	-	-
CZS084	0,070	0,271	-	-	-	-	-	-	-	-	0,207	0,774	-	-	-	-	-	-

	VK247										*P. vivax* -like							
Sample	IgG OD	IgG RI	IgG1 OD	IgG1 RI	IgG2 OD	IgG2 RI	IgG3 OD	IgG3 RI	IgG4 OD	IgG4 RI	IgG OD	IgG RI	IgG1 OD	IgG1 RI	IgG2 OD	IgG2 RI	IgG3 OD	IgG3 RI
CZS085	0,194	0,746	-	-	-	-	-	-	-	-	0,235	0,875	-	-	-	-	-	-
CZS086	0,104	0,400	-	-	-	-	-	-	-	-	0,433	1,617	0,054	0,898	0,048	0,913	0,421	3,824
CZS087	0,114	0,437	-	-	-	-	-	-	-	-	0,120	0,449	-	-	-	-	-	-
CZS088	0,156	0,601	-	-	-	-	-	-	-	-	0,146	0,543	-	-	-	-	-	-
CZS089	0,140	0,538	-	-	-	-	-	-	-	-	0,137	0,512	-	-	-	-	-	-
CZS090	0,214	0,824	-	-	-	-	-	-	-	-	0,465	1,735	0,069	1,147	0,053	0,999	0,245	2,224
CZS091	0,224	0,86	-	-	-	-	-	-	-	-	0,220	0,821	-	-	-	-	-	-
CZS092	0,143	0,551	-	-	-	-	-	-	-	-	0,548	2,045	0,044	0,740	0,053	1,008	0,158	1,436
CZS093	0,156	0,601	-	-	-	-	-	-	-	-	0,616	2,297	0,047	0,782	0,045	0,846	0,178	1,622
CZS094	0,100	0,385	-	-	-	-	-	-	-	-	0,658	2,456	0,047	0,790	0,047	0,894	0,134	1,221
CZS095	0,128	0,493	-	-	-	-	-	-	-	-	0,484	1,807	0,049	0,823	0,046	0,875	0,173	1,577
CZS096	0,164	0,631	-	-	-	-	-	-	-	-	0,223	0,833	-	-	-	-	-	-
CZS097	0,192	0,737	-	-	-	-	-	-	-	-	0,248	0,925	-	-	-	-	-	-
CZS098	0,222	0,853	-	-	-	-	-	-	-	-	0,265	0,989	-	-	-	-	-	-
CZS099	0,196	0,755	-	-	-	-	-	-	-	-	0,180	0,671	-	-	-	-	-	-
CZS100	0,065	0,251	-	-	-	-	-	-	-	-	0,123	0,459	-	-	-	-	-	-
CZS101	0,194	0,748	-	-	-	-	-	-	-	-	0,349	1,303	0,048	0,798	0,047	0,884	0,203	1,841
CZS102	0,185	0,713	-	-	-	-	-	-	-	-	0,196	0,73	-	-	-	-	-	-
CZS103	0,554	2,132	0,308	1,316	0,448	1,330	0,197	0,685	0,104	0,237	0,605	2,256	0,053	0,881	0,051	0,970	0,278	2,525
CZS104	0,490	1,884	0,387	1,653	0,245	0,727	0,215	0,748	0,228	0,520	0,524	1,954	0,047	0,790	0,047	0,884	0,107	0,971
CZS105	0,282	1,085	0,421	1,796	0,188	0,559	0,187	0,650	0,188	0,429	0,292	1,09	0,065	1,089	0,048	0,913	0,275	2,502
CZS106	0,158	0,606	-	-	-	-	-	-	-	-	0,163	0,609	-	-	-	-	-	-
CZS107	0,423	1,628	0,326	1,392	0,116	0,344	0,147	0,509	0,088	0,200	0,321	1,198	0,059	0,981	0,055	1,046	0,165	1,499
CZS108	0,192	0,738	-	-	-	-	-	-	-	-	0,190	0,71	-	-	-	-	-	-
CZS109	0,155	0,597	-	-	-	-	-	-	-	-	0,424	1,581	0,045	0,748	0,056	1,065	0,203	1,841
CZS110	0,467	1,796	0,210	0,895	0,339	1,005	0,183	0,635	0,104	0,236	0,634	2,365	0,056	0,940	0,052	0,980	0,639	5,811
CZS111	0,278	1,069	0,798	3,406	0,127	0,378	0,218	0,758	0,095	0,216	0,166	0,621	-	-	-	-	-	-
CZS112	0,287	1,102	0,242	1,034	0,145	0,430	0,578	2,006	0,116	0,264	0,410	1,528	0,055	0,923	0,053	1,008	0,202	1,837

	VK247										*P. vivax* -like							
**Sample**	**IgG OD**	**IgG RI**	**IgG1 OD**	**IgG1 RI**	**IgG2 OD**	**IgG2 RI**	**IgG3 OD**	**IgG3 RI**	**IgG4 OD**	**IgG4 RI**	**IgG OD**	**IgG RI**	**IgG1 OD**	**IgG1 RI**	**IgG2 OD**	**IgG2 RI**	**IgG3 OD**	**IgG3 RI**
CZS113	0,088	0,337	-	-	-	-	-	-	-	-	0,117	0,438	-	-	-	-	-	-
CZS114	0,276	1,062	0,260	1,111	0,102	0,304	0,206	0,716	0,103	0,235	0,278	1,036	0,199	3,317	0,049	0,923	0,027	0,243
CZS115	0,298	1,148	0,283	1,209	0,146	0,434	0,138	0,478	0,177	0,404	0,520	1,941	0,070	1,172	0,050	0,951	0,065	0,592
CZS116	0,147	0,566	-	-	-	-	-	-	-	-	0,143	0,534	-	-	-	-	-	-
CZS117	0,234	0,899	-	-	-	-	-	-	-	-	0,303	1,129	0,072	1,206	0,051	0,961	0,225	2,046
CZS118	1,087	4,18	0,880	3,757	0,343	1,019	0,189	0,657	0,105	0,240	1,050	3,917	0,055	0,915	0,051	0,961	0,215	1,955
CZS119	0,703	2,705	0,285	1,217	0,117	0,348	0,151	0,523	0,123	0,279	0,126	0,47	-	-	-	-	-	-
CZS120	0,225	0,864	-	-	-	-	-	-	-	-	1,106	4,128	0,050	0,840	0,060	1,132	0,181	1,645
CZS121	0,138	0,532	-	-	-	-	-	-	-	-	0,467	1,744	0,045	0,757	0,049	0,923	0,128	1,167
CZS122	0,788	3,029	0,301	1,286	0,127	0,377	0,180	0,624	0,203	0,462	0,190	0,709	-	-	-	-	-	-
CZS123	0,219	0,843	-	-	-	-	-	-	-	-	0,265	0,987	-	-	-	-	-	-
CZS124	1,013	3,897	0,271	1,158	0,382	1,134	0,149	0,518	0,134	0,306	1,246	4,65	0,067	1,122	0,061	1,151	0,083	0,757
ML001	0,185	0,713	-	-	-	-	-	-	-	-	0,271	1,011	0,088	1,463	0,048	0,904	0,209	1,900
ML002	0,214	0,822	-	-	-	-	-	-	-	-	0,319	1,192	0,056	0,931	0,045	0,846	0,139	1,267
ML003	0,209	0,802	-	-	-	-	-	-	-	-	0,446	1,664	0,049	0,815	0,048	0,904	0,131	1,189
ML004	0,583	2,243	0,199	0,851	0,311	0,924	0,195	0,676	0,476	1,083	0,574	2,14	0,055	0,923	0,048	0,913	0,127	1,158
ML005	0,190	0,730	-	-	-	-	-	-	-	-	0,183	0,683	-	-	-	-	-	-
ML006	0,224	0,863	-	-	-	-	-	-	-	-	0,233	0,871	-	-	-	-	-	-
ML007	0,235	0,905	-	-	-	-	-	-	-	-	0,221	0,824	-	-	-	-	-	-
ML008	0,371	1,426	0,191	0,816	0,324	0,961	0,523	1,817	0,580	1,321	0,399	1,49	0,056	0,940	0,050	0,951	0,145	1,317
ML009	0,212	0,814	-	-	-	-	-	-	-	-	0,233	0,871	-	-	-	-	-	-
ML010	0,081	0,313	-	-	-	-	-	-	-	-	0,474	1,768	0,052	0,873	0,046	0,875	0,153	1,390
ML011	0,493	1,897	0,236	1,009	0,374	1,109	0,204	0,708	0,328	0,748	0,448	1,67	0,086	1,430	0,053	0,999	0,131	1,194
ML012	0,327	1,258	0,199	0,851	0,405	1,201	0,213	0,740	0,321	0,732	0,558	2,081	0,057	0,948	0,049	0,923	0,180	1,632
ML013	0,202	0,777	-	-	-	-	-	-	-	-	0,611	2,281	0,046	0,765	0,047	0,884	0,120	1,094
ML014	0,222	0,853	-	-	-	-	-	-	-	-	0,304	1,136	0,053	0,881	0,051	0,970	0,511	4,644
ML015	0,226	0,870	-	-	-	-	-	-	-	-	0,228	0,852	-	-	-	-	-	-
ML016	0,202	0,776	-	-	-	-	-	-	-	-	0,212	0,79	-	-	-	-	-	-

	VK247										*P. vivax* -like							
**Sample**	**IgG OD**	**IgG RI**	**IgG1 OD**	**IgG1 RI**	**IgG2 OD**	**IgG2 RI**	**IgG3 OD**	**IgG3 RI**	**IgG4 OD**	**IgG4 RI**	**IgG OD**	**IgG RI**	**IgG1 OD**	**IgG1 RI**	**IgG2 OD**	**IgG2 RI**	**IgG3 OD**	**IgG3 RI**
ML017	0,210	0,806	-	-	-	-	-	-	-	-	0,289	1,08	0,049	0,823	0,048	0,904	0,139	1,267
ML018	0,188	0,723	-	-	-	-	-	-	-	-	0,163	0,609	-	-	-	-	-	-
ML019	0,245	0,944	-	-	-	-	-	-	-	-	0,213	0,793	-	-	-	-	-	-
ML020	0,095	0,364	-	-	-	-	-	-	-	-	0,422	1,574	0,051	0,848	0,048	0,904	0,157	1,431
ML021	0,229	0,881	-	-	-	-	-	-	-	-	0,201	0,749	-	-	-	-	-	-
ML022	0,195	0,751	-	-	-	-	-	-	-	-	0,297	1,108	0,067	1,114	0,061	1,151	0,132	1,199
ML023	0,203	0,781	-	-	-	-	-	-	-	-	0,165	0,615	-	-	-	-	-	-
ML024	0,178	0,685	-	-	-	-	-	-	-	-	0,154	0,574	-	-	-	-	-	-
ML025	0,121	0,465	-	-	-	-	-	-	-	-	0,136	0,506	-	-	-	-	-	-
ML026	0,141	0,542	-	-	-	-	-	-	-	-	0,489	1,825	0,058	0,964	0,051	0,970	0,181	1,645
ML027	0,172	0,663	-	-	-	-	-	-	-	-	0,793	2,959	0,048	0,798	0,047	0,884	0,135	1,226
ML028	0,126	0,483	-	-	-	-	-	-	-	-	0,422	1,576	0,054	0,898	0,047	0,894	0,136	1,240
ML029	0,463	1,780	0,224	0,956	0,349	1,035	0,200	0,695	0,671	1,528	0,501	1,869	0,082	1,372	0,051	0,970	0,149	1,358
ML030	0,162	0,624	-	-	-	-	-	-	-	-	0,164	0,613	-	-	-	-	-	-
ML031	0,205	0,788	-	-	-	-	-	-	-	-	0,503	1,877	0,056	0,931	0,049	0,923	0,143	1,299
ML032	0,123	0,472	-	-	-	-	-	-	-	-	0,210	0,783	-	-	-	-	-	-
ML033	0,472	1,817	0,226	0,965	0,355	1,053	0,200	0,695	0,335	0,764	0,444	1,657	0,058	0,964	0,047	0,894	0,135	1,226
ML034	0,479	1,844	0,189	0,807	0,293	0,869	0,198	0,689	0,818	1,863	0,583	2,174	0,053	0,881	0,047	0,894	0,145	1,322
ML035	0,203	0,779	-	-	-	-	-	-	-	-	0,221	0,824	-	-	-	-	-	-
ML036	0,327	1,259	0,189	0,807	0,286	0,850	0,316	1,097	0,385	0,876	0,329	1,227	0,056	0,931	0,047	0,884	0,421	3,828
ML037	0,469	1,803	0,185	0,789	0,336	0,998	0,631	2,193	0,328	0,748	1,298	4,844	0,060	1,006	0,050	0,951	3,618	32,891
ML038	0,265	1,020	0,193	0,824	0,324	0,961	0,310	1,077	0,335	0,764	0,225	0,840	-	-	-	-	-	-
ML039	0,237	0,912	-	-	-	-	-	-	-	-	0,368	1,374	0,061	1,014	0,050	0,942	0,108	0,984
ML040	0,167	0,644	-	-	-	-	-	-	-	-	0,783	2,923	0,054	0,906	0,051	0,970	0,262	2,384
ML041	0,262	1,006	0,193	0,824	0,286	0,850	0,295	1,026	0,307	0,700	1,880	7,016	0,071	1,189	0,047	0,894	3,775	34,317
ML042	0,089	0,341	-	-	-	-	-	-	-	-	0,655	2,445	0,048	0,798	0,046	0,865	0,149	1,358
ML043	0,205	0,788	-	-	-	-	-	-	-	-	0,515	1,921	0,054	0,898	0,047	0,884	0,154	1,404
ML044	0,110	0,424	-	-	-	-	-	-	-	-	0,446	1,666	0,066	1,098	0,049	0,932	0,362	3,295

	VK247										*P. vivax* -like							
**Sample**	**IgG OD**	**IgG RI**	**IgG1 OD**	**IgG1 RI**	**IgG2 OD**	**IgG2 RI**	**IgG3 OD**	**IgG3 RI**	**IgG4 OD**	**IgG4 RI**	**IgG OD**	**IgG RI**	**IgG1 OD**	**IgG1 RI**	**IgG2 OD**	**IgG2 RI**	**IgG3 OD**	**IgG3 RI**
ML045	0,101	0,388	-	-	-	-	-	-	-	-	0,359	1,34	0,052	0,865	0,047	0,884	0,151	1,372
ML046	0,529	2,036	0,185	0,789	0,380	1,127	0,184	0,638	0,328	0,748	0,132	0,492	-	-	-	-	-	-
ML047	0,473	1,818	0,195	0,833	0,411	1,220	0,184	0,638	0,328	0,748	0,640	2,387	0,055	0,923	0,050	0,951	0,113	1,025
ML048	0,554	2,132	0,218	0,930	0,305	0,906	1,990	6,911	0,335	0,764	0,431	1,61	0,069	1,147	0,050	0,951	0,104	0,948
ML049	0,122	0,468	-	-	-	-	-	-	-	-	0,118	0,44	-	-	-	-	-	-
ML050	0,445	1,712	0,201	0,859	0,723	2,144	0,211	0,733	0,335	0,764	0,577	2,153	0,078	1,297	0,049	0,923	0,260	2,361
ML051	0,148	0,570	-	-	-	-	-	-	-	-	0,142	0,531	-	-	-	-	-	-
ML052	0,196	0,752	-	-	-	-	-	-	-	-	0,534	1,994	0,052	0,865	0,048	0,904	0,255	2,315
ML053	0,387	1,489	0,238	1,017	0,311	0,924	0,185	0,644	0,412	0,939	0,361	1,348	0,079	1,322	0,051	0,970	0,166	1,513
ML054	0,217	0,836	-	-	-	-	-	-	-	-	0,397	1,48	0,066	1,098	0,048	0,913	0,093	0,848
ML055	0,173	0,665	-	-	-	-	-	-	-	-	0,188	0,700	-	-	-	-	-	-
ML056	0,159	0,612	-	-	-	-	-	-	-	-	0,146	0,543	-	-	-	-	-	-
ML057	0,220	0,846	-	-	-	-	-	-	-	-	0,246	0,918	-	-	-	-	-	-
ML058	0,171	0,656	-	-	-	-	-	-	-	-	0,147	0,550	-	-	-	-	-	-
ML059	0,182	0,699	-	-	-	-	-	-	-	-	0,198	0,737	-	-	-	-	-	-
ML060	0,182	0,701	-	-	-	-	-	-	-	-	0,164	0,613	-	-	-	-	-	-
ML061	0,154	0,591	-	-	-	-	-	-	-	-	0,153	0,571	-	-	-	-	-	-
ML062	1,198	4,606	0,205	0,877	0,318	0,943	0,198	0,689	0,517	1,178	1,002	3,739	0,051	0,848	0,054	1,018	0,073	0,661
ML063	0,220	0,847	-	-	-	-	-	-	-	-	0,236	0,882	-	-	-	-	-	-
ML064	0,619	2,382	0,287	1,224	0,713	2,116	0,290	1,008	0,353	0,805	0,606	2,26	0,067	1,114	0,052	0,980	0,082	0,747
ML065	0,259	0,996	-	-	-	-	-	-	-	-	0,218	0,815	-	-	-	-	-	-
ML066	0,111	0,426	-	-	-	-	-	-	-	-	0,148	0,554	-	-	-	-	-	-
ML067	0,083	0,318	-	-	-	-	-	-	-	-	0,133	0,495	-	-	-	-	-	-
ML068	0,625	2,404	0,385	1,644	0,310	0,921	0,200	0,695	0,241	0,550	0,142	0,531	-	-	-	-	-	-
ML069	0,387	1,488	1,076	4,594	0,243	0,722	0,203	0,704	0,469	1,068	0,156	0,583	-	-	-	-	-	-
ML070	0,099	0,381	-	-	-	-	-	-	-	-	0,140	0,523	-	-	-	-	-	-
ML071	0,128	0,493	-	-	-	-	-	-	-	-	0,124	0,462	-	-	-	-	-	-
ML072	0,125	0,482	-	-	-	-	-	-	-	-	0,143	0,534	-	-	-	-	-	-

	VK247										*P. vivax* -like							
**Sample**	**IgG OD**	**IgG RI**	**IgG1 OD**	**IgG1 RI**	**IgG2 OD**	**IgG2 RI**	**IgG3 OD**	**IgG3 RI**	**IgG4 OD**	**IgG4 RI**	**IgG OD**	**IgG RI**	**IgG1 OD**	**IgG1 RI**	**IgG2 OD**	**IgG2 RI**	**IgG3 OD**	**IgG3 RI**
ML073	0,252	0,968	-	-	-	-	-	-	-	-	0,222	0,827	-	-	-	-	-	-
ML074	0,340	1,307	0,359	1,533	0,147	0,437	0,195	0,678	0,201	0,458	0,137	0,511	-	-	-	-	-	-
ML075	0,161	0,619	-	-	-	-	-	-	-	-	0,304	1,133	0,047	0,790	0,054	1,018	0,073	0,661
ML076	0,288	1,108	0,382	1,632	0,167	0,495	0,231	0,801	0,754	1,718	0,288	1,074	0,065	1,089	0,046	0,875	0,081	0,734
ML077	0,472	1,817	1,066	4,551	0,399	1,185	0,296	1,027	0,406	0,924	0,181	0,677	-	-	-	-	-	-
ML078	0,164	0,629	-	-	-	-	-	-	-	-	0,186	0,693	-	-	-	-	-	-
ML079	1,091	4,197	0,356	1,521	0,210	0,624	0,172	0,596	0,220	0,500	0,205	0,764	-	-	-	-	-	-
ML080	0,231	0,887	-	-	-	-	-	-	-	-	0,232	0,867	-	-	-	-	-	-
ML081	0,349	1,344	0,214	0,912	0,184	0,546	0,162	0,562	0,471	1,073	0,223	0,833	-	-	-	-	-	-
ML082	0,300	1,153	0,180	0,767	0,292	0,867	0,178	0,619	0,472	1,075	0,214	0,798	-	-	-	-	-	-
ML083	0,248	0,954	-	-	-	-	-	-	-	-	0,230	0,858	-	-	-	-	-	-
ML084	0,540	2,076	0,547	2,336	0,215	0,638	0,240	0,833	0,390	0,889	0,149	0,556	-	-	-	-	-	-
ML085	0,172	0,662	-	-	-	-	-	-	-	-	0,141	0,525	-	-	-	-	-	-
ML086	0,669	2,572	1,134	4,841	0,174	0,515	0,236	0,819	0,760	1,732	0,656	2,447	0,055	0,923	0,047	0,894	0,077	0,702
ML087	0,233	0,897	-	-	-	-	-	-	-	-	0,333	1,242	0,060	1,006	0,047	0,894	0,055	0,501
ML088	0,434	1,670	0,316	1,348	0,321	0,953	0,221	0,768	0,738	1,681	0,437	1,629	0,056	0,931	0,051	0,961	0,061	0,551
GJ001	0,783	3,012	0,282	1,204	0,266	0,789	0,151	0,525	0,097	0,220	0,565	2,107	0,051	0,856	0,055	1,037	0,198	1,800
GJ002	0,342	1,317	0,255	1,091	0,173	0,512	0,144	0,501	0,119	0,270	0,413	1,54	0,055	0,915	0,050	0,942	0,134	1,217
GJ003	0,282	1,083	0,249	1,064	0,175	0,520	0,167	0,579	0,138	0,315	0,269	1,005	0,052	0,873	0,049	0,932	0,139	1,267
GJ004	0,462	1,775	0,262	1,119	0,356	1,057	0,189	0,657	0,108	0,246	0,104	0,387	-	-	-	-	-	-
GJ005	0,205	0,790	-	-	-	-	-	-	-	-	0,225	0,84	-	-	-	-	-	-
GJ006	0,299	1,150	0,257	1,098	0,540	1,603	0,175	0,608	0,204	0,465	0,274	1,024	0,059	0,981	0,049	0,923	0,193	1,750
GJ007	0,447	1,719	0,365	1,557	0,450	1,335	0,186	0,647	0,108	0,246	0,380	1,417	0,061	1,014	0,051	0,970	0,156	1,422
GJ008	0,111	0,426	-	-	-	-	-	-	-	-	0,388	1,449	0,059	0,989	0,056	1,056	0,242	2,201
GJ009	0,171	0,657	-	-	-	-	-	-	-	-	0,217	0,808	-	-	-	-	-	-
GJ010	0,137	0,528	-	-	-	-	-	-	-	-	0,137	0,512	-	-	-	-	-	-
GJ011	0,137	0,527	-	-	-	-	-	-	-	-	0,217	0,810	-	-	-	-	-	-
GJ012	0,170	0,653	-	-	-	-	-	-	-	-	0,155	0,580	-	-	-	-	-	-

	VK247										*P. vivax* -like							
Sample	IgG OD	IgG RI	IgG1 OD	IgG1 RI	IgG2 OD	IgG2 RI	IgG3 OD	IgG3 RI	IgG4 OD	IgG4 RI	IgG OD	IgG RI	IgG1 OD	IgG1 RI	IgG2 OD	IgG2 RI	IgG3 OD	IgG3 RI
GJ013	0,221	0,849	-	-	-	-	-	-	-	-	0,209	0,780	-	-	-	-	-	-
GJ014	0,291	1,118	0,417	1,783	0,316	0,939	0,214	0,744	0,112	0,256	0,263	0,983	-	-	-	-	-	-
GJ015	0,231	0,888	-	-	-	-	-	-	-	-	0,304	1,136	0,047	0,790	0,042	0,789	0,441	4,006
GJ016	0,211	0,810	-	-	-	-	-	-	-	-	0,303	1,13	0,052	0,873	0,043	0,808	0,221	2,010
GJ017	0,184	0,706	-	-	-	-	-	-	-	-	0,183	0,683	-	-	-	-	-	-
GJ018	0,697	2,679	0,272	1,160	0,147	0,437	0,187	0,648	0,223	0,508	0,743	2,773	0,058	0,964	0,044	0,837	0,408	3,705
GJ019	0,229	0,880	-	-	-	-	-	-	-	-	0,243	0,907	-	-	-	-	-	-
GJ020	0,145	0,559	-	-	-	-	-	-	-	-	0,152	0,566	-	-	-	-	-	-
GJ021	0,116	0,445	-	-	-	-	-	-	-	-	0,321	1,198	0,050	0,840	0,042	0,789	0,364	3,309
GJ022	0,065	0,250	-	-	-	-	-	-	-	-	0,530	1,979	0,101	1,680	0,042	0,789	0,255	2,315
GJ023	0,094	0,360	-	-	-	-	-	-	-	-	0,337	1,258	0,070	1,172	0,046	0,865	0,230	2,092
GJ024	0,992	3,816	0,226	0,965	0,330	0,980	0,332	1,154	1,070	2,436	0,831	3,102	0,117	1,954	0,056	1,065	0,269	2,443
GJ025	0,468	1,800	0,199	0,851	0,361	1,072	0,242	0,842	0,350	0,796	0,463	1,726	0,053	0,881	0,054	1,027	0,312	2,839
GJ026	0,361	1,387	0,183	0,781	0,349	1,035	0,250	0,867	0,357	0,812	0,359	1,339	0,052	0,873	0,055	1,037	0,363	3,304
GJ027	0,323	1,241	0,187	0,798	0,305	0,906	0,279	0,969	0,510	1,162	0,327	1,22	0,045	0,748	0,046	0,875	0,260	2,365
GJ028	0,756	2,907	0,649	2,771	0,330	0,980	0,235	0,816	0,440	1,003	0,676	2,522	0,072	1,206	0,065	1,227	0,282	2,561
GJ029	0,267	1,027	0,224	0,956	0,318	0,943	0,242	0,842	0,552	1,258	0,225	0,840	-	-	-	-	-	-
GJ030	0,193	0,744	-	-	-	-	-	-	-	-	0,195	0,729	-	-	-	-	-	-
GJ031	0,459	1,765	0,191	0,816	0,299	0,887	0,233	0,810	0,545	1,242	0,539	2,010	0,054	0,898	0,047	0,894	0,059	0,536
GJ032	0,218	0,838	-	-	-	-	-	-	-	-	0,251	0,936	-	-	-	-	-	-
GJ033	0,743	2,859	0,326	1,394	0,361	1,072	0,240	0,835	0,447	1,019	1,224	4,568	0,074	1,239	0,078	1,465	0,215	1,955
GJ034	0,168	0,646	-	-	-	-	-	-	-	-	0,310	1,157	0,053	0,881	0,044	0,837	0,199	1,805
GJ035	0,254	0,978	-	-	-	-	-	-	-	-	0,603	2,249	0,047	0,782	0,050	0,942	2,498	22,710
GJ036	0,194	0,748	-	-	-	-	-	-	-	-	0,784	2,924	0,056	0,940	0,068	1,274	0,194	1,768
GJ037	0,214	0,822	-	-	-	-	-	-	-	-	0,184	0,687	-	-	-	-	-	-
GJ038	0,113	0,433	-	-	-	-	-	-	-	-	0,110	0,409	-	-	-	-	-	-
GJ039	0,136	0,523	-	-	-	-	-	-	-	-	0,528	1,969	0,047	0,790	0,053	1,002	0,058	0,531
GJ040	0,101	0,390	-	-	-	-	-	-	-	-	0,136	0,506	-	-	-	-	-	-

	VK247										*P. vivax*-like							
**Sample**	**IgG OD**	**IgG RI**	**IgG1 OD**	**IgG1 RI**	**IgG2 OD**	**IgG2 RI**	**IgG3 OD**	**IgG3 RI**	**IgG4 OD**	**IgG4 RI**	**IgG OD**	**IgG RI**	**IgG1 OD**	**IgG1 RI**	**IgG2 OD**	**IgG2 RI**	**IgG3 OD**	**IgG3 RI**
GJ041	0,156	0,601	-	-	-	-	-	-	-	-	0,225	0,838	-	-	-	-	-	-
GJ042	0,241	0,926	-	-	-	-	-	-	-	-	0,247	0,921	-	-	-	-	-	-
GJ043	0,110	0,424	-	-	-	-	-	-	-	-	0,494	1,844	0,054	0,898	0,045	0,846	0,119	1,085
GJ044	0,416	1,600	0,224	0,956	0,299	0,887	0,310	1,077	0,391	0,891	0,405	1,51	0,078	1,305	0,046	0,875	0,089	0,807
GJ045	0,177	0,682	-	-	-	-	-	-	-	-	1,609	6,005	0,058	0,964	0,050	0,942	0,194	1,764
GJ046	0,101	0,388	-	-	-	-	-	-	-	-	0,482	1,798	0,055	0,915	0,048	0,904	0,184	1,677
GJ047	0,084	0,322	-	-	-	-	-	-	-	-	0,172	0,643	-	-	-	-	-	-
GJ048	0,557	2,142	0,191	0,816	0,299	0,887	0,240	0,835	0,538	1,226	0,454	1,695	0,058	0,964	0,048	0,913	0,212	1,923
GJ049	0,199	0,765	-	-	-	-	-	-	-	-	0,222	0,827	-	-	-	-	-	-
GJ050	0,118	0,454	-	-	-	-	-	-	-	-	0,129	0,481	-	-	-	-	-	-
GJ051	0,228	0,877	-	-	-	-	-	-	-	-	0,217	0,808	-	-	-	-	-	-
GJ052	0,060	0,229	-	-	-	-	-	-	-	-	0,372	1,389	0,057	0,956	0,047	0,884	0,304	2,762
GJ053	0,227	0,873	-	-	-	-	-	-	-	-	0,734	2,737	0,048	0,798	0,045	0,856	0,238	2,160
GJ054	0,251	0,965	-	-	-	-	-	-	-	-	0,265	0,987	-	-	-	-	-	-
GJ055	0,235	0,903	-	-	-	-	-	-	-	-	0,248	0,926	-	-	-	-	-	-
GJ056	0,230	0,885	-	-	-	-	-	-	-	-	0,265	0,989	-	-	-	-	-	-
GJ057	0,227	0,874	-	-	-	-	-	-	-	-	0,226	0,843	-	-	-	-	-	-
GJ058	0,175	0,674	-	-	-	-	-	-	-	-	0,165	0,615	-	-	-	-	-	-
GJ059	0,233	0,896	-	-	-	-	-	-	-	-	0,388	1,446	0,069	1,156	0,046	0,875	0,198	1,800
GJ060	0,173	0,665	-	-	-	-	-	-	-	-	0,241	0,898	-	-	-	-	-	-
GJ061	0,181	0,695	-	-	-	-	-	-	-	-	0,191	0,712	-	-	-	-	-	-
GJ062	0,204	0,783	-	-	-	-	-	-	-	-	0,226	0,843	-	-	-	-	-	-
GJ063	0,205	0,790	-	-	-	-	-	-	-	-	0,219	0,818	-	-	-	-	-	-
GJ064	0,199	0,765	-	-	-	-	-	-	-	-	0,181	0,674	-	-	-	-	-	-
GJ065	0,232	0,894	-	-	-	-	-	-	-	-	0,494	1,843	0,054	0,906	0,052	0,980	0,187	1,700
GJ066	0,238	0,916	-	-	-	-	-	-	-	-	0,182	0,680	-	-	-	-	-	-
GJ067	0,108	0,417	-	-	-	-	-	-	-	-	0,401	1,495	0,057	0,956	0,051	0,970	0,530	4,817
GJ068	0,140	0,538	-	-	-	-	-	-	-	-	0,557	2,078	0,074	1,239	0,051	0,961	0,123	1,121

	VK247										***P. vivax*-like**							
**Sample**	**IgG OD**	**IgG RI**	**IgG1 OD**	**IgG1 RI**	**IgG2 OD**	**IgG2 RI**	**IgG3 OD**	**IgG3 RI**	**IgG4 OD**	**IgG4 RI**	**IgG OD**	**IgG RI**	**IgG1 OD**	**IgG1 RI**	**IgG2 OD**	**IgG2 RI**	**IgG3 OD**	**IgG3 RI**
GJ069	0,213	0,820	-	-	-	-	-	-	-	-	0,413	1,542	0,051	0,848	0,054	1,018	0,113	1,030
GJ070	0,238	0,916	-	-	-	-	-	-	-	-	0,319	1,189	0,058	0,973	0,050	0,951	0,227	2,060
GJ071	0,204	0,786	-	-	-	-	-	-	-	-	0,207	0,774	-	-	-	-	-	-
GJ072	0,176	0,678	-	-	-	-	-	-	-	-	0,157	0,587	-	-	-	-	-	-
GJ073	0,093	0,356	-	-	-	-	-	-	-	-	0,394	1,47	0,194	3,226	0,047	0,894	0,177	1,613
GJ074	0,289	1,111	0,255	1,087	0,361	1,072	0,239	0,829	0,321	0,732	0,272	1,014	0,067	1,122	0,069	1,293	0,159	1,449
GJ075	0,101	0,388	-	-	-	-	-	-	-	-	0,499	1,862	0,053	0,881	0,055	1,037	0,169	1,536
GJ076	0,219	0,843	-	-	-	-	-	-	-	-	0,699	2,608	0,063	1,048	0,051	0,961	0,106	0,966
GJ077	0,631	2,425	0,199	0,851	0,349	1,035	0,217	0,752	0,335	0,764	0,664	2,477	0,068	1,131	0,088	1,655	0,135	1,226
GJ078	0,092	0,354	-	-	-	-	-	-	-	-	0,187	0,698	-	-	-	-	-	-
GJ079	0,090	0,347	-	-	-	-	-	-	-	-	0,822	3,068	0,055	0,915	0,049	0,923	0,117	1,066
GJ080	0,168	0,646	-	-	-	-	-	-	-	-	0,515	1,923	0,054	0,898	0,052	0,980	0,189	1,718
GJ081	0,242	0,930	-	-	-	-	-	-	-	-	0,483	1,802	0,060	1,005	0,049	0,932	0,079	0,718
GJ082	0,222	0,853	-	-	-	-	-	-	-	-	0,455	1,696	0,060	0,998	0,047	0,894	0,139	1,267
GJ083	0,152	0,584	-	-	-	-	-	-	-	-	0,570	2,127	0,047	0,782	0,049	0,932	0,080	0,725
GJ084	0,184	0,707	-	-	-	-	-	-	-	-	0,731	2,727	0,059	0,989	0,050	0,942	3,595	32,681
GJ085	0,157	0,604	-	-	-	-	-	-	-	-	1,084	4,043	0,095	1,580	0,049	0,923	0,210	1,910
GJ086	0,214	0,822	-	-	-	-	-	-	-	-	0,497	1,853	0,050	0,831	0,051	0,961	0,129	1,176
GJ087	0,871	3,351	0,230	0,982	0,330	0,980	0,360	1,250	0,342	0,780	0,957	3,572	0,102	1,704	0,054	1,027	0,449	4,079
Control 001	0,115	0,442	-	-	-	-	-	-	-	-	0,140	0,522	-	-	-	-	-	-
Control 002	0,108	0,415	-	-	-	-	-	-	-	-	0,130	0,485	-	-	-	-	-	-
Control 003	0,134	0,515	-	-	-	-	-	-	-	-	0,138	0,515	-	-	-	-	-	-
Control 004	0,146	0,562	0,099	0,423	0,089	0,264	0,154	0,535	0,086	0,196	0,147	0,549	0,049	0,820	0,048	0,906	0,079	0,714
Control 005	0,132	0,508	-	-	-	-	-	-	-	-	0,127	0,474	-	-	-	-	-	-
Control 006	0,149	0,573	0,100	0,427	0,221	0,656	0,180	0,625	0,276	0,629	0,090	0,336	-	-	-	-	-	-
Control 007	0,077	0,296	-	-	-	-	-	-	-	-	0,230	0,858	0,056	0,933	0,049	0,925	0,075	0,682
Control 008	0,073	0,281	-	-	-	-	-	-	-	-	0,082	0,306	-	-	-	-	-	-
Control 009	0,124	0,477	-	-	-	-	-	-	-	-	0,130	0,485	-	-	-	-	-	-

	VK247										*P. vivax*-like							
**Sample**	**IgG OD**	**IgG RI**	**IgG1 OD**	**IgG1 RI**	**IgG2 OD**	**IgG2 RI**	**IgG3 OD**	**IgG3 RI**	**IgG4 OD**	**IgG4 RI**	**IgG OD**	**IgG RI**	**IgG1 OD**	**IgG1 RI**	**IgG2 OD**	**IgG2 RI**	**IgG3 OD**	**IgG3 RI**
Control 010	0,105	0,404	-	-	-	-	-	-	-	-	0,185	0,690	0,049	0,817	0,048	0,906	0,068	0,618
Control 011	0,101	0,388	-	-	-	-	-	-	-	-	0,123	0,459	-	-	-	-	-	-
Control 012	0,099	0,381	-	-	-	-	-	-	-	-	0,104	0,388	-	-	-	-	-	-
Control 013	0,081	0,312	-	-	-	-	-	-	-	-	0,101	0,377	-	-	-	-	-	-
Control 014	0,082	0,315	-	-	-	-	-	-	-	-	0,089	0,332	-	-	-	-	-	-
Control 015	0,139	0,535	0,147	0,626	0,102	0,303	0,111	0,384	0,092	0,210	0,087	0,325	-	-	-	-	-	-
Control 016	0,111	0,427	-	-	-	-	-	-	-	-	0,141	0,526	-	-	-	-	-	-
Control 017	0,112	0,431	-	-	-	-	-	-	-	-	0,175	0,653	0,052	0,867	0,049	0,925	0,071	0,645
Control 018	0,070	0,269	-	-	-	-	-	-	-	-	0,138	0,515	-	-	-	-	-	-
Control 019	0,069	0,265	-	-	-	-	-	-	-	-	0,077	0,287	-	-	-	-	-	-
Control 020	0,078	0,300	-	-	-	-	-	-	-	-	0,077	0,287	-	-	-	-	-	-
Control 021	0,168	0,646	0,114	0,487	0,198	0,588	0,180	0,625	0,198	0,451	0,189	0,705	0,052	0,870	0,046	0,858	0,074	0,673
Control 022	0,082	0,315	-	-	-	-	-	-	-	-	0,085	0,317	-	-	-	-	-	-
Control 023	0,115	0,442	-	-	-	-	-	-	-	-	0,086	0,321	-	-	-	-	-	-
Control 024	0,119	0,458	-	-	-	-	-	-	-	-	0,123	0,459	-	-	-	-	-	-
Control 025	0,100	0,385	-	-	-	-	-	-	-	-	0,141	0,526	-	-	-	-	-	-
Control 026	0,103	0,396	-	-	-	-	-	-	-	-	0,117	0,437	-	-	-	-	-	-
Control 027	0,172	0,662	0,125	0,532	0,189	0,561	0,208	0,722	0,297	0,677	0,122	0,455	-	-	-	-	-	-
Control 028	0,066	0,254	-	-	-	-	-	-	-	-	0,075	0,280	-	-	-	-	-	-
Control 029	0,178	0,685	0,144	0,615	0,095	0,282	0,181	0,627	0,238	0,542	0,191	0,713	0,051	0,850	0,051	0,953	0,106	0,964
Control 030	0,076	0,292	-	-	-	-	-	-	-	-	0,079	0,295	-	-	-	-	-	-
Control 031	0,064	0,246	-	-	-	-	-	-	-	-	0,089	0,332	-	-	-	-	-	-
Control 032	0,076	0,292	-	-	-	-	-	-	-	-	0,098	0,366	-	-	-	-	-	-
Control 033	0,062	0,238	-	-	-	-	-	-	-	-	0,068	0,254	-	-	-	-	-	-
Control 034	0,063	0,242	-	-	-	-	-	-	-	-	0,076	0,284	-	-	-	-	-	-
Control 035	0,128	0,492	-	-	-	-	-	-	-	-	0,116	0,433	-	-	-	-	-	-
Control 036	0,188	0,723	0,171	0,729	0,098	0,291	0,198	0,688	0,098	0,223	0,220	0,821	0,053	0,885	0,049	0,915	0,082	0,745
Control 037	0,119	0,458	-	-	-	-	-	-	-	-	0,125	0,466	-	-	-	-	-	-

	VK247										*P. vivax*-like							
**Sample**	**IgG OD**	**IgG RI**	**IgG1 OD**	**IgG1 RI**	**IgG2 OD**	**IgG2 RI**	**IgG3 OD**	**IgG3 RI**	**IgG4 OD**	**IgG4 RI**	**IgG OD**	**IgG RI**	**IgG1 OD**	**IgG1 RI**	**IgG2 OD**	**IgG2 RI**	**IgG3 OD**	**IgG3 RI**
Control 038	0,129	0,496	-	-	-	-	-	-	-	-	0,121	0,451	-	-	-	-	-	-
Control 039	0,088	0,338	-	-	-	-	-	-	-	-	0,099	0,369	-	-	-	-	-	-
Control 040	0,112	0,431	-	-	-	-	-	-	-	-	0,132	0,493	-	-	-	-	-	-
Control 041	0,193	0,742	0,146	0,624	0,221	0,656	0,116	0,403	0,247	0,563	0,202	0,754	0,057	0,950	0,049	0,925	0,072	0,655
Control 042	0,131	0,504	-	-	-	-	-	-	-	-	0,122	0,455	-	-	-	-	-	-
Control 043	0,135	0,519	-	-	-	-	-	-	-	-	0,078	0,291	-	-	-	-	-	-
Control 044	0,127	0,488	-	-	-	-	-	-	-	-	0,111	0,414	-	-	-	-	-	-
Control 045	0,122	0,469	-	-	-	-	-	-	-	-	0,210	0,784	0,054	0,902	0,051	0,962	0,076	0,691
Control 046	0,097	0,373	-	-	-	-	-	-	-	-	0,136	0,507	-	-	-	-	-	-
Control 047	0,215	0,827	0,172	0,735	0,219	0,650	0,234	0,813	0,232	0,528	0,142	0,530	-	-	-	-	-	-
Control 048	0,116	0,446	-	-	-	-	-	-	-	-	0,138	0,515	-	-	-	-	-	-
Control 049	0,079	0,304	-	-	-	-	-	-	-	-	0,129	0,481	-	-	-	-	-	-
Control 050	0,114	0,438	-	-	-	-	-	-	-	-	0,114	0,425	-	-	-	-	-	-
Control 051	0,129	0,496	-	-	-	-	-	-	-	-	0,117	0,437	-	-	-	-	-	-
Control 052	0,220	0,846	0,190	0,812	0,088	0,261	0,156	0,542	0,094	0,214	0,210	0,784	0,052	0,867	0,048	0,906	0,080	0,727
Control 053	0,126	0,485	-	-	-	-	-	-	-	-	0,089	0,332	-	-	-	-	-	-

			Ct									
Sample	IgG4 OD	IgG4 RI	IgG OD	IgG RI	IgG1 OD	IgG1 RI	IgG2 OD	IgG2 RI	IgG3 OD	IgG3 RI	IgG4 OD	IgG4 RI
CZS001	0,091	1,395	0,307	1,254	0,174	1,079	0,217	0,704	0,190	0,995	0,222	0,781
CZS002	0,051	0,786	0,349	1,424	0,532	3,305	0,260	0,845	0,220	1,152	0,176	0,621
CZS003	-	-	0,289	1,181	0,184	1,141	0,193	0,628	0,210	1,097	0,160	0,563
CZS004	-	-	0,180	0,735	-	-	-	-	-	-	-	-
CZS005	-	-	0,605	2,470	0,311	1,931	1,675	5,437	0,279	1,463	0,403	1,419
CZS006	-	-	0,168	0,686	-	-	-	-	-	-	-	-
CZS007	-	-	0,163	0,664	-	-	-	-	-	-	-	-
CZS008	-	-	0,218	0,888	-	-	-	-	-	-	-	-
CZS009	-	-	0,163	0,667	-	-	-	-	-	-	-	-
CZS010	-	-	0,138	0,562	-	-	-	-	-	-	-	-
CZS011	-	-	0,141	0,577	-	-	-	-	-	-	-	-
CZS012	0,052	0,794	0,357	1,458	0,173	1,073	0,199	0,646	0,137	0,715	0,138	0,487
CZS013	0,053	0,817	0,260	1,062	0,171	1,06	0,237	0,77	0,164	0,861	0,246	0,865
CZS014	0,046	0,709	0,346	1,412	0,154	0,958	0,706	2,293	0,203	1,063	0,169	0,594
CZS015	0,062	0,948	0,268	1,092	0,188	1,169	0,247	0,801	0,197	1,029	0,227	0,798
CZS016	-	-	0,642	2,619	0,157	0,976	0,329	1,067	0,249	1,304	0,160	0,565
CZS017	-	-	0,204	0,832	-	-	-	-	-	-	-	-
CZS018	-	-	0,312	1,275	0,185	1,15	0,265	0,861	0,161	0,843	0,232	0,818
CZS019	0,068	1,048	0,349	1,424	0,222	1,377	0,299	0,972	0,210	1,097	0,323	1,138
CZS020	-	-	0,127	0,518	-	-	-	-	-	-	-	-
CZS021	-	-	0,150	0,611	-	-	-	-	-	-	-	-
CZS022	-	-	0,579	2,362	0,220	1,365	0,529	1,716	0,213	1,113	0,299	1,052
CZS023	0,047	0,717	0,220	0,897	-	-	-	-	-	-	-	-
CZS024	-	-	0,188	0,766	-	-	-	-	-	-	-	-
CZS025	-	-	0,372	1,517	0,156	0,967	0,299	0,972	0,202	1,055	0,149	0,526
CZS026	-	-	0,212	0,866	-	-	-	-	-	-	-	-
CZS027	0,092	1,410	0,194	0,791	-	-	-	-	-	-	-	-
CZS028	-	-	0,303	1,238	0,174	1,079	0,182	0,59	0,189	0,992	0,145	0,512

			Ct									
**Sample**	**IgG4 OD**	**IgG4 RI**	**IgG OD**	**IgG RI**	**IgG1 OD**	**IgG1 RI**	**IgG2 OD**	**IgG2 RI**	**IgG3 OD**	**IgG3 RI**	**IgG4 OD**	**IgG4 RI**
CZS029	-	-	0,185	0,756	-	-	-	-	-	-	-	-
CZS030	-	-	0,157	0,639	-	-	-	-	-	-	-	-
CZS031	0,066	1,010	0,402	1,642	0,136	0,843	0,170	0,552	0,238	1,246	0,243	0,855
CZS032	-	-	0,208	0,850	-	-	-	-	-	-	-	-
CZS033	-	-	0,428	1,747	0,157	0,973	0,294	0,955	0,233	1,22	0,160	0,565
CZS034	0,076	1,171	0,335	1,369	0,255	1,586	0,295	0,959	0,363	1,898	0,284	1,001
CZS035	-	-	0,340	1,387	0,151	0,936	0,312	1,012	0,194	1,016	0,235	0,828
CZS036	0,046	0,709	0,370	1,511	0,157	0,973	0,351	1,139	0,189	0,987	0,132	0,466
CZS037	-	-	0,306	1,247	0,179	1,11	0,217	0,706	0,173	0,908	0,233	0,821
CZS038	-	-	0,205	0,838	-	-	-	-	-	-	-	-
CZS039	0,135	2,073	0,268	1,093	0,146	0,908	0,262	0,85	0,226	1,181	0,219	0,772
CZS040	0,073	1,117	0,300	1,225	0,148	0,92	0,284	0,921	0,271	1,419	0,307	1,082
CZS041	-	-	0,353	1,439	0,220	1,365	0,574	1,864	0,191	1,001	0,179	0,63
CZS042	0,049	0,748	0,211	0,860	-	-	-	-	-	-	-	-
CZS043	-	-	0,283	1,155	0,218	1,352	0,256	0,83	0,273	1,427	0,349	1,229
CZS044	-	-	0,134	0,547	-	-	-	-	-	-	-	-
CZS045	-	-	0,157	0,640	-	-	-	-	-	-	-	-
CZS046	-	-	0,433	1,766	0,410	2,546	0,097	0,315	0,142	0,741	0,110	0,389
CZS047	-	-	0,140	0,571	-	-	-	-	-	-	-	-
CZS048	-	-	0,190	0,776	-	-	-	-	-	-	-	-
CZS049	-	-	0,303	1,235	0,150	0,93	0,284	0,923	0,227	1,191	0,134	0,473
CZS050	-	-	0,137	0,559	-	-	-	-	-	-	-	-
CZS051	-	-	0,161	0,658	-	-	-	-	-	-	-	-
CZS052	-	-	0,125	0,509	-	-	-	-	-	-	-	-
CZS053	-	-	0,168	0,686	-	-	-	-	-	-	-	-
CZS054	-	-	0,195	0,794	-	-	-	-	-	-	-	-
CZS055	0,046	0,701	0,595	2,430	0,129	0,799	0,131	0,425	0,222	1,16	0,145	0,512
CZS056	-	-	0,144	0,587	-	-	-	-	-	-	-	-

			Ct									
Sample	IgG4 OD	IgG4 RI	IgG OD	IgG RI	IgG1 OD	IgG1 RI	IgG2 OD	IgG2 RI	IgG3 OD	IgG3 RI	IgG4 OD	IgG4 RI
CZS057	-	-	0,105	0,428	-	-	-	-	-	-	-	-
CZS058	-	-	0,131	0,534	-	-	-	-	-	-	-	-
CZS059	-	-	0,215	0,878	-	-	-	-	-	-	-	-
CZS060	-	-	0,220	0,897	-	-	-	-	-	-	-	-
CZS061	0,070	1,079	0,134	0,547	-	-	-	-	-	-	-	-
CZS062	-	-	0,185	0,754	-	-	-	-	-	-	-	-
CZS063	-	-	0,137	0,561	-	-	-	-	-	-	-	-
CZS064	-	-	0,189	0,772	-	-	-	-	-	-	-	-
CZS065	-	-	0,111	0,455	-	-	-	-	-	-	-	-
CZS066	0,050	0,771	0,253	1,033	0,195	1,213	0,348	1,129	0,149	0,778	0,175	0,616
CZS067	0,053	0,809	0,266	1,086	0,121	0,752	0,287	0,933	0,192	1,003	0,180	0,635
CZS068	-	-	0,389	1,586	0,119	0,74	0,588	1,909	0,116	0,607	0,199	0,7
CZS069	0,053	0,809	0,210	0,856	-	-	-	-	-	-	-	-
CZS070	-	-	0,203	0,829	-	-	-	-	-	-	-	-
CZS071	0,053	0,817	0,302	1,232	0,143	0,889	0,306	0,993	0,271	1,419	0,169	0,594
CZS072	0,050	0,771	0,407	1,663	0,082	0,507	0,314	1,019	0,086	0,448	0,085	0,301
CZS073	0,064	0,979	0,356	1,455	0,175	1,085	0,156	0,506	0,108	0,568	0,118	0,417
CZS074	0,048	0,740	0,144	0,588	-	-	-	-	-	-	-	-
CZS075	0,193	2,975	0,159	0,651	-	-	-	-	-	-	-	-
CZS076	-	-	0,174	0,710	-	-	-	-	-	-	-	-
CZS077	0,051	0,786	0,233	0,951	-	-	-	-	-	-	-	-
CZS078	0,059	0,902	0,481	1,964	0,135	0,836	0,138	0,448	0,198	1,037	0,188	0,663
CZS079	-	-	0,158	0,645	-	-	-	-	-	-	-	-
CZS080	0,080	1,225	0,135	0,553	-	-	-	-	-	-	-	-
CZS081	-	-	0,186	0,760	-	-	-	-	-	-	-	-
CZS082	-	-	0,187	0,763	-	-	-	-	-	-	-	-
CZS083	-	-	0,101	0,413	-	-	-	-	-	-	-	-
CZS084	-	-	0,214	0,875	-	-	-	-	-	-	-	-

			Ct									
Sample	IgG4 OD	IgG4 RI	IgG OD	IgG RI	IgG1 OD	IgG1 RI	IgG2 OD	IgG2 RI	IgG3 OD	IgG3 RI	IgG4 OD	IgG4 RI
CZS085	-	-	0,190	0,777	-	-	-	-	-	-	-	-
CZS086	0,060	0,925	0,302	1,232	0,119	0,74	0,293	0,952	0,206	1,081	0,519	1,829
CZS087	-	-	0,103	0,419	-	-	-	-	-	-	-	-
CZS088	-	-	0,145	0,590	-	-	-	-	-	-	-	-
CZS089	-	-	0,133	0,543	-	-	-	-	-	-	-	-
CZS090	0,053	0,817	0,198	0,808	-	-	-	-	-	-	-	-
CZS091	-	-	0,209	0,853	-	-	-	-	-	-	-	-
CZS092	0,094	1,441	0,145	0,591	-	-	-	-	-	-	-	-
CZS093	0,048	0,740	0,243	0,992	-	-	-	-	-	-	-	-
CZS094	0,049	0,748	0,476	1,942	0,074	0,46	0,328	1,064	0,091	0,479	0,088	0,31
CZS095	0,052	0,794	0,130	0,529	-	-	-	-	-	-	-	-
CZS096	-	-	0,592	2,418	0,104	0,644	0,089	0,288	0,179	0,935	0,145	0,512
CZS097	-	-	0,205	0,835	-	-	-	-	-	-	-	-
CZS098	-	-	0,180	0,735	-	-	-	-	-	-	-	-
CZS099	-	-	0,179	0,729	-	-	-	-	-	-	-	-
CZS100	-	-	0,115	0,469	-	-	-	-	-	-	-	-
CZS101	0,052	0,801	0,135	0,550	-	-	-	-	-	-	-	-
CZS102	-	-	0,167	0,680	-	-	-	-	-	-	-	-
CZS103	0,046	0,709	0,486	1,983	0,112	0,696	0,331	1,074	0,208	1,089	0,111	0,39
CZS104	0,067	1,033	0,125	0,511	-	-	-	-	-	-	-	-
CZS105	0,051	0,786	0,222	0,906	-	-	-	-	-	-	-	-
CZS106	-	-	0,132	0,537	-	-	-	-	-	-	-	-
CZS107	0,052	0,794	0,082	0,336	-	-	-	-	-	-	-	-
CZS108	-	-	0,167	0,683	-	-	-	-	-	-	-	-
CZS109	0,057	0,871	0,321	1,309	0,101	0,625	0,263	0,853	0,253	1,325	0,109	0,383
CZS110	0,051	0,778	0,553	2,256	0,107	0,665	0,361	1,173	0,146	0,764	0,122	0,429
CZS111	-	-	0,150	0,611	-	-	-	-	-	-	-	-
CZS112	0,101	1,549	0,243	0,993	-	-	-	-	-	-	-	-

			Ct									
**Sample**	**IgG4 OD**	**IgG4 RI**	**IgG OD**	**IgG RI**	**IgG1 OD**	**IgG1 RI**	**IgG2 OD**	**IgG2 RI**	**IgG3 OD**	**IgG3 RI**	**IgG4 OD**	**IgG4 RI**
CZS113	-	-	0,111	0,454	-	-	-	-	-	-	-	-
CZS114	0,046	0,709	0,217	0,884	-	-	-	-	-	-	-	-
CZS115	0,078	1,202	0,176	0,719	-	-	-	-	-	-	-	-
CZS116	-	-	0,148	0,605	-	-	-	-	-	-	-	-
CZS117	0,059	0,909	0,266	1,084	0,177	1,098	0,120	0,388	0,173	0,906	0,141	0,498
CZS118	0,050	0,763	0,563	2,299	0,114	0,706	0,346	1,122	0,151	0,788	0,105	0,369
CZS119	-	-	0,113	0,463	-	-	-	-	-	-	-	-
CZS120	0,047	0,717	0,172	0,701	-	-	-	-	-	-	-	-
CZS121	0,062	0,948	0,420	1,714	0,100	0,619	0,349	1,134	0,141	0,736	0,137	0,482
CZS122	-	-	0,171	0,698	-	-	-	-	-	-	-	-
CZS123	-	-	0,133	0,544	-	-	-	-	-	-	-	-
CZS124	0,053	0,817	0,710	2,898	0,117	0,724	0,353	1,147	0,119	0,623	0,105	0,369
ML001	0,050	0,763	0,176	0,717	-	-	-	-	-	-	-	-
ML002	0,049	0,755	0,214	0,872	-	-	-	-	-	-	-	-
ML003	0,05	0,763	0,139	0,567	-	-	-	-	-	-	-	-
ML004	0,094	1,449	0,469	1,914	0,119	0,737	0,643	2,089	0,220	1,152	0,105	0,368
ML005	-	-	0,175	0,714	-	-	-	-	-	-	-	-
ML006	-	-	0,193	0,788	-	-	-	-	-	-	-	-
ML007	-	-	0,198	0,807	-	-	-	-	-	-	-	-
ML008	0,252	3,884	0,265	1,080	0,115	0,712	0,172	0,56	0,210	1,097	0,101	0,357
ML009	-	-	0,189	0,770	-	-	-	-	-	-	-	-
ML010	0,056	0,855	0,318	1,299	0,091	0,566	0,311	1,009	0,279	1,463	0,112	0,394
ML011	0,052	0,801	0,431	1,760	0,136	0,846	0,364	1,181	0,137	0,715	0,123	0,433
ML012	0,049	0,755	0,169	0,689	-	-	-	-	-	-	-	-
ML013	0,056	0,855	0,654	2,669	0,086	0,532	0,161	0,524	0,203	1,063	0,089	0,315
ML014	0,053	0,817	0,225	0,919	-	-	-	-	-	-	-	-
ML015	-	-	0,234	0,954	-	-	-	-	-	-	-	-
ML016	-	-	0,171	0,698	-	-	-	-	-	-	-	-

			Ct									
Sample	IgG4 OD	IgG4 RI	IgG OD	IgG RI	IgG1 OD	IgG1 RI	IgG2 OD	IgG2 RI	IgG3 OD	IgG3 RI	IgG4 OD	IgG4 RI
ML017	0,055	0,848	0,261	1,064	0,091	0,566	0,140	0,456	0,197	1,029	0,087	0,306
ML018	-	-	0,189	0,773	-	-	-	-	-	-	-	-
ML019	-	-	0,202	0,825	-	-	-	-	-	-	-	-
ML020	0,052	0,794	0,392	1,600	0,098	0,606	0,160	0,521	0,249	1,304	0,093	0,327
ML021	-	-	0,211	0,860	-	-	-	-	-	-	-	-
ML022	0,052	0,801	0,411	1,678	0,113	0,703	0,422	1,369	0,161	0,843	0,123	0,433
ML023	-	-	0,182	0,742	-	-	-	-	-	-	-	-
ML024	-	-	0,157	0,642	-	-	-	-	-	-	-	-
ML025	-	-	0,112	0,456	-	-	-	-	-	-	-	-
ML026	0,056	0,855	0,338	1,381	0,184	1,141	0,168	0,547	0,210	1,097	0,116	0,41
ML027	0,052	0,794	0,604	2,465	0,138	0,858	0,338	1,098	0,213	1,113	0,113	0,397
ML028	0,115	1,765	0,376	1,536	0,231	1,436	0,140	0,454	0,202	1,055	0,116	0,41
ML029	0,226	3,483	0,211	0,862	-	-	-	-	-	-	-	-
ML030	-	-	0,170	0,695	-	-	-	-	-	-	-	-
ML031	0,054	0,825	0,327	1,336	0,097	0,603	0,121	0,393	0,238	1,246	0,085	0,299
ML032	-	-	0,156	0,635	-	-	-	-	-	-	-	-
ML033	0,054	0,832	0,383	1,564	0,115	0,715	0,172	0,558	0,233	1,22	0,108	0,38
ML034	0,415	6,381	0,552	2,253	0,115	0,712	0,404	1,312	0,363	1,898	0,113	0,399
ML035	-	-	0,178	0,726	-	-	-	-	-	-	-	-
ML036	0,066	1,017	0,332	1,357	0,213	1,325	0,120	0,388	0,194	1,016	0,135	0,477
ML037	0,053	0,809	0,362	1,477	0,127	0,789	0,106	0,344	0,198	1,037	0,099	0,348
ML038	-	-	0,473	1,929	0,090	0,557	0,393	1,275	0,173	0,908	0,107	0,375
ML039	0,204	3,144	0,236	0,965	-	-	-	-	-	-	-	-
ML040	0,060	0,917	0,534	2,180	0,099	0,616	0,327	1,062	0,226	1,181	0,097	0,343
ML041	0,050	0,771	0,256	1,043	0,098	0,609	0,117	0,38	0,271	1,419	0,078	0,276
ML042	0,051	0,786	0,473	1,932	0,081	0,501	0,122	0,397	0,191	1,001	0,090	0,317
ML043	0,053	0,817	0,398	1,624	0,107	0,662	0,157	0,509	0,273	1,427	0,131	0,461
ML044	0,059	0,902	0,164	0,671	-	-	-	-	-	-	-	-

			Ct									
**Sample**	**IgG4 OD**	**IgG4 RI**	**IgG OD**	**IgG RI**	**IgG1 OD**	**IgG1 RI**	**IgG2 OD**	**IgG2 RI**	**IgG3 OD**	**IgG3 RI**	**IgG4 OD**	**IgG4 RI**
ML045	0,050	0,763	0,137	0,561	-	-	-	-	-	-	-	-
ML046	-	-	0,443	1,807	0,088	0,544	0,363	1,178	0,227	1,191	0,109	0,385
ML047	0,051	0,786	0,154	0,627	-	-	-	-	-	-	-	-
ML048	0,058	0,886	0,344	1,406	0,087	0,541	0,191	0,621	0,222	1,16	0,140	0,494
ML049	-	-	0,112	0,459	-	-	-	-	-	-	-	-
ML050	0,050	0,763	0,163	0,665	-	-	-	-	-	-	-	-
ML051	-	-	0,125	0,509	-	-	-	-	-	-	-	-
ML052	0,054	0,825	0,154	0,630	-	-	-	-	-	-	-	-
ML053	0,061	0,940	0,189	0,772	-	-	-	-	-	-	-	-
ML054	0,052	0,801	0,159	0,651	-	-	-	-	-	-	-	-
ML055	-	-	0,174	0,711	-	-	-	-	-	-	-	-
ML056	-	-	0,129	0,528	-	-	-	-	-	-	-	-
ML057	-	-	0,214	0,872	-	-	-	-	-	-	-	-
ML058	-	-	0,557	2,275	0,182	1,129	0,257	0,834	0,192	1,003	0,098	0,346
ML059	-	-	0,193	0,788	-	-	-	-	-	-	-	-
ML060	-	-	0,147	0,599	-	-	-	-	-	-	-	-
ML061	-	-	0,164	0,670	-	-	-	-	-	-	-	-
ML062	0,048	0,740	1,122	4,580	0,104	0,647	0,646	2,096	0,116	0,607	0,135	0,475
ML063	-	-	0,135	0,553	-	-	-	-	-	-	-	-
ML064	0,045	0,686	0,490	2,001	0,100	0,619	0,474	1,538	0,271	1,419	0,103	0,362
ML065	-	-	0,208	0,847	-	-	-	-	-	-	-	-
ML066	-	-	0,687	2,804	0,115	0,715	0,554	1,8	0,108	0,568	0,100	0,353
ML067	-	-	0,116	0,472	-	-	-	-	-	-	-	-
ML068	-	-	0,137	0,561	-	-	-	-	-	-	-	-
ML069	-	-	0,532	2,173	0,174	1,082	0,254	0,826	0,139	0,728	0,165	0,582
ML070	-	-	0,122	0,499	-	-	-	-	-	-	-	-
ML071	-	-	0,108	0,441	-	-	-	-	-	-	-	-
ML072	-	-	0,119	0,487	-	-	-	-	-	-	-	-

			Ct									
Sample	IgG4 OD	IgG4 RI	IgG OD	IgG RI	IgG1 OD	IgG1 RI	IgG2 OD	IgG2 RI	IgG3 OD	IgG3 RI	IgG4 OD	IgG4 RI
ML073	-	-	0,183	0,748	-	-	-	-	-	-	-	-
ML074	-	-	0,243	0,992	-	-	-	-	-	-	-	-
ML075	0,045	0,686	0,138	0,565	-	-	-	-	-	-	-	-
ML076	0,048	0,740	0,207	0,844	-	-	-	-	-	-	-	-
ML077	-	-	0,151	0,618	-	-	-	-	-	-	-	-
ML078	-	-	0,147	0,599	-	-	-	-	-	-	-	-
ML079	-	-	0,140	0,570	-	-	-	-	-	-	-	-
ML080	-	-	0,253	1,032	0,153	0,948	0,167	0,542	0,206	1,081	0,135	0,475
ML081	-	-	0,154	0,627	-	-	-	-	-	-	-	-
ML082	-	-	0,101	0,413	-	-	-	-	-	-	-	-
ML083	-	-	0,221	0,900	-	-	-	-	-	-	-	-
ML084	-	-	0,133	0,541	-	-	-	-	-	-	-	-
ML085	-	-	0,134	0,547	-	-	-	-	-	-	-	-
ML086	0,081	1,241	0,127	0,517	-	-	-	-	-	-	-	-
ML087	0,070	1,079	0,206	0,841	-	-	-	-	-	-	-	-
ML088	0,171	2,628	0,333	1,361	0,149	0,923	0,310	1,007	0,176	0,919	0,206	0,725
GJ001	0,058	0,886	0,170	0,693	-	-	-	-	-	-	-	-
GJ002	0,056	0,863	0,158	0,645	-	-	-	-	-	-	-	-
GJ003	0,048	0,732	0,142	0,579	-	-	-	-	-	-	-	-
GJ004	-	-	0,329	1,341	0,124	0,768	0,416	1,351	0,185	0,966	0,118	0,415
GJ005	-	-	0,128	0,524	-	-	-	-	-	-	-	-
GJ006	0,093	1,433	0,258	1,052	0,109	0,675	0,491	1,594	0,122	0,641	0,122	0,431
GJ007	0,048	0,732	0,369	1,505	0,111	0,69	0,407	1,323	0,108	0,568	0,128	0,45
GJ008	0,048	0,740	0,138	0,564	-	-	-	-	-	-	-	-
GJ009	-	-	0,173	0,708	-	-	-	-	-	-	-	-
GJ010	-	-	0,132	0,540	-	-	-	-	-	-	-	-
GJ011	-	-	0,155	0,632	-	-	-	-	-	-	-	-
GJ012	-	-	0,196	0,798	-	-	-	-	-	-	-	-

			Ct									
Sample	IgG4 OD	IgG4 RI	IgG OD	IgG RI	IgG1 OD	IgG1 RI	IgG2 OD	IgG2 RI	IgG3 OD	IgG3 RI	IgG4 OD	IgG4 RI
GJ013	-	-	0,179	0,732	-	-	-	-	-	-	-	-
GJ014	-	-	0,183	0,748	-	-	-	-	-	-	-	-
GJ015	0,057	0,879	0,211	0,863	-	-	-	-	-	-	-	-
GJ016	0,064	0,986	0,344	1,406	0,116	0,721	0,257	0,835	0,231	1,207	0,115	0,406
GJ017	-	-	0,182	0,742	-	-	-	-	-	-	-	-
GJ018	0,062	0,956	0,140	0,570	-	-	-	-	-	-	-	-
GJ019	-	-	0,182	0,741	-	-	-	-	-	-	-	-
GJ020	-	-	0,131	0,534	-	-	-	-	-	-	-	-
GJ021	0,050	0,771	0,275	1,123	0,087	0,541	0,683	2,216	0,047	0,244	0,099	0,348
GJ022	0,065	1,002	0,452	1,843	0,118	0,731	0,373	1,212	0,107	0,558	0,110	0,389
GJ023	0,062	0,948	0,135	0,550	-	-	-	-	-	-	-	-
GJ024	0,099	1,518	0,774	3,159	0,107	0,662	0,345	1,121	0,197	1,031	0,129	0,455
GJ025	0,051	0,786	0,174	0,710	-	-	-	-	-	-	-	-
GJ026	0,053	0,809	0,175	0,716	-	-	-	-	-	-	-	-
GJ027	0,060	0,925	0,111	0,455	-	-	-	-	-	-	-	-
GJ028	0,059	0,902	0,214	0,873	-	-	-	-	-	-	-	-
GJ029	-	-	0,196	0,801	-	-	-	-	-	-	-	-
GJ030	-	-	0,115	0,471	-	-	-	-	-	-	-	-
GJ031	0,067	1,033	0,379	1,545	0,137	0,852	0,162	0,526	0,219	1,149	0,152	0,535
GJ032	-	-	0,099	0,404	-	-	-	-	-	-	-	-
GJ033	0,052	0,794	0,801	3,271	0,117	0,724	1,594	5,174	0,167	0,872	0,250	0,881
GJ034	0,052	0,794	0,145	0,591	-	-	-	-	-	-	-	-
GJ035	0,065	1,002	0,310	1,266	0,135	0,839	0,146	0,475	0,243	1,272	0,230	0,809
GJ036	0,052	0,801	0,871	3,556	0,123	0,765	0,347	1,127	0,164	0,859	0,207	0,73
GJ037	-	-	0,163	0,667	-	-	-	-	-	-	-	-
GJ038	-	-	0,097	0,397	-	-	-	-	-	-	-	-
GJ039	0,056	0,863	0,126	0,514	-	-	-	-	-	-	-	-
GJ040	-	-	0,102	0,416	-	-	-	-	-	-	-	-

			Ct									
Sample	IgG4 OD	IgG4 RI	IgG OD	IgG RI	IgG1 OD	IgG1 RI	IgG2 OD	IgG2 RI	IgG3 OD	IgG3 RI	IgG4 OD	IgG4 RI
GJ041	-	-	0,662	2,703	0,095	0,588	0,399	1,294	0,145	0,757	0,127	0,448
GJ042	-	-	0,179	0,729	-	-	-	-	-	-	-	-
GJ043	0,052	0,801	0,386	1,576	0,095	0,588	0,238	0,774	0,213	1,113	0,143	0,503
GJ044	0,065	0,994	0,372	1,517	0,129	0,802	0,190	0,616	0,208	1,089	0,199	0,702
GJ045	0,054	0,825	0,875	3,573	0,132	0,821	0,542	1,76	0,164	0,861	0,177	0,623
GJ046	0,051	0,778	0,341	1,390	0,101	0,628	0,378	1,228	0,158	0,827	0,162	0,572
GJ047	-	-	0,145	0,591	-	-	-	-	-	-	-	-
GJ048	0,071	1,087	0,125	0,511	-	-	-	-	-	-	-	-
GJ049	-	-	0,147	0,602	-	-	-	-	-	-	-	-
GJ050	-	-	0,103	0,419	-	-	-	-	-	-	-	-
GJ051	-	-	0,201	0,822	-	-	-	-	-	-	-	-
GJ052	0,050	0,763	0,090	0,368	-	-	-	-	-	-	-	-
GJ053	0,068	1,048	0,586	2,393	0,104	0,647	1,431	4,645	0,168	0,877	0,605	2,13
GJ054	-	-	0,174	0,712	-	-	-	-	-	-	-	-
GJ055	-	-	0,173	0,707	-	-	-	-	-	-	-	-
GJ056	-	-	0,128	0,524	-	-	-	-	-	-	-	-
GJ057	-	-	0,158	0,645	-	-	-	-	-	-	-	-
GJ058	-	-	0,148	0,605	-	-	-	-	-	-	-	-
GJ059	0,059	0,902	0,151	0,618	-	-	-	-	-	-	-	-
GJ060	-	-	0,146	0,597	-	-	-	-	-	-	-	-
GJ061	-	-	0,154	0,627	-	-	-	-	-	-	-	-
GJ062	-	-	0,166	0,676	-	-	-	-	-	-	-	-
GJ063	-	-	0,179	0,729	-	-	-	-	-	-	-	-
GJ064	-	-	0,191	0,779	-	-	-	-	-	-	-	-
GJ065	0,055	0,848	0,442	1,805	0,121	0,752	0,210	0,683	0,221	1,155	0,131	0,463
GJ066	-	-	0,207	0,844	-	-	-	-	-	-	-	-
GJ067	0,048	0,740	0,159	0,651	-	-	-	-	-	-	-	-
GJ068	0,050	0,763	0,429	1,750	0,104	0,647	0,945	3,067	0,159	0,83	0,221	0,779

			Ct									
Sample	IgG4 OD	IgG4 RI	IgG OD	IgG RI	IgG1 OD	IgG1 RI	IgG2 OD	IgG2 RI	IgG3 OD	IgG3 RI	IgG4 OD	IgG4 RI
GJ069	0,047	0,724	0,093	0,380	-	-	-	-	-	-	-	-
GJ070	0,060	0,917	0,265	1,083	0,097	0,6	0,240	0,78	0,210	1,1	0,108	0,382
GJ071	-	-	0,187	0,763	-	-	-	-	-	-	-	-
GJ072	-	-	0,157	0,639	-	-	-	-	-	-	-	-
GJ073	0,050	0,771	0,113	0,463	-	-	-	-	-	-	-	-
GJ074	0,054	0,825	0,142	0,579	-	-	-	-	-	-	-	-
GJ075	0,048	0,740	0,130	0,529	-	-	-	-	-	-	-	-
GJ076	0,054	0,832	0,162	0,660	-	-	-	-	-	-	-	-
GJ077	0,046	0,701	0,141	0,576	-	-	-	-	-	-	-	-
GJ078	-	-	0,127	0,520	-	-	-	-	-	-	-	-
GJ079	0,048	0,740	0,438	1,789	0,106	0,656	0,561	1,823	0,218	1,139	0,097	0,343
GJ080	0,075	1,156	0,369	1,506	0,130	0,805	0,468	1,52	0,146	0,762	0,250	0,879
GJ081	0,056	0,855	0,305	1,244	0,124	0,768	0,335	1,087	0,156	0,819	0,160	0,565
GJ082	0,060	0,917	0,172	0,701	-	-	-	-	-	-	-	-
GJ083	0,068	1,040	0,363	1,481	0,174	1,079	0,452	1,466	0,141	0,738	0,174	0,612
GJ084	0,064	0,986	0,383	1,562	0,140	0,871	0,717	2,329	0,159	0,83	0,236	0,830
GJ085	0,089	1,372	0,682	2,783	0,318	1,977	0,314	1,019	0,226	1,183	0,139	0,489
GJ086	0,083	1,279	0,457	1,864	0,118	0,731	0,300	0,973	0,216	1,131	0,144	0,506
GJ087	0,068	1,040	0,196	0,799	-	-	-	-	-	-	-	-
Control 001	-	-	0,087	0,355	-	-	-	-	-	-	-	-
Control 002	-	-	0,090	0,367	-	-	-	-	-	-	-	-
Control 003	-	-	0,125	0,510	-	-	-	-	-	-	-	-
Control 004	0,054	0,831	0,129	0,527	0,143	0,888	0,096	0,312	0,137	0,717	0,101	0,356
Control 005	-	-	0,122	0,498	-	-	-	-	-	-	-	-
Control 006	-	-	0,137	0,559	0,134	0,832	0,106	0,344	0,128	0,67	0,147	0,518
Control 007	0,055	0,846	0,104	0,424	-	-	-	-	-	-	-	-
Control 008	-	-	0,114	0,465	-	-	-	-	-	-	-	-
Control 009	-	-	0,156	0,637	0,117	0,727	0,147	0,477	0,132	0,691	0,134	0,472

			Ct									
Sample	IgG4 OD	IgG4 RI	IgG OD	IgG RI	IgG1 OD	IgG1 RI	IgG2 OD	IgG2 RI	IgG3 OD	IgG3 RI	IgG4 OD	IgG4 RI
Control 010	0,053	0,815	0,131	0,535	0,138	0,857	0,164	0,532	0,145	0,759	0,235	0,827
Control 011	-	-	0,102	0,416	-	-	-	-	-	-	-	-
Control 012	-	-	0,092	0,376	-	-	-	-	-	-	-	-
Control 013	-	-	0,196	0,800	0,105	0,652	0,229	0,744	0,136	0,712	0,148	0,521
Control 014	-	-	0,080	0,327	-	-	-	-	-	-	-	-
Control 015	-	-	0,088	0,359	-	-	-	-	-	-	-	-
Control 016	-	-	0,089	0,363	-	-	-	-	-	-	-	-
Control 017	0,054	0,823	0,093	0,380	-	-	-	-	-	-	-	-
Control 018	-	-	0,127	0,518	-	-	-	-	-	-	-	-
Control 019	-	-	0,121	0,494	-	-	-	-	-	-	-	-
Control 020	-	-	0,148	0,604	0,127	0,789	0,092	0,299	0,182	0,953	0,221	0,778
Control 021	0,061	0,938	0,125	0,510	-	-	-	-	-	-	-	-
Control 022	-	-	0,072	0,294	-	-	-	-	-	-	-	-
Control 023	-	-	0,071	0,290	-	-	-	-	-	-	-	-
Control 024	-	-	0,095	0,388	-	-	-	-	-	-	-	-
Control 025	-	-	0,09	0,367	-	-	-	-	-	-	-	-
Control 026	-	-	0,067	0,273	-	-	-	-	-	-	-	-
Control 027	-	-	0,068	0,278	-	-	-	-	-	-	-	-
Control 028	-	-	0,189	0,771	0,121	0,752	0,161	0,523	0,138	0,723	0,162	0,57
Control 029	0,052	0,800	0,183	0,747	0,128	0,795	0,159	0,516	0,119	0,623	0,123	0,433
Control 030	-	-	0,112	0,457	-	-	-	-	-	-	-	-
Control 031	-	-	0,104	0,424	-	-	-	-	-	-	-	-
Control 032	-	-	0,121	0,494	-	-	-	-	-	-	-	-
Control 033	-	-	0,116	0,473	-	-	-	-	-	-	-	-
Control 034	-	-	0,078	0,318	-	-	-	-	-	-	-	-
Control 035	-	-	0,066	0,269	-	-	-	-	-	-	-	-
Control 036	0,053	0,815	0,114	0,465	-	-	-	-	-	-	-	-
Control 037	-	-	0,121	0,494	-	-	-	-	-	-	-	-

			Ct									
Sample	IgG4 OD	IgG4 RI	IgG OD	IgG RI	IgG1 OD	IgG1 RI	IgG2 OD	IgG2 RI	IgG3 OD	IgG3 RI	IgG4 OD	IgG4 RI
Control 038	-	-	0,090	0,367	-	-	-	-	-	-	-	-
Control 039	-	-	0,078	0,318	-	-	-	-	-	-	-	-
Control 040	-	-	0,084	0,343	-	-	-	-	-	-	-	-
Control 041	0,052	0,792	0,182	0,743	0,133	0,826	0,086	0,279	0,139	0,728	0,151	0,532
Control 042	-	-	0,104	0,424	-	-	-	-	-	-	-	-
Control 043	-	-	0,111	0,453	-	-	-	-	-	-	-	-
Control 044	-	-	0,089	0,363	-	-	-	-	-	-	-	-
Control 045	0,058	0,892	0,076	0,310	-	-	-	-	-	-	-	-
Control 046	-	-	0,078	0,318	-	-	-	-	-	-	-	-
Control 047	-	-	0,118	0,482	-	-	-	-	-	-	-	-
Control 048	-	-	0,121	0,494	-	-	-	-	-	-	-	-
Control 049	-	-	0,123	0,502	-	-	-	-	-	-	-	-
Control 050	-	-	0,098	0,400	-	-	-	-	-	-	-	-
Control 051	-	-	0,114	0,465	-	-	-	-	-	-	-	-
Control 052	0,060	0,923	0,192	0,784	0,114	0,708	0,233	0,756	0,15	0,785	0,112	0,394
Control 053	-	-	0,112	0,457	-	-	-	-	-	-	-	-

Supplementary Data: Dataset of immune response and population exposure.
